# Identification of NLE1/CDK1 axis as key regulator in the development and progression of non-small cell lung cancer

**DOI:** 10.3389/fonc.2022.985827

**Published:** 2023-02-01

**Authors:** Pei Xu, Lei Wang, Bin Mo, Xiao Xie, Rui Hu, Lianyong Jiang, Fengqing Hu, Fangbao Ding, Haibo Xiao

**Affiliations:** Department of Cardiothoracic Surgery, Xinhua Hospital Affiliated to Shanghai Jiao Tong University School of Medicine, Shanghai, China

**Keywords:** non-small cell lung cancer, NLE1, tumor promotor, molecular mechanism, CDK1

## Abstract

Non-small cell lung cancer (NSCLC) is the most common pathological type of lung cancer, which is a severer threaten to human health because of its extremely high morbidity and mortality. In this study, the role of Notchless homolog 1 (NLE1) in the development of NSCLC was investigated and the underlying mechanism was explored. The outcomes showed that NLE1 expression is significantly higher in tumor tissues than normal tissues, and is correlated with the pathological stage. The regulation of NSCLC development by NLE1 was also visualized by the *in vitro* and *in vivo* loss-of-function studies, which indicated the inhibition of cell growth and migration, as well as enhancement of cell apoptosis on condition of NLE1 knockdown. As for the mechanism, it was demonstrated that NLE1 may execute its tumor-regulating function through activating E2F1-mediated transcription of CDK1, and PI3K/Akt signaling pathway was also supposed as a downstream of NLE1 in the regulation of NSCLC. Both CDK1 overexpression and treatment of Akt pathway activator could reverse the NLE1 knockdown induced NSCLC inhibition to some extent. In conclusion, this study identified NLE1 as a novel tumor promotor in the development and progression of NSCLC, which may be a potential therapeutic target in the treatment of NSCLC.

## 1 Introduction

Worldwide, malignant tumors have become one of the most serious public health problems. Among all types of cancers, lung cancer possesses the highest incidence rate and mortality, and they are still on the rise in recent years (1, 2). Non-small cell lung cancer (NSCLC) is the most common pathological type of lung cancer. Due to its rapid development and lack of specific early symptoms, more than 60% of NSCLC patients have been in the middle or late stage of the disease at the time of diagnosis, and lost the opportunity of surgical treatment, which is currently still the most effective means for treating lung cancer (3, 4). Previously, the major treatment strategy of lung cancer was chemotherapy with platinum-containing cytotoxic drugs, but the response rate was only 20-35% with a median survival time of 10-12 months (4, 5). In the past decades, more and more research found that the occurrence and development of NSCLC has very significant molecular characteristics, which stimulates the development of molecular targeted therapy and brings a significant change to the treatment of lung cancer (6, 7). However, due to the heterogeneity of tumor cells and the instability of genome, the efficacy of single targeted drug will be reduced rapidly due to the emergence of drug resistance (8). Therefore, it is of great significance to explore more regulatory factors that play a key role in the development and progression of lung cancer in order to develop more targeted drugs and solve the treatment dilemma of some lung cancer patients.

The Notch signaling pathway is a highly evolutionarily conserved signal transduction pathway, which is involved in mediating cell differentiation, proliferation and angiogenesis, and plays an important role in embryonic development and maintaining tissue homeostasis (9–11). Recently, more and more studies have shown that the abnormality of the Notch signaling pathway is closely related to the differentiation, proliferation, apoptosis of tumor cells, and the epithelial-mesenchymal transition, thus participating in the occurrence, development, invasion and metastasis of a variety of tumors (12). In mammals, Notch signal transduction is composed of 4 types of heterodimers of Notch receptors (Notch1-4), 5 types of type I transmembrane proteins, and DNA binding protein, of which Notch1 is one of the most focused ligands (13). Mutations of Notch1 can cause ligand-independent activation, leading to the continuous activation of Notch1 signaling pathway (13, 14). In view of the in-depth research on the role of Notch signaling pathway in tumorigenesis and development, there are more and more research on the regulatory mechanism of Notch signaling pathway and existing studies have identified some direct regulators of the Notch signaling pathway (15). For example, Notchless homolog 1 (NLE1), as a regulator of Notch1, was identified through a genetic screen for suppressors of the notchoid mutant in drosophila (16–18). However, although the role of Notch signaling pathway in cancer has been comprehensively investigated, the relationship between human cancer and NLE1 was rarely reported and mains largely unknown. Only few studies concerning malignant melanoma and colon adenocarcinoma indicated that NLE1 may have certain potential in the regulation of tumor growth and prediction of patients’ prognosis (19, 20).

Bearing all these in mind, in this study, we explored the expression pattern and functions of NLE1 in NSCLC. First of all, the expression of NLE1 in lung cancer tissues and para-carcinoma tissues was investigated and statistically analyzed in a combination of tumor characteristics and patients’ survival. Subsequently, functionally, a NLE1 knockdown cell model was constructed for conducting loss-of-function studies through examining cell phenotypes. Moreover, the downstream factor of NLE1 in the regulation of NSCLC was screened by a gene microarray and recognize CDK1 as a potential molecular target of NLE1. Therefore, our study demonstrated the tumor-promoting role of NLE1 in NSCLC which may be mediated by CDK1.

## 2 Materials and methods

### 2.1 Cell culture

Human lung cancer cell lines A549, NCI-H1299 and SPC-A-1 were purchased from Cell resource center, Shanghai Institute of life sciences, Chinese Academy of Sciences (Shanghai, China) and EBC-1 was obtained from Mingzhou Biotechnology Co., Ltd (Ningbo, Zhejiang, China). A549 was cultured in McCoy’s 5A Media (Modified with Tricine) with 10% FBS, and NCI-H1299, SPC-A-1 and EBC-1 cell lines were grown in RPMI (w/o Hepes) Media containing 10% FBS. All cells were cultured in a CO_2_ cell incubator (Thermo) at 37°C. For cell infection by lentivirus, cells were seeded in a six-well plate, and 20 mL 1×10^8^ TU/mL lentivirus were added, along with ENI.S and Polybrene for culturing. After 72 h, cell infection efficiency was valued by observing GFP fluorescence under a microscope.

### 2.2 Immunohistochemistry (IHC) analysis

Lung cancer tissues and adjacent normal tissues contained in the formalin fixed and paraffin embedded tissue microarray (Lot No. HLug-Squ150Sur-02, made in Shanghai Outdo Biotech Company) was analyzed here. Firstly, the microarray was baked in an oven at 65°C for 30 min. Next, the microarray was deparaffinized in analytically pure xylene and rehydrated in 100% to 75% alcohol. After digested with citric acid solution, the microarray was blocked with 3% H_2_O_2_. Next, the microarray was incubated with NLE1 antibody and the second biotinylated antibody (NLE1, 1:100, ab230212, Abcam). DAB staining solution and Hematoxylin solution was applied for staining. NLE1 expressing levels were revealed by the staining intensity and positive rate. Staining intensity is scored as: negative = zero score, Light yellow = one score, brown yellow = two scores, dark brown= three scores, and positive rate is scored as: zero-0% = zero score, <25% = one score, 25-50% = two scores, 50-75% = three scores, or >75% = four scores. This study was approved by Xinhua Ethics committee affiliated to Shanghai Tongji University School of Medicine (Approval number: XHEC-NSFC-2021-242).

### 2.3 Lentiviral vector construction

siRNA of NLE1 was designed and three target interference sequences were as follow: 5’-AAGGACAAATGCCTCCGGATA-3’, 5’-CCTTGCAGGAGTTGAAGGAGA-3’, 5’-ACTCGGATGACAGGACACCAA-3’. Short hairpin RNA (shRNA) sequences were constructed by adding suitable restriction endonuclease site (CCGG: Agei site; AATTC: EcoRI site). Next, RNA interference lentiviral vector was constructed using Fermentas T4 DNA Ligase and competent escherichia coli cells. shRNA sequences expressing cell clones were identified by PCR. Then positive cells were cultured for plasmids extracting. Qualified plasmids were used for lentiviral packaging.

### 2.4 qRT-PCR

RNA for PCR was isolated using Trizol (Sigma). The concentration and quality of RNA were evaluated by Nanodrop 2000/2000c. Real-time PCR was performed to quantify the expression of genes. GAPDH act as inner control. The primers were used as followed: NLE1 Forward: 5’-TCTGAGCCGATACAACCTCG -3′,

NLE1 Reverse: 5′-GGGGACCACAGGAATAAGGTGA-3′;

GAPDH Forward: 5’-TGACTTCAACAGCGACACCCA -3′,

GAPDH Reverse: 5’-CACCCTGTTGCTGTAGCCAAA-3′;

CDK1 Forward: 5’-CCATACCCATTGACTAACTAT-3′,

CDK1 Reverse: 5’-ACCCCTTCCTCTTCACTTTC-3′.

### 2.5 Western blot (WB) assay and co-immunoprecipitation (Co-IP) assay

Total protein was extracted using IP cell lysate (Servicebio) containing PMSF (100:1), and the concentration and quality of total protein was detected by BCA Protein Assay Kit (HyClone-Pierce). Equal amounts of total protein (20 μg/lane) were separated by 10% SDS-polyacrylamide gel, and transferred to a polyvinylidene difluoride (PVDF) membrane and subjected to WB analysis. Antibodies used as follows: NLE1 (1:1000, # orb157995, biorbyt); CDK1 (1:3000, #ab133327, Abcam); ERK (1:2000, #4695, CST); P-ERK (1:500, #4370, CST); mTOR (1:2000, #ab2732, Abcam); P-mTOR (1:1000, #ab2732, Abcam); CDK6 (1:1000, #ab151247, Abcam); PIK3CA (1:1000, #ab40776, Abcam); AKT (1:2000, #10176-2-AP, Proteintech); p-AKT (1:1000, #10176-2-AP, Proteintech); PI3K (1:500; #AF2998-SP, Systems); p-PI3K (1:1000, #ab182651, Abcam); GAPDH (1:3000, #AP0063, Bioworld), BRCA1 (1:1000, #ab191042, Abcam); CCNE2 (1:2000, #ab40890, Abcam); PLK(1:1000, #ab17057, Abcam); Goat Anti-Rabbit (1:3000, #A0208, Beyotime), Goat Anti-Mouse (1:3000, #A0216, Beyotime), Donkey Anti-Goat (1:3000, #A0181, Beyotime).

Co-IP assay was used to detect the interaction of target proteins in cells. 1.0 mg protein samples and antibodies were incubated at 4°C overnight by rotating, following incubated with 20 μL beads at 4°C for 2 h. After centrifugal and washing for three times, samples were incubated in IP lysate and loading buffer at 100°C for 5 min. Protein was separated by SDS-PAGE and protein amount was determined by WB assay.

### 2.6 Cell proliferation

Cell proliferation was conducted by MTT assay, CCK-8 assay and Celigo cell counting assay. Lentivirus infected cells were seeded into five 96-well plates with about 2000 or 3000 cells per well and cultured in a cell incubator. 20 μL 5 mg/mL MTT (Geneview) was added 4 h before the end of culturing. 4 h later, culture medium was removed and 100μL DMSO was added and oscillated for 5 min. For CCK-8 assay, 10 μL 5 mg/mL CCK-8 reagent (Sigma) was added each well for 4 h. OD value was measured with a microplate reader (Tecan Infinite) for continuing five days.

For Celigo cell counting assay, cells were seeded in 96-well plates and plates were scanned by Celigo Image Cytometer (Nexcelom) for continuing five days. The cell number curve changed with time was drawn.

### 2.7 Migration assay

Cell migration was performed by Corning Transwell chamber and Wound healing assay. Cells (5 × 10^4^ cell/well) were seeded into a 12-well cell culture insert which contains 8 μm polycarbonate membrane serum free medium. 30% FBS culture medium was added into the lower chamber. After 16 h culturing, cells migrate and adhere to the bottom of polycarbonate membrane were stained and pictures were obtained with a microscope (Olympus).

For wound healing assay, cells were seeded into a 96-well plate until the cell confluence up to 90%. Wounds across each cell layer was accomplished by a 96-Wounding Replicator (VP Scientific) and cell debris were washed. Cells were cultured in 0.5% FBS culture medium and photos were captured 24 h and 72 h port wounding under a fluorescence microscope.

### 2.8 Flow cytometry assay

Lentivirus infected cells were cultured in a 6-well plate for 5 days with a cell confluence reached about 85%, then the cells were collected and washed with D-hanks (pH=7.2~7.4) on ice. Then, cells were suspended with 200 μL 1 × binding buffer. To detect cell apoptosis, cells were stained by Annexin V-APC staining kit (eBioscience). To detect cell cycle, cells were fixed with 4°C 70% ethanol for at least 1 h, after washing, cells were suspended with PI solution (Sigma). Cell apoptosis or cells phases were analyzed under a flow cytometry (Millipore).

### 2.9 Colony formation

After lentivirus infection, cells were continuing cultured 10 days for colony forming. Colonies were fixed with 4% paraformaldehyde (Sigma) and stained with Giemsa. Colony number was counted finally.

### 2.10 GeneChip analysis

RNA from infected NCI-H1299 cells were collected using Trizol reagent, and RNA concentration was detected by Nanodrop 2000. Qualified RNA samples (1.7< A260/A280 <2.2) were used for amplification and labeling using Affymetrix 3’ IVT Plus Kit. After hybridization, raw data was collected for data filtering using Bayesian and Benjamini-Hochberg (B-H) method (|Fold Change| ≥ 1.5 and FDR<0.05), the remaining data were analyzed for significant difference analysis and functional analysis of differential genes. The differential genes in the gene chip were directly used for KEGG pathway enrichment analysis in https://david.ncifcrf.govhome.jsp.

### 2.11 Human apoptosis antibody array

Briefly, NCI-H1299 cellular extracts are diluted and incubated with the Human Apoptosis Array (#ab134001, Abcam) overnight. The array is washed to remove unbound proteins, followed by incubation with a cocktail of biotinylated detection antibodies. Streptavidin-HRP and chemiluminescent detection reagents are applied as a WB assay, and signals were produced at capture spots corresponding to the amount of protein bound.

### 2.12 Chromatin immunoprecipitation (ChIP)-qPCR assay

SimpleChIP^®^ Enzymatic Chromatin IP Kit (#9002, Abcam) and ChIP Kit Magnetic-One Step were used for ChIP assay according to the manufacturer’s instructions. A549 cells were cross-linked with formaldehyde, lysed in the SDS buffer and sheared mechanically by sonication to fragment the DNA. Protein-DNA complexes were precipitated with 2 μg normal rabbit IgG (#2729, CST), Histone H3 (D2B12) XP^®^ Rabbit mAb (#4620, CST), and NLE1 (1:50; #3742S, CST) antibodies, respectively. The complex was eluted from the antibodies and the eluted DNA fragment was assessed by qPCR (forward primer of CDK1: 5’-CCATACCCATTGACTAACTAT-3’, reverse primer of CDK1: 5’-ACCCCTTCCTCTTCACTTTC-3’).

### 2.13 Dual-luciferase assay

CDK1-WT and CDK1-Mut promoters were constructed into the GL002 vector and these plasmids were transfected into A549 cells with or without NLE1 or E2F1 overexpression. The luciferase activity was measured with a Promega Dual-Luciferases Reporter Assay kit according to the manufacturer’s protocols after transfection.

### 2.14 Animal experiment

20 female 4 weeks old specific pathogen free BALB/c nude mice were purchased from Beijing Vital River Experimental Animals Co., Ltd for animal experiment. 1 × 10^7^ infected A549 cells or control cells were subcutaneous injected into mice (two groups with 10 in each, randomly assigned) for *in vivo* tumorigenicity. Tumor size and weight were observed and measured (two times/week, V = π/6×L×W^2^, L = longest dimension and W = width). After 21 days, all mice were anaesthetized by 0.7% sodium pentobarbital (10 μL/g, SIGMA), and tumor burden was assessed by *in vivo* fluorescence expression under an IVIS Spectrum Imaging System (Perkin Elmer). Mice were humanely sacrificed and tumor tissues were removed and collected, 5 mm thick sections were stained for Ki-67 analysis. This study was approved by Xinhua Ethics committee affiliated to Shanghai Tongji University School of Medicine (Approval number: XHEC-NSFC-2021-242).

### 2.15 Statistics analysis

All cell experiments were repeated three times and data was expressed as means ± standard deviations (SD). Statistical analysis was performed using GraphPad Prism 8.0 and SPSS 21.0. Differences between groups were analyzed using Student’s t test or one-way ANOVA test. The statistical significance was determined using P<0.05. Sign test was used to analyze the NLE1 expression level in Lung cancer tissues and adjacent normal tissues. Mann-Whitney U was applied to analysis the relationship between NLE1 expression and tumor characteristics. Spearman’s test was used to analyze the correlation between the expression level of NLE1 in cancer tissues and Pathological staging. The relation between NLE1 gene expression and overall survival of lung cancer was analyzed by Kaplan-Meier survival analysis.

## 3 Results

### 3.1 NLE1 is elevated in non-small cell lung cancer tissues and cell lines

**Figure 1:**
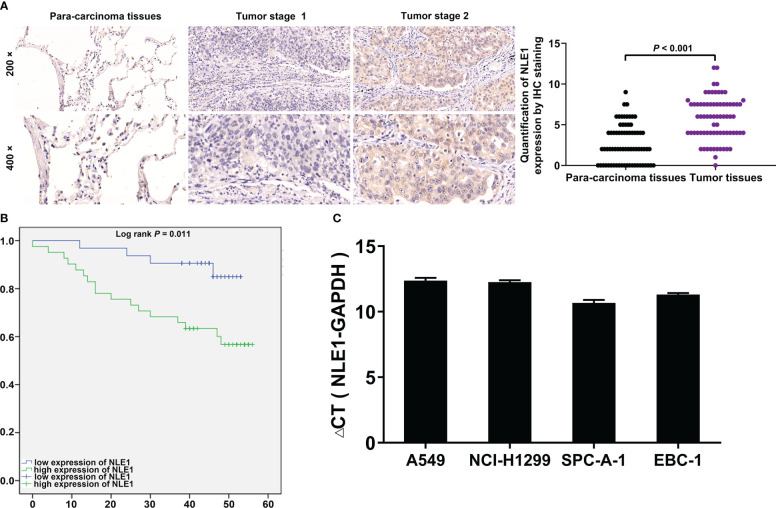
NLE1 was up-regulated in non-small cell lung cancer. **(A)** The expression levels of NLE1 in NSCLC tumor tissues and para-carcinoma tissues were determined by IHC staining. IHC scores of all tissue samples were used for statistical analysis. **(B)** Kaplan-Meier analysis showed that higher NLE1 expression was correlated with a shorter overall survival. **(C)** The expression levels of NLE1 in a panel of human NSCLC cell lines were determined by qRT-PCR. Results were presented as mean ± SD (n ≥ 3).

To clarify the underlying role of NLE1 in NSCLC development, we performed IHC analysis of a tissue microarray containing 73 NSCLC specimens and 71 non-tumor samples. As observed in **Figure 1A** and **Table 1**, high NLE1 expression was observed in 56.2% tumor specimens (41/73), while among 71 non-tumor samples, tissues harboring low NLE1 levels accounted for the majority (60/71) (*P* < 0.001). Besides, we investigated the clinical significance of NLE1 in NSCLC, and found that high NLE1 expression was associated with pathological stage (**Tables 2**, and **3**, *P* < 0.01). We further conducted Kaplan-Meier analysis, showing that higher NLE1 expression was correlated with a remarkable shorter overall survival (**Figure 1B**). In addition, abundant NLE1 level was also detected in a panel of NSCLC cell lines, namely A549, NCI-H1299, SPC-A-1 and EBC-1, especially in A549 and NCI-H1299 cells (**Figure 1C**). Taken together, there was a pattern of elevated NLE1 in NSCLC.

**Table 1:** Expression patterns of NLE1 in non-small cell lung cancer tissues and para-carcinoma tissues revealed in immunohistochemistry analysis.

NLE1 expression	Tumor tissue		Para-carcinoma tissue		*P* value
	Cases	Percentage	Cases	Percentage	
Low	32	43.8%	60	84.5%	< 0.001
High	41	56.2%	11	15.5%	

**Table 2:** Relationship between NLE1 expression and tumor characteristics in patients with non-small cell lung cancer.

Features	No. of patients	NLE1 expression		*P* value
		low	high	
All patients	73	32	41	
Age (years)				0.675
≤ 62	38	16	22	
> 62	34	16	18	
Gender				0.164
Male	67	31	36	
Female	6	1	5	
Tumor size				0.325
< 5cm	34	17	17	
≥ 5cm	39	15	24	
Lymph node positive				0.052
≤ 0	45	24	21	
> 0	27	8	19	
Grade				0.160
I	2	2	0	
II	53	24	29	
III	18	6	12	
Stage				0.003
1	27	17	10	
2	25	11	14	
3	21	4	17	
Tumor infiltrate				0.751
T1	5	1	4	
T2	51	24	27	
T3	16	6	10	
T4	1	1	0	
lymphatic metastasis (N)				0.117
N0	45	24	21	
N1	11	6	5	
N2	6	0	6	

**Table 3:** Relationship between NLE1 expression and tumor characteristics in patients with non-small cell lung cancer.

		NLE1
Stage	Spearman correlation	0.354
	Signification (double-tailed)	0.002
	N	73

### 3.2 NLE1 knockdown inhibits cancer malignant behaviors in non−small−cell lung cancer cells

**Figure 2:**
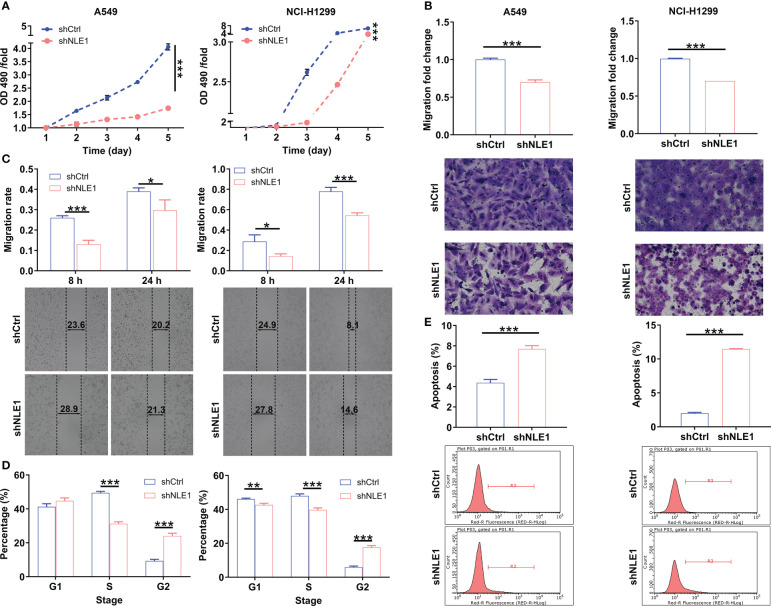
The effects of NLE1 knockdown on non-small cell lung cancer cell phenotypes. **(A)** The cell proliferation levels of A549 and NCI-H1299 cells were assessed after infecting lentiviruses by MTT assay. **(B, C)** The cell migration abilities of A549 and NCI-H1299 cells were evaluated after infecting lentiviruses by transwell assay **(B)** and wound-healing assay **(C)**. Magnification times: 200×. **(D)** The effects of NLE1 knockdown on cell cycle of A549 and NCI-H1299 cells were determined by flow cytometry. **(E)** The effects of NLE1 knockdown on cell apoptosis of A549 and NCI-H1299 cells were examined by flow cytometry. Results were presented as mean ± SD (n ≥ 3). * *P* < 0.05, ** *P* < 0.01, *** *P* < 0.001.

In this context, we aimed to explore the biological functions of NLE1 in NSCLC *in vitro*. The cell lines transfected with shRNA targeting NLE1, A549-shNLE1 and NCI-H1299-shNLE1, were developed to unveil the effects of NLE1 knockdown on the malignant behaviors of NSCLC cells (**Figure S1**). As visualized in **Figure 2A**, silencing NLE1 significantly suppressed cell proliferation levels (*P* < 0.001). To evaluate whether NLE1 knockdown could suppress cell migration, the transwell assay was performed. The results indicated that NLE1 knockdown significantly reduced the number of migrated cells (**Figure 2B**, *P* < 0.001). Additionally, as measured by the wound-healing assay, migration was clearly decreased in the shNLE1 groups, consistent with the aforementioned findings (**Figure 2C**). Moreover, in those NLE1-deficient cells, cell cycle was arrested in G2 phase (**Figure 2D**). Moreover, increased apoptosis percentage of cells was observed in the shNLE1 groups, which implied that silencing NLE1 in both cell lines significantly enhanced cell apoptosis (**Figure 2E**, *P* < 0.001). In cases of enhanced cell apoptosis, a human apoptosis antibodies array assay demonstrated that NLE1 exhaustion promoted cell apoptosis in a regulating apoptosis-related elements manner. Specifically, we detected elevated Caspase3, IGFBP-6 and p21, while reduced HSP27, HSP70, IGF-I, IGF-II, IGF-1sR, Livin, Survivin, sTNF-R1 and XIAP (**Figure S2**). Collectively, these data validated that NLE1 depletion relieved the malignant behaviors of NSCLC *in vitro*.

### 3.3 NLE1 regulates CDK1 level *via* E2F1 transcription

**Figure 3:**
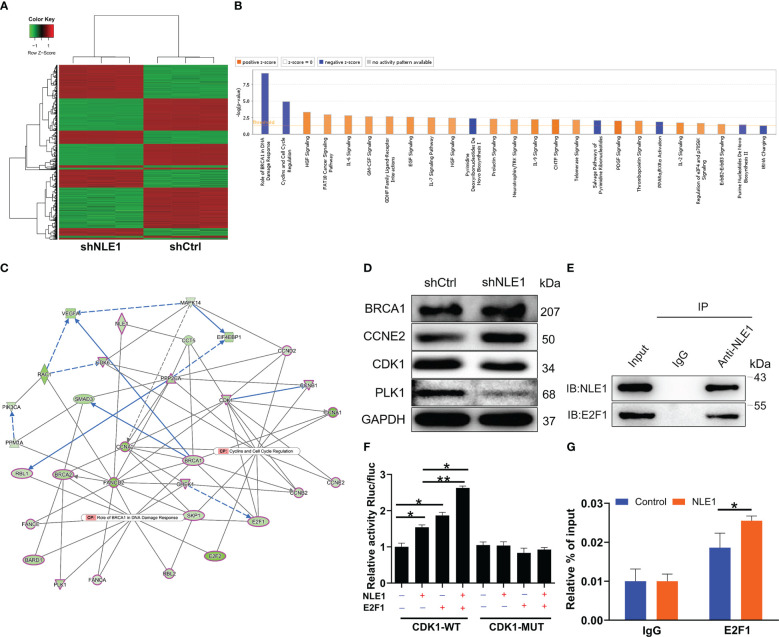
Exploration of underlying mechanism by Genechip and IPA analysis. **(A)** The heatmap of DEGs identified by RNA-sequencing of cells treated with shCtrl (n =3) or shNLE1 (n = 3). **(B)** Canonical signaling pathway based on IPA was performed to identify the pathways enrichment of DEGs. **(C)** Interaction network diagram between NLE1 with Role of BRCA1 in DNA Damage Response and Cyclins and Cell Cycle Regulation was analyzed by IPA. Ellipse Shape, transcription regulator; del operator, kinase; triangle, phosphatase; hexagon, translation regulator. The red outlines indicated the highlighted connectors in this network. **(D)** The expression of several significantly differentially expressed genes was identified by western blot in NCI-H1299 cells with shCtrl and shNLE1. **(E)** Co-IP assay was used to verify whether there was protein interaction between NLE1 and E2F1. **(F)** The dual-luciferase assay was performed to assess the interaction between E2F1 and CDK1 promotor. **(G)** ChIP-qPCR assay was conducted to confirm that NLE1 was responsible for the transcriptional regulation of CDK1 by E2F1. The data are expressed as mean ± SD (n ≥ 3). * *P* < 0.05, ** *P* < 0.01.

To further investigate the downstream factors in the regulation of NSCLC by NLE1, we performed a GeneChip PrimeView Human Gene Expression Array on shCtrl and shNLE1 infected NCI-H1299 cells. We found that 1251 genes were up-regulated and 1613 were down-regulated in shNLE1 group based on the threshold of an absolute fold change ≥ 1.5 and *FDR* < 0.05 (**Figure 3A**). Canonical signaling pathways according to Ingenuity pathway analysis (IPA) showed that NLE1 knockdown evidently impaired Role of BRCA1 in DNA Damage Response and Cyclins and Cell Cycle Regulation (**Figure 3B**). On that account, we focused on the interaction network between NLE1 with these both pathways, suggesting that NLE1 could affect the expression of BARD1, BRCA1, BRCA2, CCNA1, CCNA2, CCNB1, CCNB2, CCND2, CCNE2, CDK1, CDK6, CHEK1, E2F1, E2F2, FANCA, FANCE, PLK1, PPP2CA, RBL1, RBL2 and SKP1 (**Figure 3C**). Next, we selected several candidates for western blot verification in shNLE1 NCI-H1299 cells. Indeed, the results demonstrated that CDK1 and PLK1 were down-regulated (**Figure 3D**). As we know, CDK1 is involved in cell cycle regulation (21). More importantly, CDK1 is a well-known cancer-promoting factor in NSCLC (22), implying CDK1 as a potential downstream of NLE1. Otherwise, we utilized the TRRUST website (https://www.grnpedia.org/trrust/), and found that E2F1 was an upstream transcription factor of CDK1. Subsequently, we predicted the binding site of E2F1 on CDK1 promotor through JASPER website (http://jaspar.genereg.net/analysis), and found such a site (5’- TTTCGCGC-3’) between +1873 and +1880. Additional Co-IP assay clarified the protein interaction between NLE1 and E2F1 (**Figure 3E**). A hypothesis arisen from all above results is that NLE1 may regulate the expression of CDK1 through E2F1-mediated transcription. We therefore performed a dual-luciferase assay using luciferase reporter constructs with wild-type (WT) and mutated CDK1 (MUT). The results showed that elevating NLE1 exclusively increased the luciferase activity driven by the CDK1 promoter in the WT group (*P* < 0.05) but not in the MUT group. More importantly, the augment of NLE1 promoted the binding of E2F1 to the CDK1 promoter (**Figure 3F**). Further ChIP-qPCR assay confirmed that NLE1 overexpression could enhance the transcriptional regulation of CDK1 by E2F1 (*P* < 0.05, **Figure 3G**). Our findings collectively illustrated that NLE1 could transcriptionally activate CDK1 by interacting with E2F1.

### 3.4 NLE1 regulates non−small−cell lung cancer progression *via* the Akt signaling pathway

**Figure 4:**
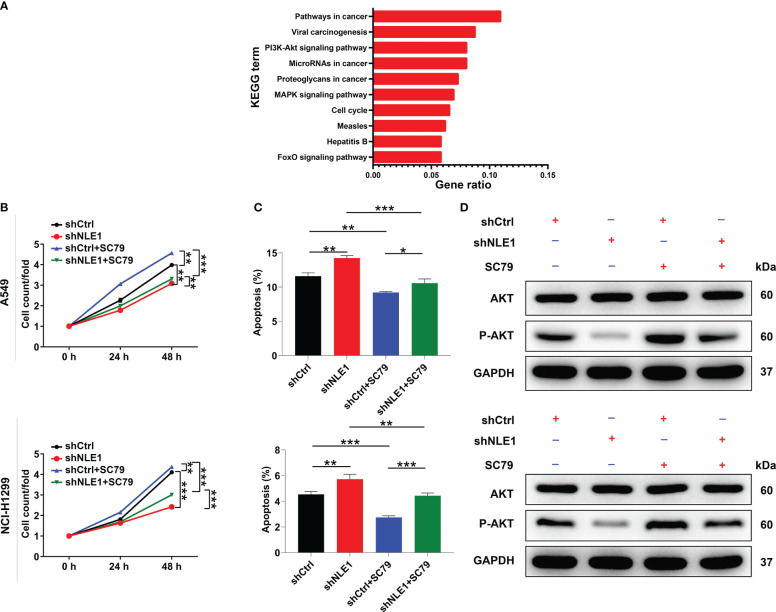
NLE1 regulated non−small−cell lung cancer *via* the Akt signaling pathway. **(A)** KEGG pathway analysis was performed using genes whose expression was increased or decreased by NLE1 silencing in PrimeView human gene expression array. **(B, C)** The cell proliferation levels **(B)** and apoptosis levels **(C)** of shCtrl and shNLE1 infected A549 and NCI-H1299 cells were tested after treating with 10 μM SC79. **(D)** The expression of AKT and p-AKT in shCtrl and shNLE1 A549 and NCI-H1299 cells was detected by western blot after treating with 10 μM SC79. The data are expressed as mean ± SD (n ≥ 3). ** *P* < 0.01, *** *P* < 0.001, *P < 0.05.

On the other hand, we conducted KEGG pathway analysis using genes whose expression was increased or decreased upon depleting NLE1 in PrimeView human gene expression array. The PI3K-Akt signaling pathway was enriched in downregulated genes in response to NLE1 depletion (**Figure 4A**), implying that PI3K-Akt signaling pathway is a possible target by which NLE1 promotes NSCLC cell proliferation and migration. To confirm this, we treated shCtrl and shNLE1 infected NCI-H1299 cells with 10 μM SC79, an AKT agonist, to measure the changes of cell phenotypes in response to SC79 treatment, indicating that cell proliferation levels in the shNLE1+10μM SC79 groups increased in comparison with shNLE1 group, while the cell apoptosis levels were decreased accordingly (**Figures 4B, C**). Moreover, western blot analysis suggested that the levels of p-Akt were attenuated in NLE1-deficient cells, which were raised upon treating with SC79 (**Figure 4D**). Taken together, these findings supported that the Akt pathway participated in the attenuation of cell proliferation and migration induced by NLE1 knockdown.

### 3.5 Augmenting CDK1 partially reverses the inhibitory effects of NLE1 knockdown on non−small−cell lung cancer

**Figure 5:**
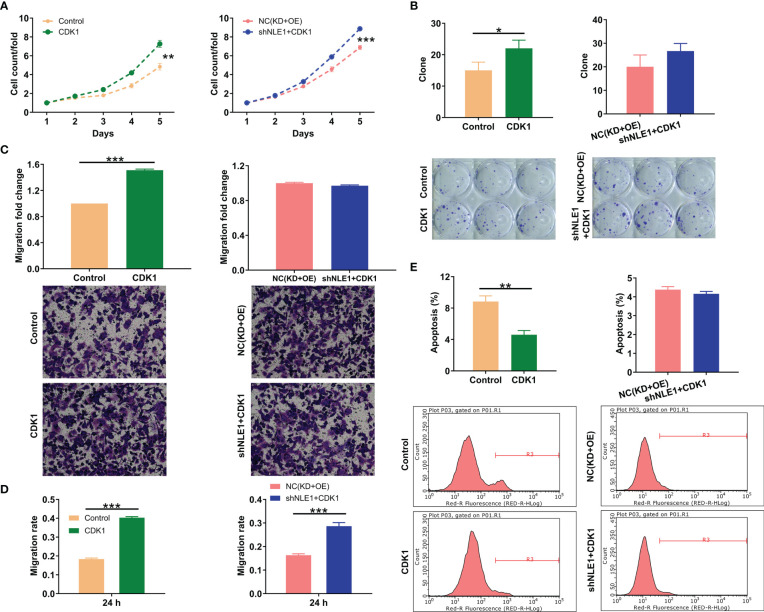
CDK1 elevation partially reversed the inhibitory effects of NLE1 knockdown on non−small−cell lung cancer. **(A)** Celigo cell counting assay was employed to show the effects of alone overexpressing CDK1 as well as simultaneously downregulating NLE1 and overexpressing CDK1 on the cell proliferation of NCI-H1299 cells. **(B)** Colony formation assay was used to evaluate the abilities of NCI-H1299 cells to form colonies in CDK1 and CDK1+shNLE1 groups. **(C, D)** The migration abilities of NCI-H1299 cells were detected in CDK1 and CDK1+shNLE1 groups by transwell assay **(C)** and wound-healing assay **(D)**. Magnification times: 200×. **(E)** The cell apoptosis of NCI-H1299 cells was examined by flow cytometry after infecting CDK1 and CDK1+shNLE1. Results were presented as mean ± SD (n ≥ 3). ** *P* < 0.01, *** *P* < 0.001, *P < 0.05.

Finally, we attempted to show whether NLE1 could influence NSCLC development by regulating CDK1. We thus smoothly constructed alone overexpressing CDK1 and simultaneously downregulating NLE1 and overexpressing CDK1 NCI-H1299 cell models to verify CDK1’s necessity in NLE1-induced regulation NSCLC progression (**Figure S3**). Cells transfected with Control lentivirus (the negative control for CDK1 overexpression) or both Control and shCtrl lentiviruses were used as negative control groups. The findings demonstrated that alone elevated CDK1 improved the capacities of cell proliferation, colony formation and cell migration, as well as suppressed cell apoptosis. More notably, overexpressing CDK1 could partially abolish the inhibitory effects of NLE1 knockdown on NSCLC, such as ameliorating proliferation and colony formation, mitigating apoptosis, and accelerating migration (**Figures 5A–E**). These data suggested that CDK1 harbored similar effects on the development of NSCLC as NLE1, and NLE1 played a role in promoting malignant progression of NSCLC through CDK1.

### 3.6 NLE1 depletion inhibits xenograft tumors in non−small−cell lung cancer

**Figure 6:**
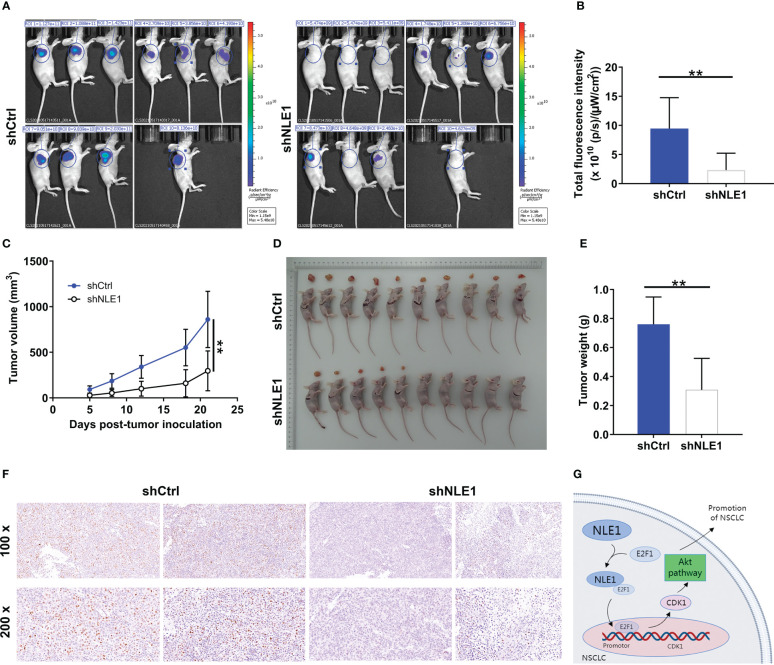
NLE1 depletion inhibits xenograft tumors in non−small−cell lung cancer. **(A)** A nude mice model was constructed through subcutaneously injecting NLE1-deficient A549 cells. **(B, C)** Following cell implantation, the fluorescence intensity **(B)** and tumor volume **(C)** were evaluated at indicated time. **(D)** The photos of tumors removed from mice models were collected. **(E)** The weight of xenografted tumors was measured. **(F)** The pattern of Ki-67 was detected *via* IHC staining in tumor sections from the mice models. **(G)** A diagram depicting the mechanism by which NLE1 regulates the development of non−small−cell lung cancer. The data were expressed as mean ± SD (n ≥ 3). ** *P* < 0.01.

Having shown that NLE1 depletion displayed potent suppressive effects *in vitro*, we further assessed the influence of NLE1 on tumor growth *in vivo*. We thus subcutaneously injected NLE1-deficient A549 cells into four-week-old BALB-c nude mice, and measured tumor growth indicators (GFP label on lentivirus vector) before sacrifice of animal (**Figure 6A**). Strikingly, the depletion of NLE1 contributed to significant diminishment of fluorescence intensity (*P* < 0.01, **Figure 6B**) as well as inhibition of tumor growth. Additionally, we also observed that the volume of NLE1-deficient tumors was smaller than that of the control group (**Figure 6C**). 21 days following cell implantation, mice were sacrificed to harvest tumors for weighing. Obviously, NLE1 inhibition was capable of decreasing tumor weight (*P* < 0.05, **Figures 6D, E**). Subsequently, we collected tumor tissues for Ki-67 detection. As expected, Ki-67 pattern was mitigated in tissues from NLE1-deficient group (**Figure 6F**). Together, these data illustrated that the inhibition of NLE1 obviously arrested the growth of xenografted tumors. Overall, the mechanism by which NLE1 regulates non−small−cell lung cancer development was shown in **Figure 6G**.

## 4 Discussion

Although the functions of Notch signaling pathway in the development of cancer are thought to be double-edged, accumulating evidence showed that activation of Notch signaling promote tumor progression through regulating proliferation, apoptosis, migration, invasion of cancer cells and self-renewal of cancer stem cells. As a key receptor in Notch signaling, Notch1 has been comprehensively studied and revealed to possess tumor-promoting functions. For example, based on the conjoint analysis of several cancer-related database, Hu et al. found that high expression levels of Notch receptors including Notch1/2/3/4 were significantly correlated with gastric cancer patients’ survival and higher infiltration levels of immune cells (23). Gan et al. provides more direct evidence of the tumor-regulating property of Notch1, which plays an important role in cell proliferation, apoptosis and invasion of tongue cancer cells (24). The research of Rice et al. showed that suppressing the activation of Notch signaling by inhibiting Notch1 could not only hinder the growth and metastasis of prostate cancer, but also enhance the therapeutic efficiencies of anti-androgen therapies (25). Recently, it was demonstrated that Notch1 also mediated the Nrf2-induced regulation of breast cancer (26). Moreover, the significance of inhibiting Notch1 signaling in preventing lung cancer development has also been clarified and indicated Notch1 as a promising therapeutic target and biomarker of lung cancer (27, 28). The *NLE1* codes for a member of the WD-repeat containing protein family, which participates in a variety of cell functions such as cytoskeleton assembly, cell division, transcriptional regulation, RNA processing and signal transduction. More importantly, NLE1 has been well-identified in Drosophila to possess direct regulatory effects on Notch1 activity (17, 18, 29). Therefore, it was fairly reasonable to deduce that NLE1 may also play some regulatory roles in the development of human cancer. In fact, although few, the role of NLE1 in the development and progression of tumor development has been studied to some extent. For example, Zhang et al. analyzed the gene expression profiling data of TCGA-COAD database and identified NLE1 as one of the key promotors for tumor growth of colon cancer (19). Moreover, Xiao et al. carried out a more detailed study and demonstrated that NLE1 could promote the development of malignant melanoma through promoting cell proliferation, cell migration and inhibiting cell apoptosis, indicating the role of NLE1 in melanoma development.

Herein, through detecting NLE1 expression in lung cancer tissues and para-carcinoma tissues, upregulation of NLE1 in lung cancer was revealed. Further statistical analysis showed that elevation of NLE1 expression was positively correlated with pathological stage of tumor and negatively correlated with survival of patients, indicating the potential tumor-promoting role of NLE1 in lung cancer. The conclusion deduced from clinical analysis was also proved by *in vitro* loss-of-function and gain-of-function studies, which illustrated the NLE1-induced change of cell phenotypes. Actually, current results showed that NLE1 presented similar functions with Notch1 in the regulation of human cancer. However, the relationship between NLE1 and Notch1 was still not clear. Whether NLE1 execute its function through affecting the activation of Notch1 signaling requires further investigation. Despite of these, the mechanistic study in our study identified CDK1 as a potential downstream target of NLE1 in the regulation of lung cancer.

Cyclin dependent kinases (CDKs) are a class of multifunctional enzymes that regulate the cell cycle by binding with cyclins, and are regarded as potential diagnostic and therapeutic targets for cell proliferation disorder related diseases including malignant tumors (30). Abnormal expression of CDKs can lead to abnormal cell proliferation and dysfunction of related genes and proteins (31, 32). CDK1/Cyclin B complex is the main regulator of G2/M phase transition, which regulates cell cycle transition and mitosis in late G2 phase by activating nuclear membrane, spindle and actin cytoskeleton reorganization through basement membrane phosphorylation. A large number of studies have shown that CDK1 is closely related to the occurrence and development of malignant tumors (33). For example, Qian et al. reported that KIAA1429 may act as a “writer” for m6A methylation of CDK1 thus regulating its expression and further the development of breast cancer (34). Moreover, CDK1 itself also was involved in multiple cancer development through inducing the phosphorylation of hTERT and the hTERT-mediated RNA dependent RNA polymerase (RdRP) activity (35). Besides, numerous studies have demonstrated the critical role of CDK1 in the development and drug resistance in lung cancer, which could act as prognostic biomarker and therapeutic target (22, 36–38). In this study, except for the regulatory effects of CDK1 itself, we also found that knockdown of CDK1 could partially eliminate the promotion effects of lung cancer induced by NLE1 overexpression, suggesting CDK1 as an essential link in the function of NLE1 in lung cancer. As for the mechanism by which NLE1 regulates CDK1, it was discovered that NLE1 knockdown could significantly affect the E2F1-mediated transcription of CDK1 through physically interacting E2F1, which in turn affects the expression of CDK1. Actually, the E2F1-mediated transcription of downstream targets was also reported to be influenced by a physical interaction with PTEN (39). On the other hand, Akt has been demonstrated to be a newly reported substrate of CDK1, whose phosphorylation was regulated by it (40, 41). Correspondingly, we also showed the involvement of Akt pathway in NLE1-induced regulation of lung cancer cell phenotypes.

## 5 Conclusions

In conclusion, this study identified NLE1 as a critical regulator of lung cancer, which is upregulated in lung cancer and plays a vital role in the regulation of cell phenotypes. Moreover, mechanistic study clarifies that the regulation of lung cancer by NLE1 needs the participation of CDK1 as a downstream target, whose E2F1-mediated transcription was regulated by NLE1. Therefore, NLE1 may act as a prognostic indicator and therapeutic target of lung cancer. In our future work, whether NLE1 regulates lung cancer through Notch1 signaling and the molecular regulatory mechanism between NLE1 and CDK1 would be further explored and investigated.

## Data availability statement

The original contributions presented in the study are included in the article/**Supplementary Material**. Further inquiries can be directed to the corresponding authors.

## Ethics statement

This study was approved by Ethics Committee of Xinhua Hosptial affiliated to Shanghai Jiaotong University School of Medicine. The patients/participants provided their written informed consent to participate in this study.

## Author contributions

HX designed this program. PX, LW, and BM operated the cell and animal experiments. LJ and XX conducted the data collection and analysis. RH and FH produced the manuscript which was checked by FD. All the authors have confirmed the submission of this manuscript.
