# Supplementary material for: Identification of NLE1/CDK1 axis as key regulator in the development and progression of non-small cell lung cancer
Source: Front Oncol. 2023 Feb 1;12:985827. doi: 10.3389/fonc.2022.985827 (PMC9931185; doi:10.3389/fonc.2022.985827)
Supplement: Supplementary file 1 [file DataSheet_1.zip › Original Data 1/Figure 2E/A549/shCtrl-1.pdf]

Well Number: F01 Sample ID: F01
File Name: E:/张静/20190409 A549 DW/2019-04-09_at_05-05-30pm.fcs

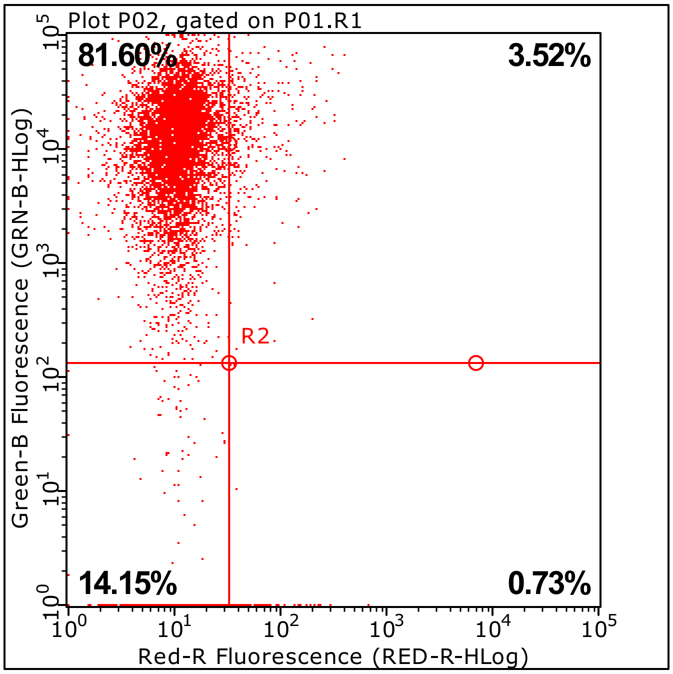

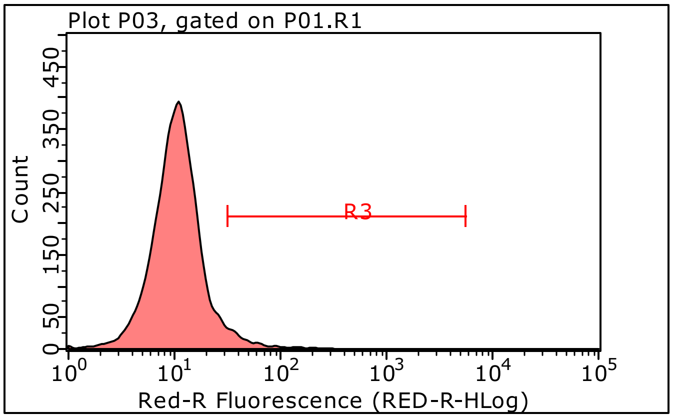

Well	Sample ID	Date	R2.Percent.UL Percent for R2 gated by P01.R1 (%)	R2.Percent.UR Percent for R2 gated by P01.R1 (%)	R2.Percent.LL Percent for R2 gated by P01.R1 (%)	R2.Percent.LR Percent for R2 gated by P01.R1 (%)
F01	F01	04.11.2019	81.60	3.52	14.15	0.73

Well	R3.Percent Percent for R3 gated by P01.R1 (%)
F01	4.25
