# Supplementary material for: Identification of NLE1/CDK1 axis as key regulator in the development and progression of non-small cell lung cancer
Source: Front Oncol. 2023 Feb 1;12:985827. doi: 10.3389/fonc.2022.985827 (PMC9931185; doi:10.3389/fonc.2022.985827)
Supplement: Supplementary file 1 [file DataSheet_1.zip › Original Data 1/Figure 2E/NCI-H1299/shCtrl-3.pdf]

Well Number: C04 Sample ID: C04
File Name: E:/张静/20190313/20190306 H1299 DW/2019-03-06_at_04-24-58pm.fcs

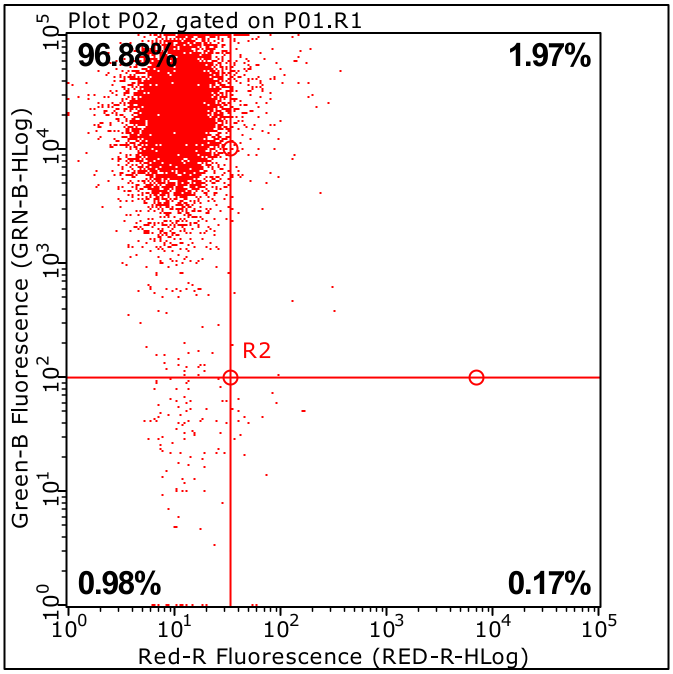

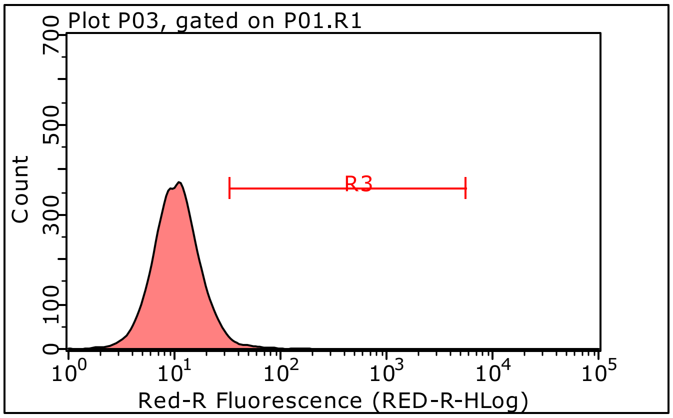

Well	Sample ID	Date	R2.Percent.UL Percent for R2 gated by P01.R1 (%)	R2.Percent.UR Percent for R2 gated by P01.R1 (%)	R2.Percent.LL Percent for R2 gated by P01.R1 (%)	R2.Percent.LR Percent for R2 gated by P01.R1 (%)
C04	C04	03.24.2019	96.88	1.97	0.98	0.17

Well	R3.Percent Percent for R3 gated by P01.R1 (%)
C04	2.14
