# Supplementary figures and images for: Identification of NLE1/CDK1 axis as key regulator in the development and progression of non-small cell lung cancer
Source: Front Oncol. 2023 Feb 1;12:985827. doi: 10.3389/fonc.2022.985827 (PMC9931185; doi:10.3389/fonc.2022.985827)

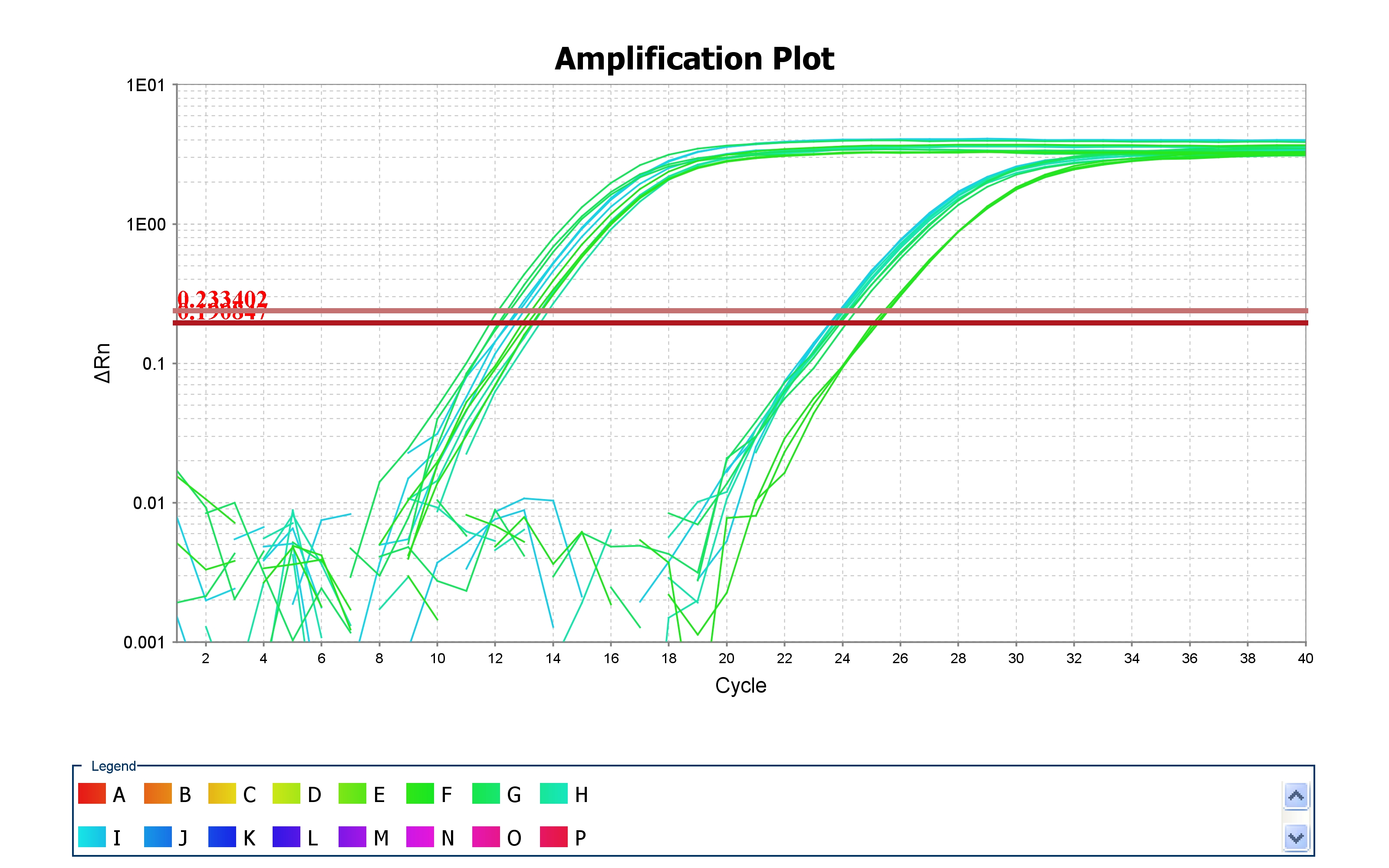

Supplement: Supplementary file 1 [file DataSheet_1.zip › Original Data 1/Figure 1C/Amplification Plot.jpg]

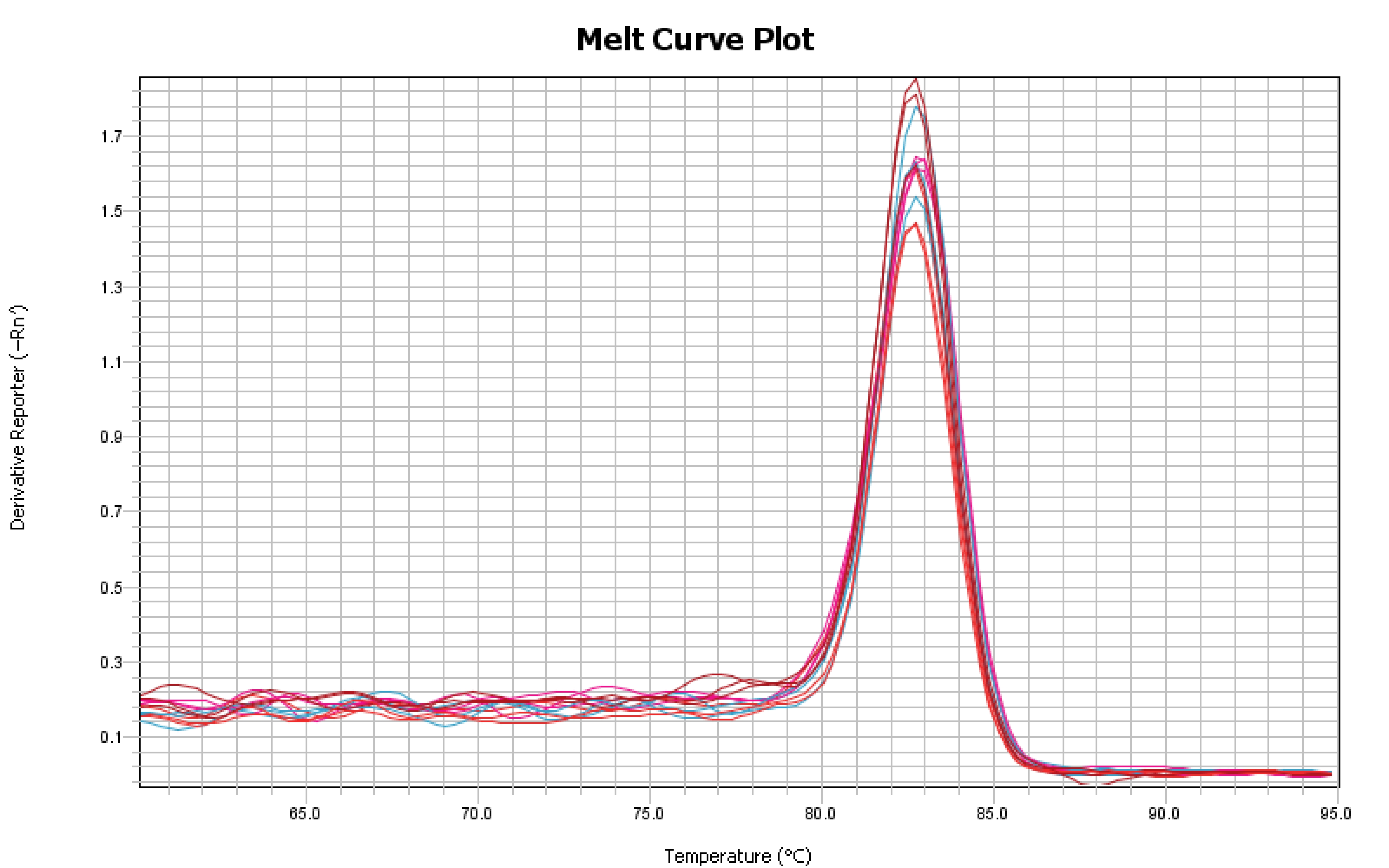

Supplement: Supplementary file 1 [file DataSheet_1.zip › Original Data 1/Figure 1C/Melt Curve Plot H-GAPDH.jpg]

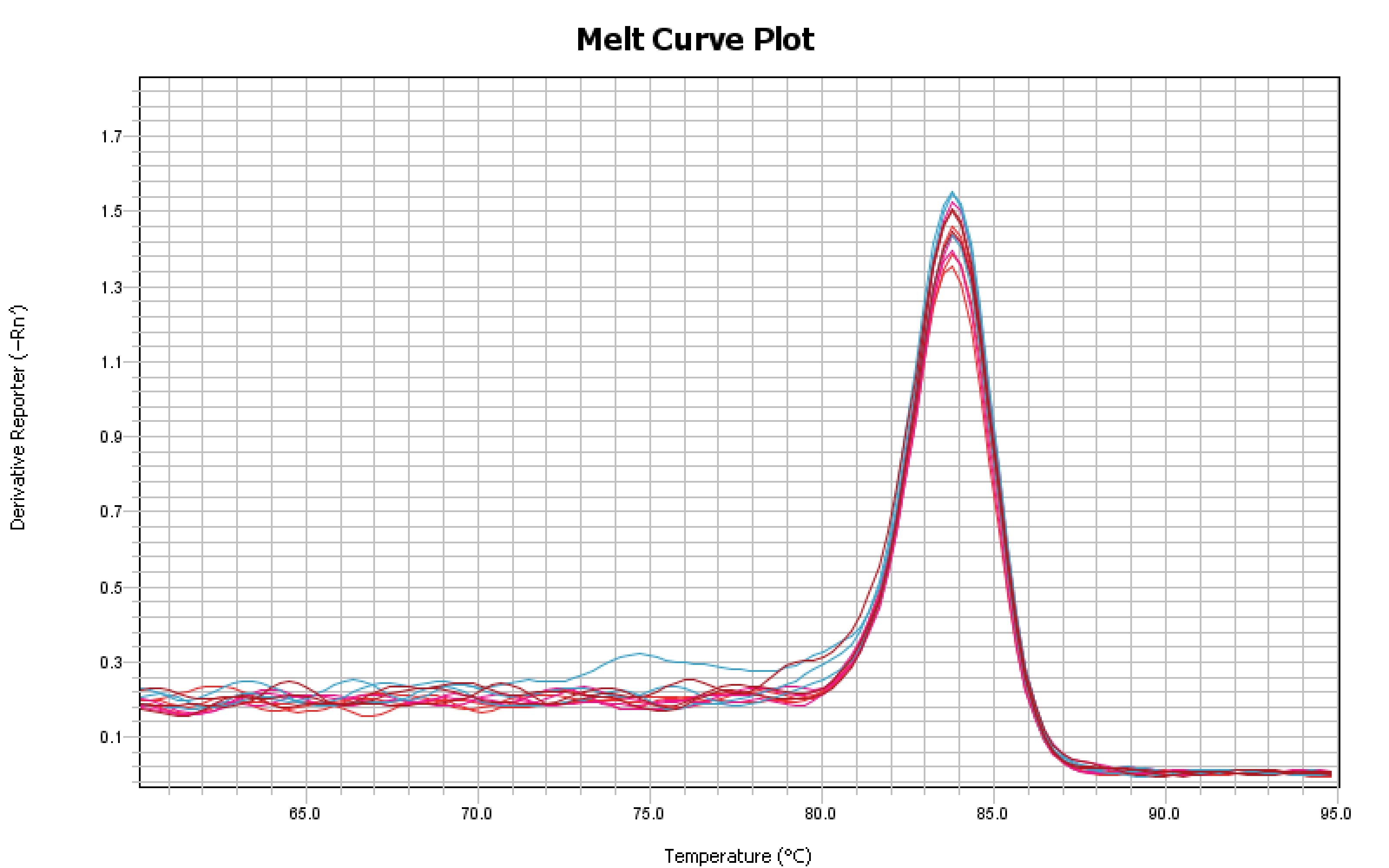

Supplement: Supplementary file 1 [file DataSheet_1.zip › Original Data 1/Figure 1C/Melt Curve Plot H-NLE1.jpg]

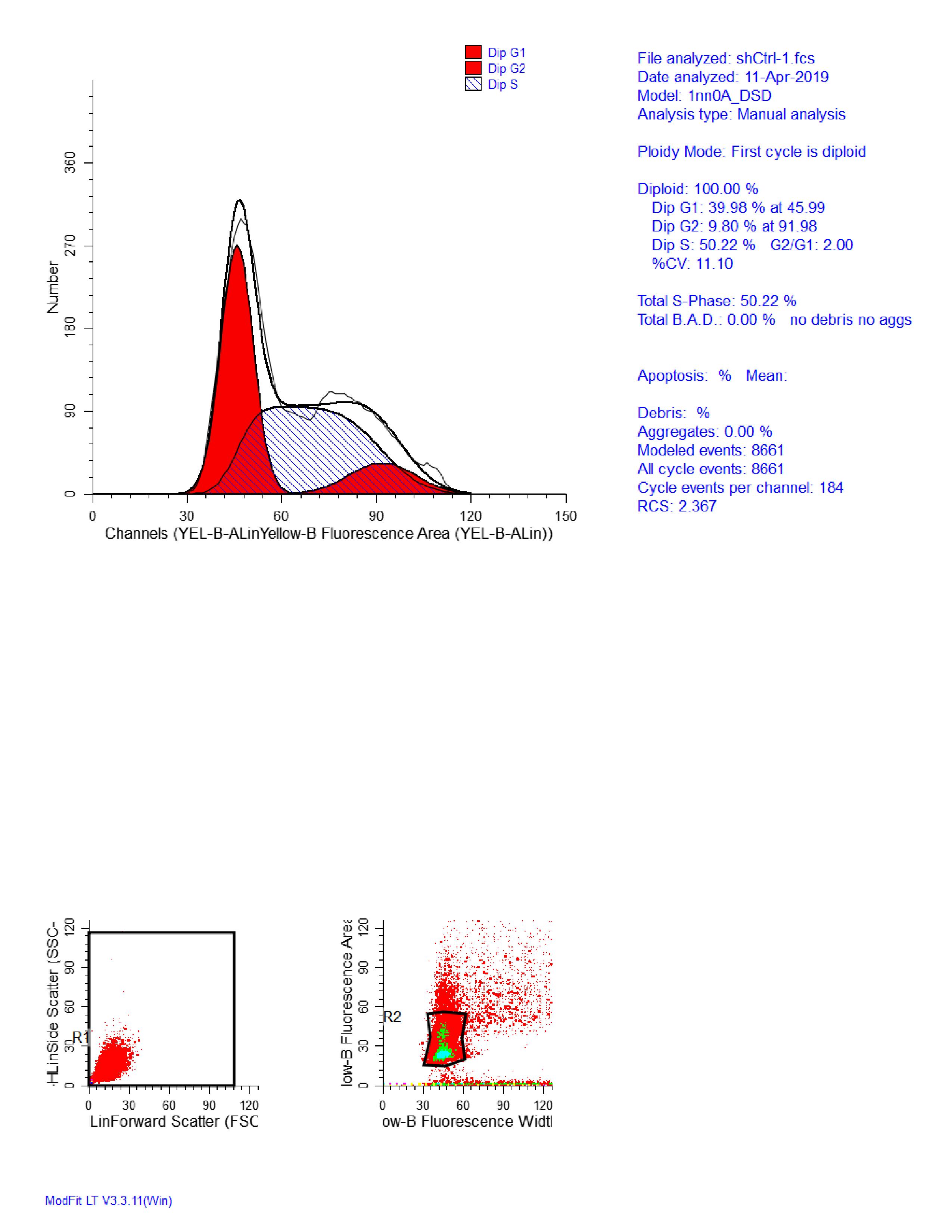

Supplement: Supplementary file 1 [file DataSheet_1.zip › Original Data 1/Figure 2D/A549/shCtrl-1.jpg]

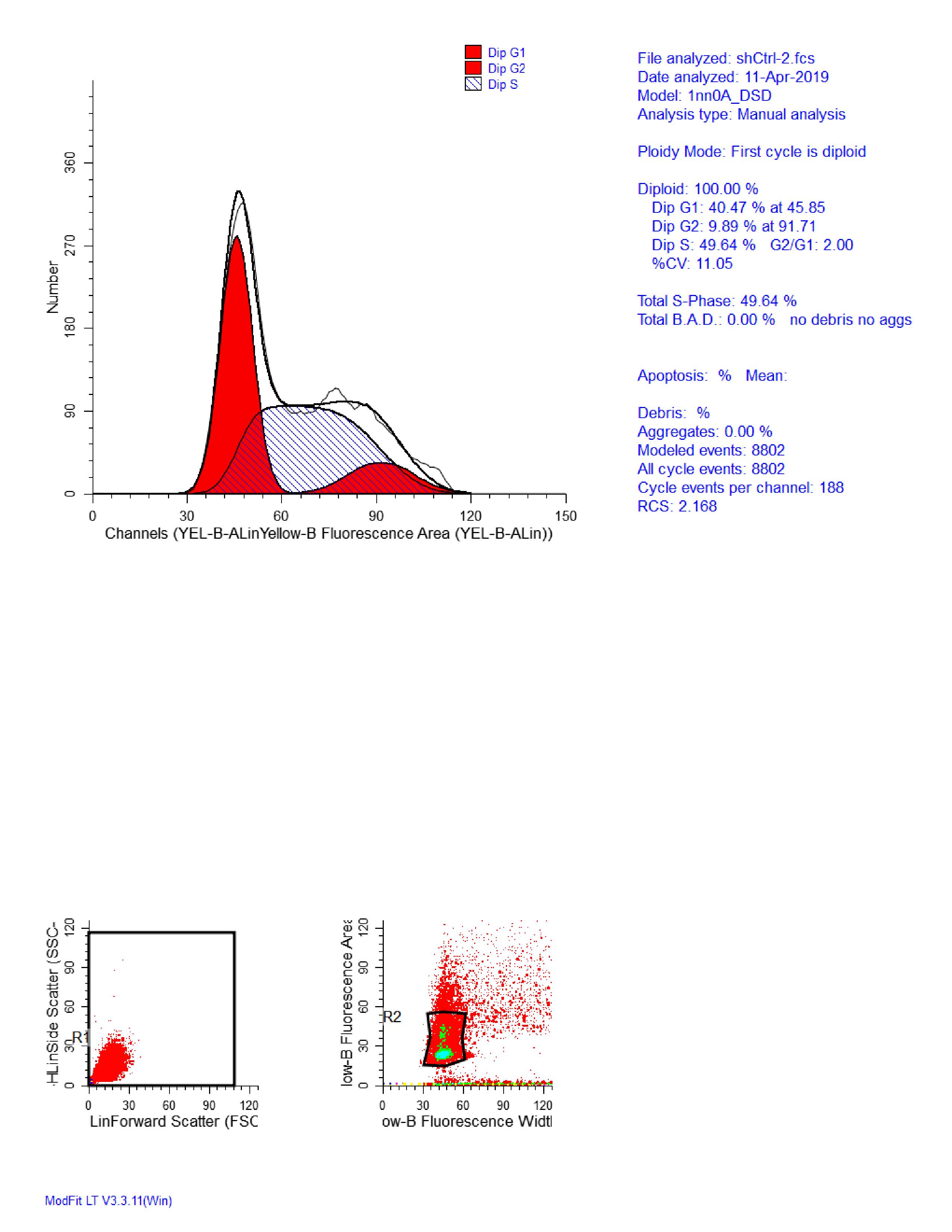

Supplement: Supplementary file 1 [file DataSheet_1.zip › Original Data 1/Figure 2D/A549/shCtrl-2.jpg]

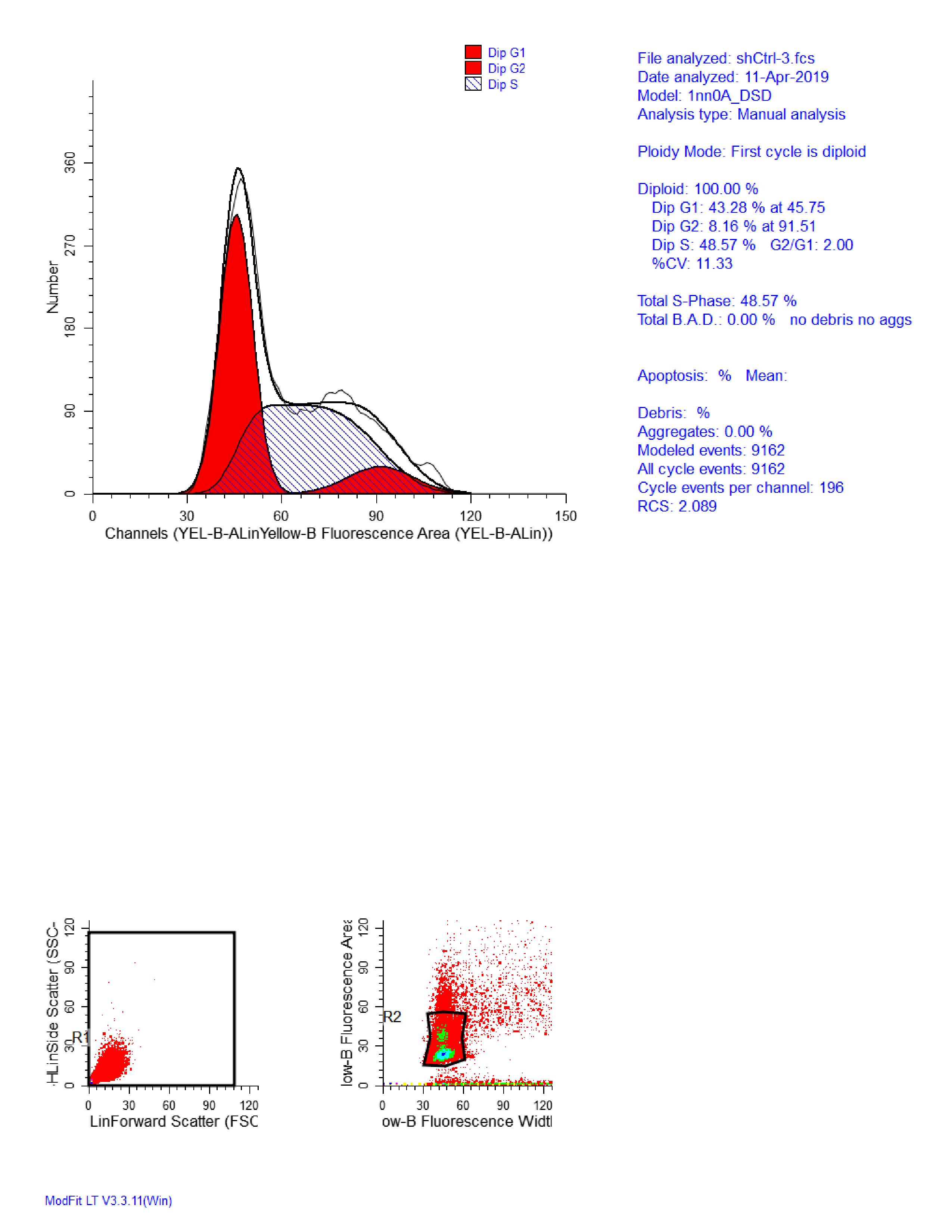

Supplement: Supplementary file 1 [file DataSheet_1.zip › Original Data 1/Figure 2D/A549/shCtrl-3.jpg]

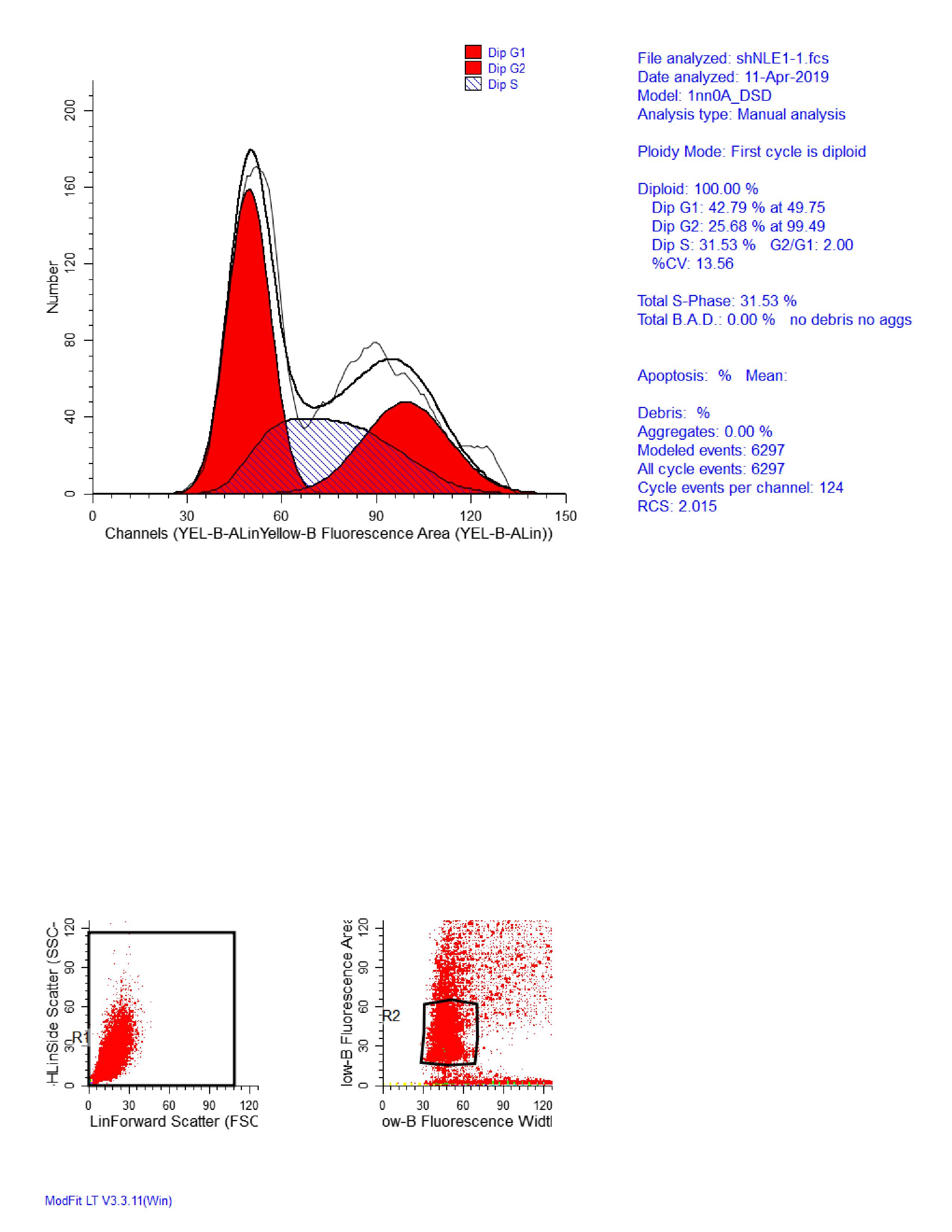

Supplement: Supplementary file 1 [file DataSheet_1.zip › Original Data 1/Figure 2D/A549/shNLE1-1.jpg]

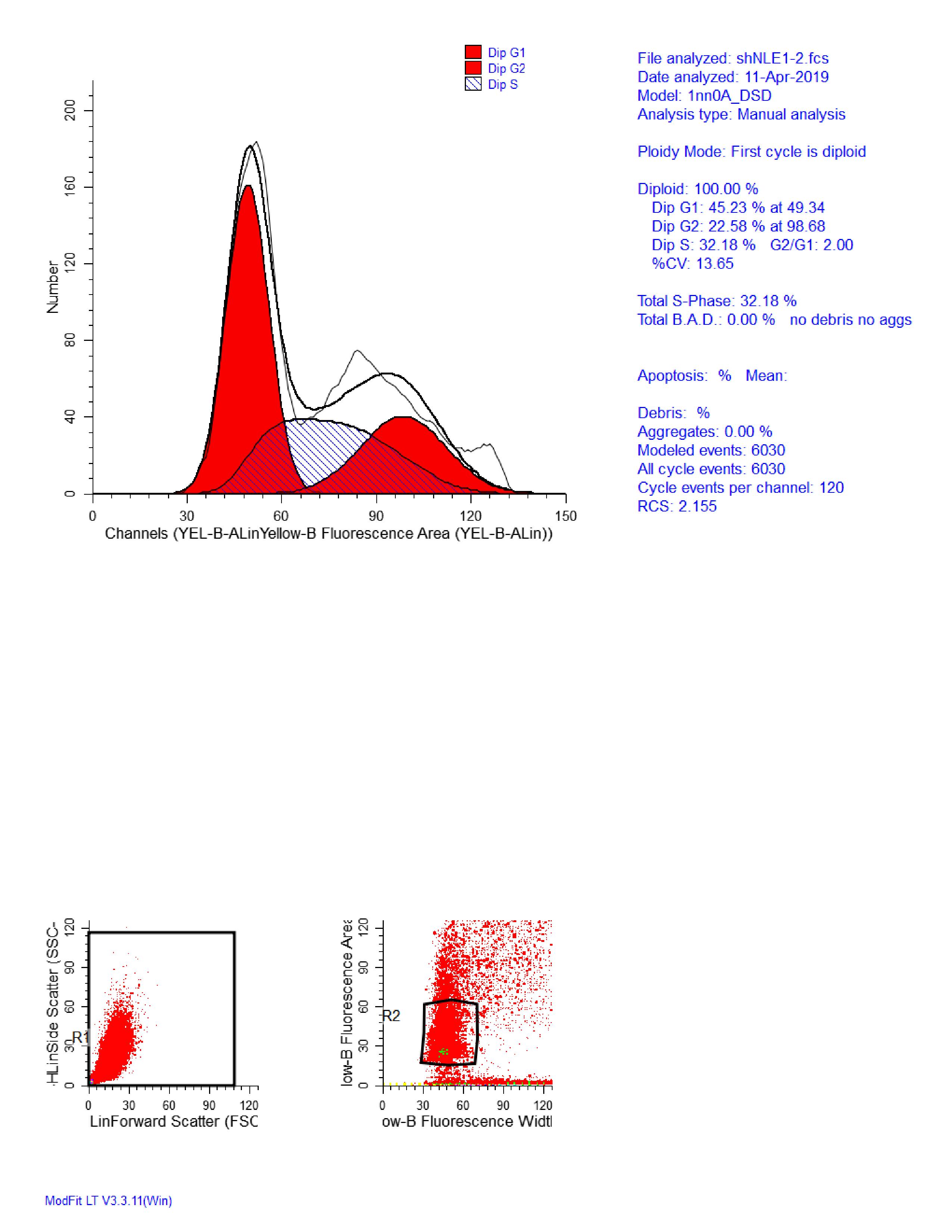

Supplement: Supplementary file 1 [file DataSheet_1.zip › Original Data 1/Figure 2D/A549/shNLE1-2.jpg]

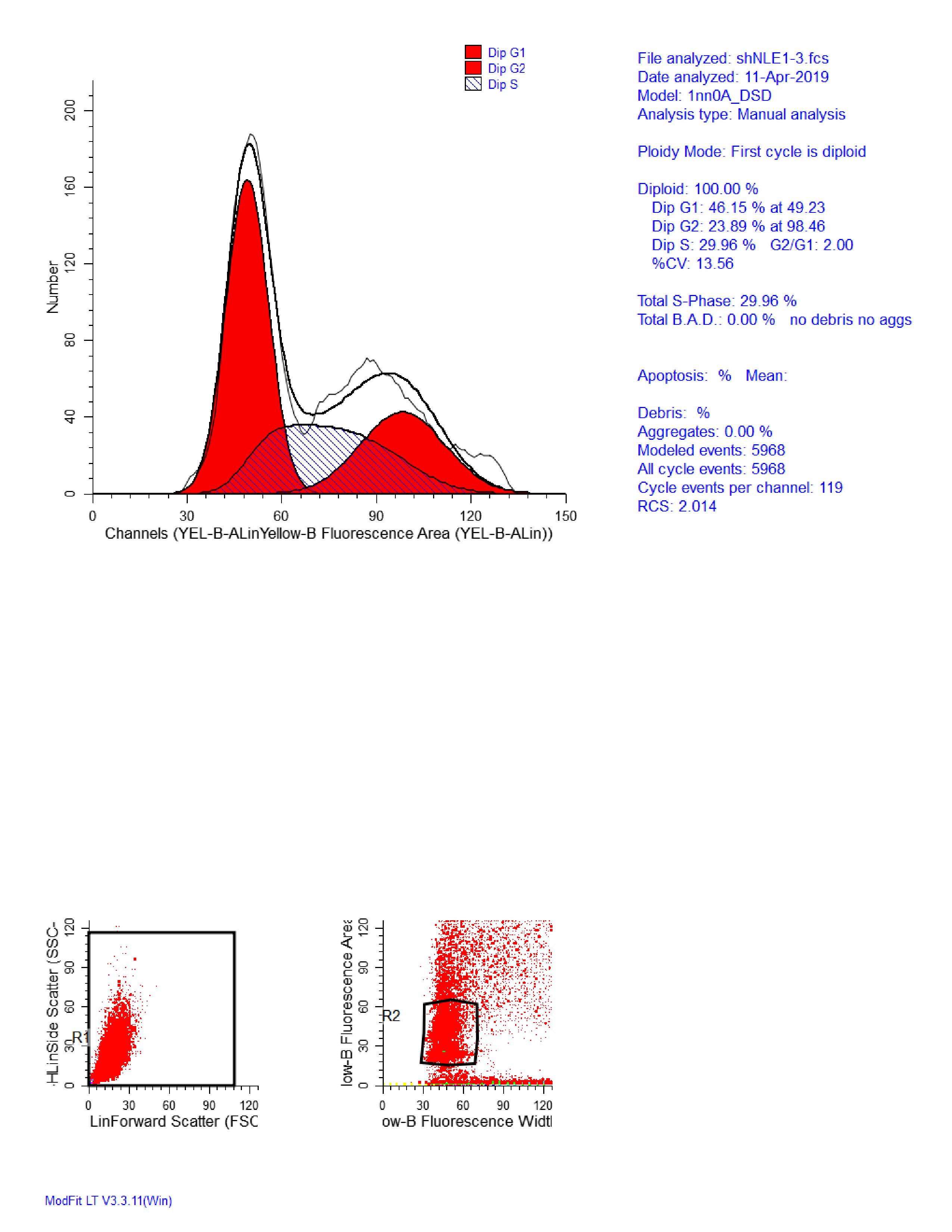

Supplement: Supplementary file 1 [file DataSheet_1.zip › Original Data 1/Figure 2D/A549/shNLE1-3.jpg]

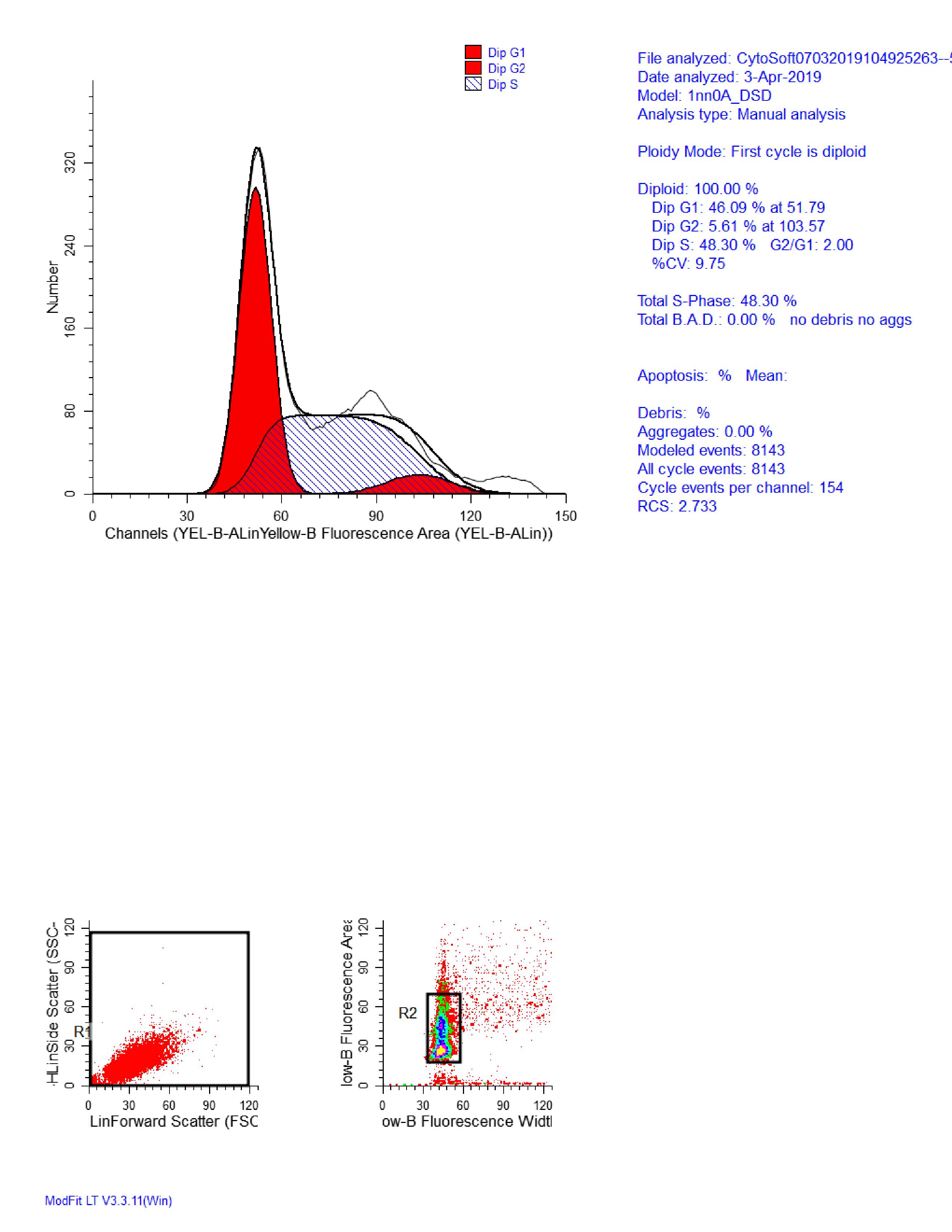

Supplement: Supplementary file 1 [file DataSheet_1.zip › Original Data 1/Figure 2D/NCI-H1299/shCtrl-1.jpg]

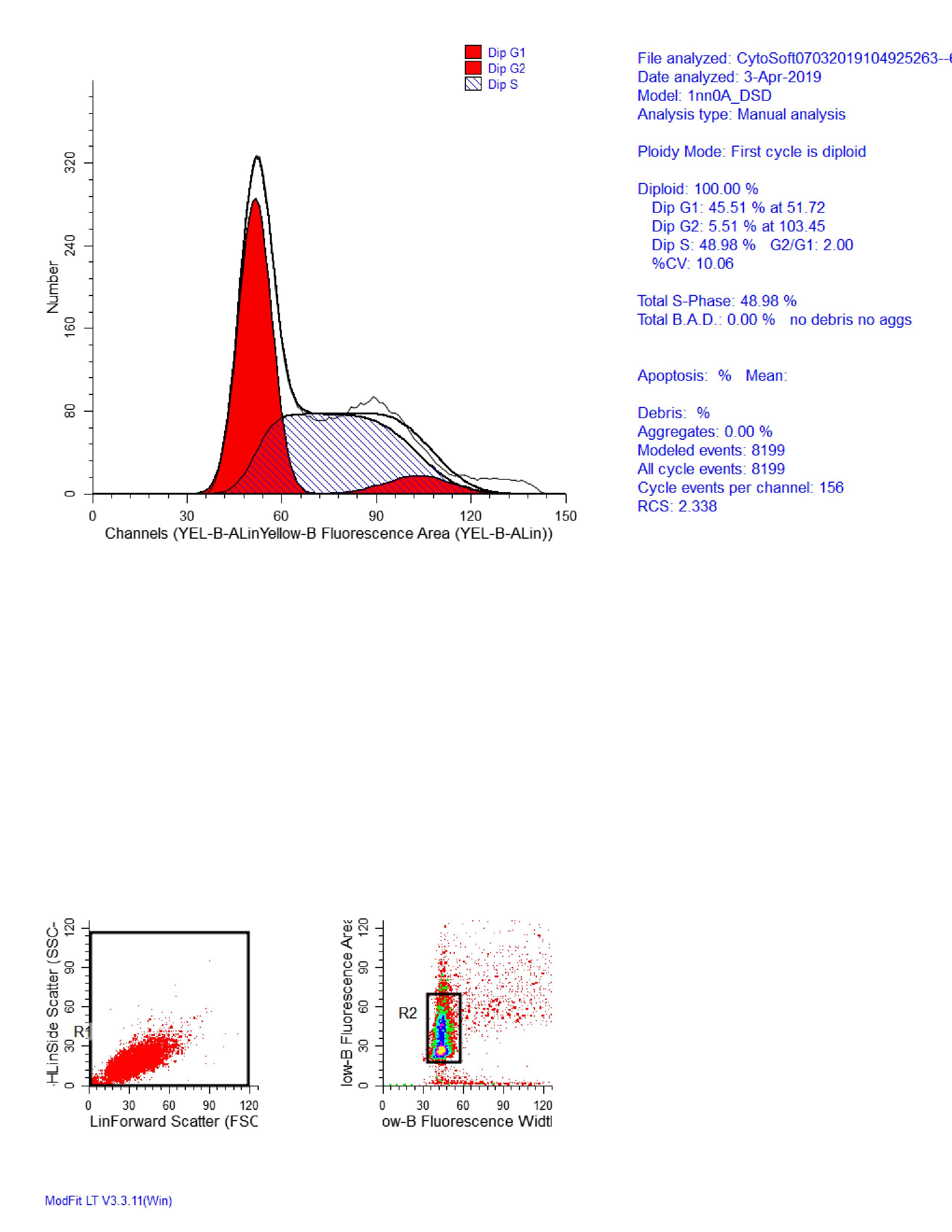

Supplement: Supplementary file 1 [file DataSheet_1.zip › Original Data 1/Figure 2D/NCI-H1299/shCtrl-2.jpg]

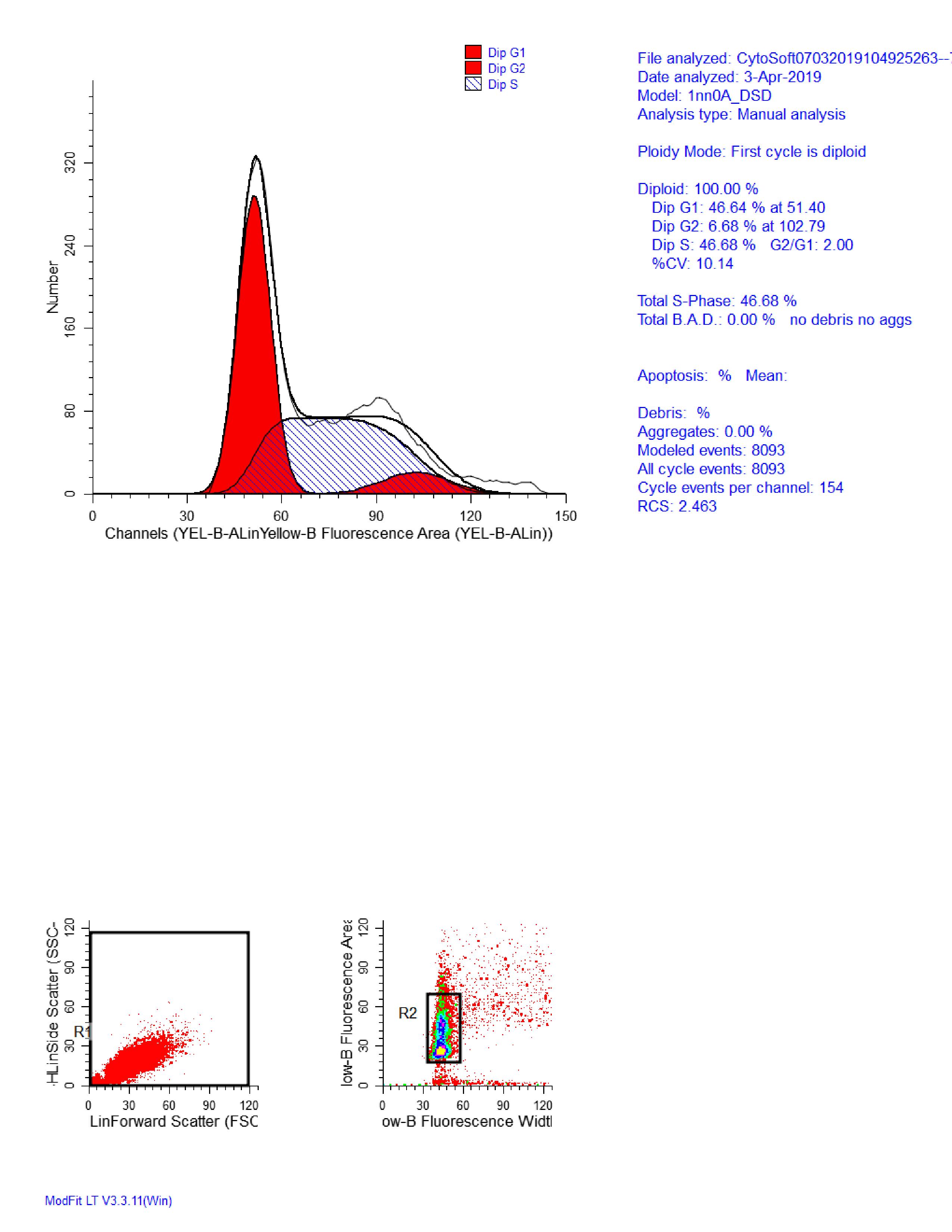

Supplement: Supplementary file 1 [file DataSheet_1.zip › Original Data 1/Figure 2D/NCI-H1299/shCtrl-3.jpg]

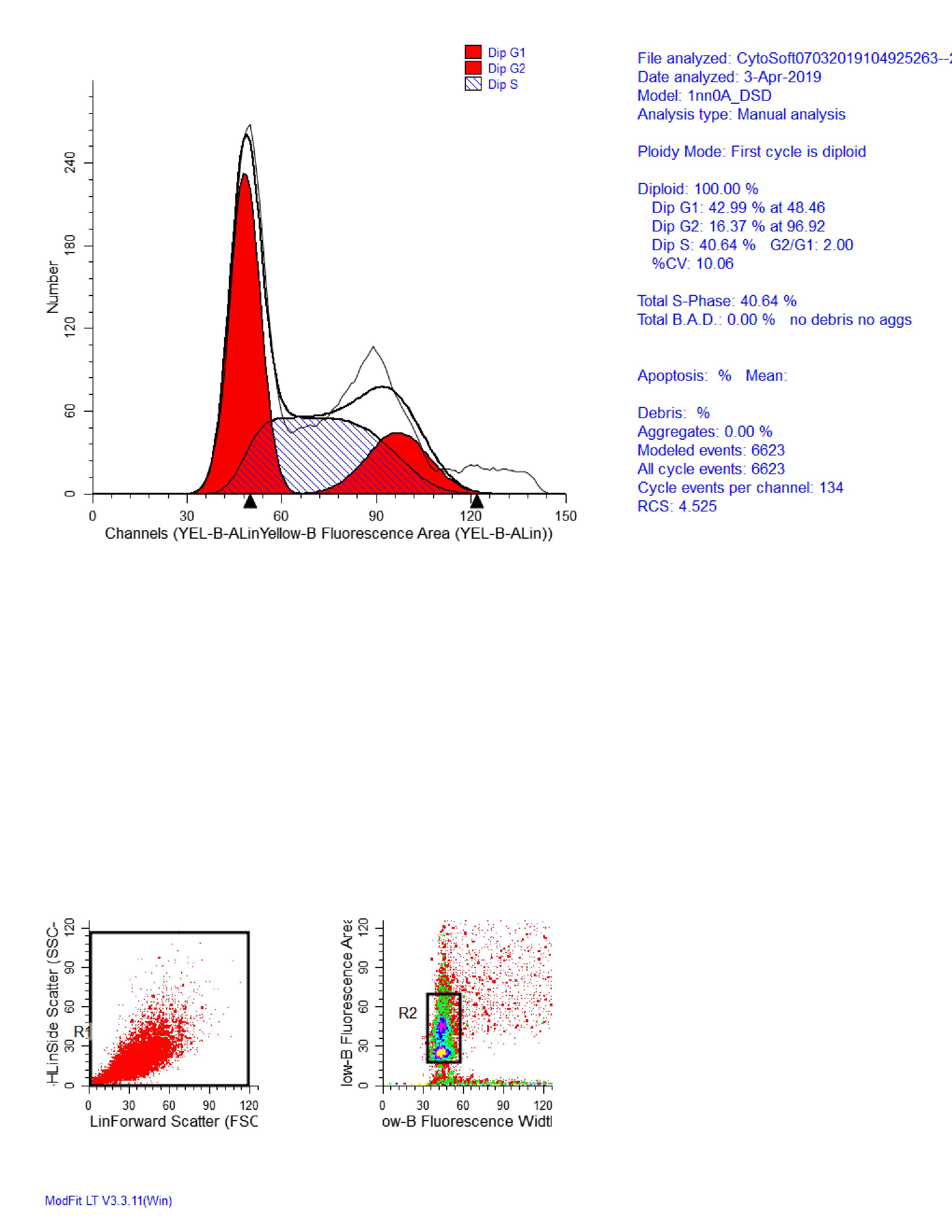

Supplement: Supplementary file 1 [file DataSheet_1.zip › Original Data 1/Figure 2D/NCI-H1299/shNLE1-1.jpg]

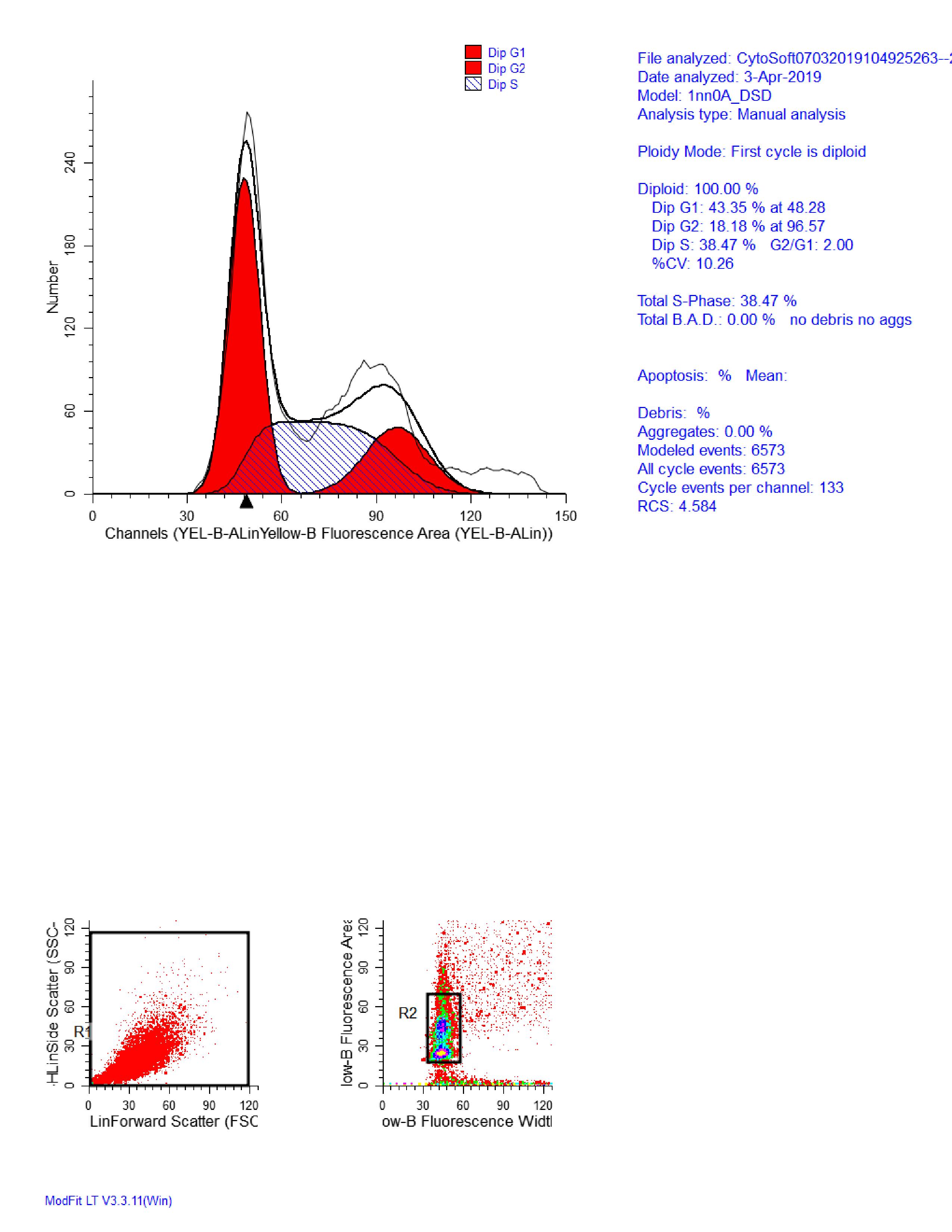

Supplement: Supplementary file 1 [file DataSheet_1.zip › Original Data 1/Figure 2D/NCI-H1299/shNLE1-2.jpg]

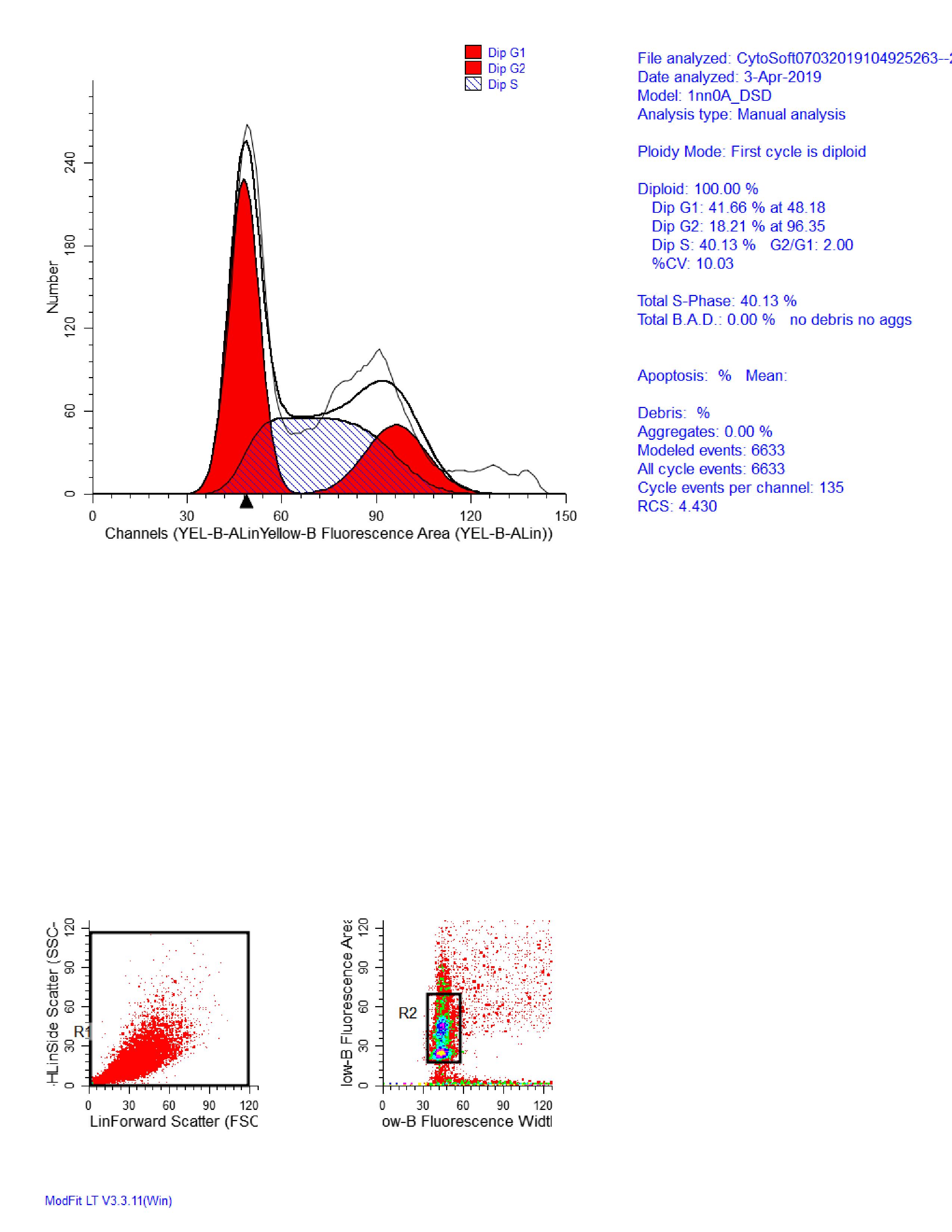

Supplement: Supplementary file 1 [file DataSheet_1.zip › Original Data 1/Figure 2D/NCI-H1299/shNLE1-3.jpg]

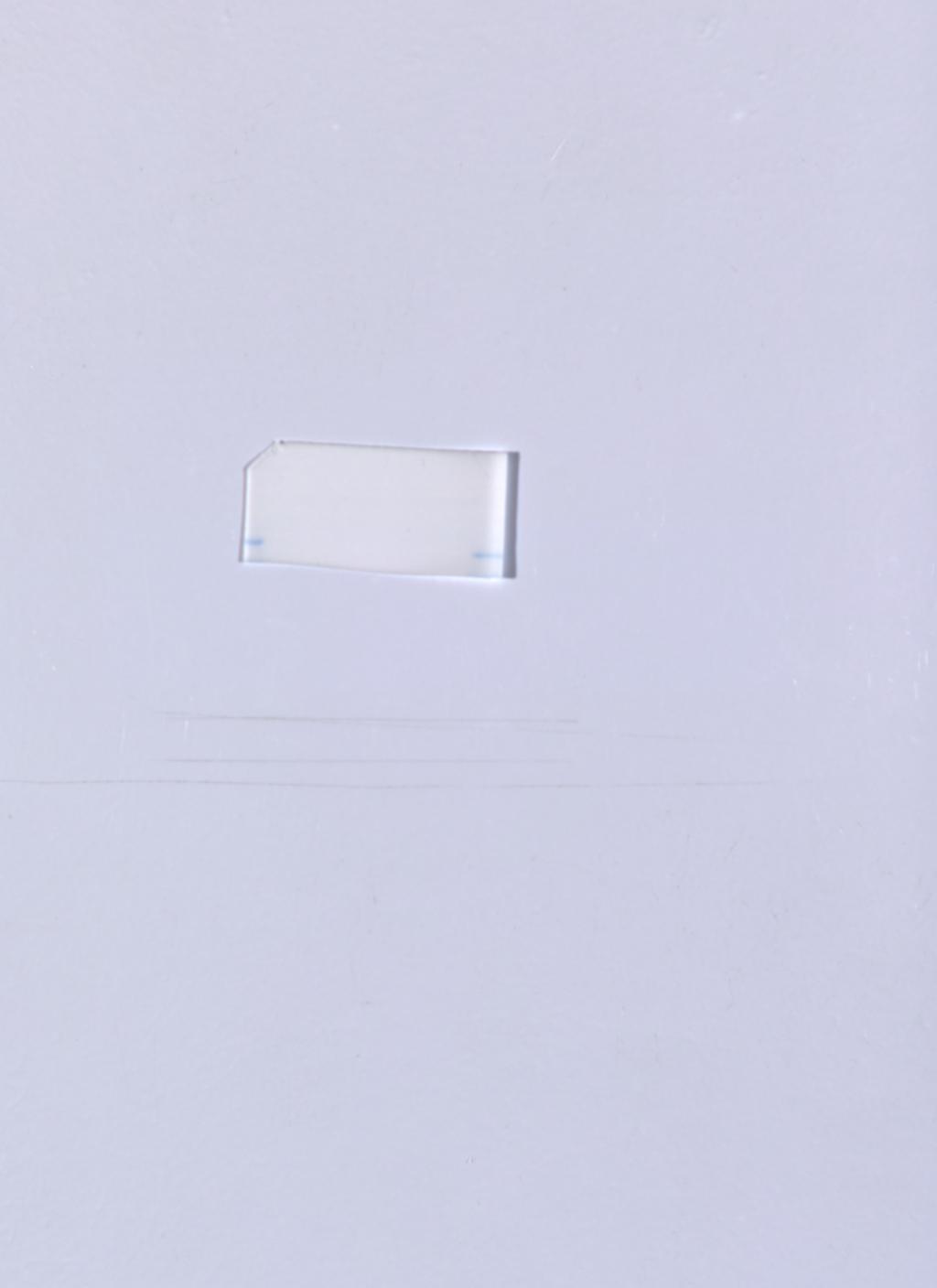

Supplement: Supplementary file 1 [file DataSheet_1.zip › Original Data 1/Figure 3D/BRCA1 M/BRCA1 M.jpg]

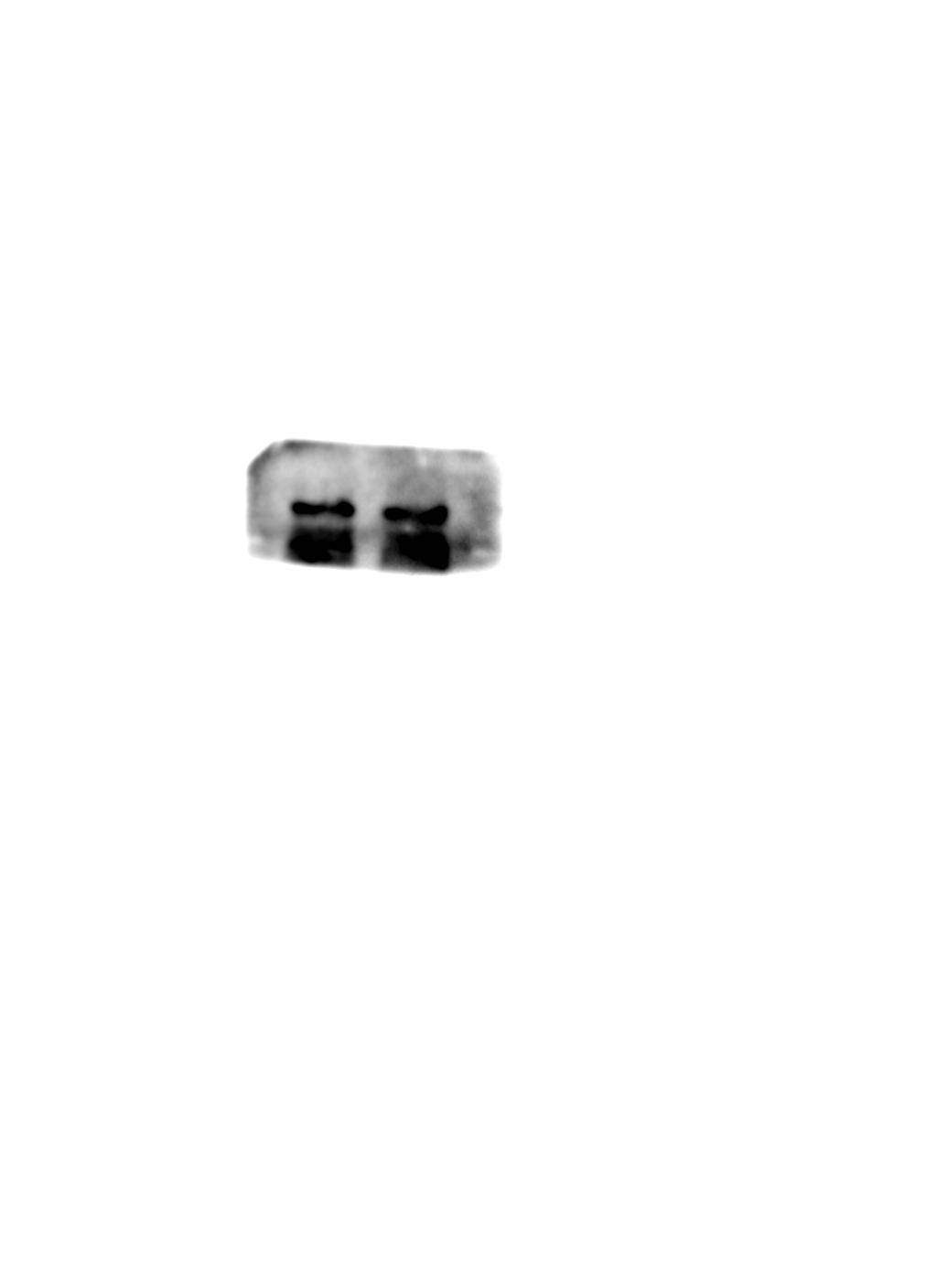

Supplement: Supplementary file 1 [file DataSheet_1.zip › Original Data 1/Figure 3D/BRCA1/BRCA1.jpg]

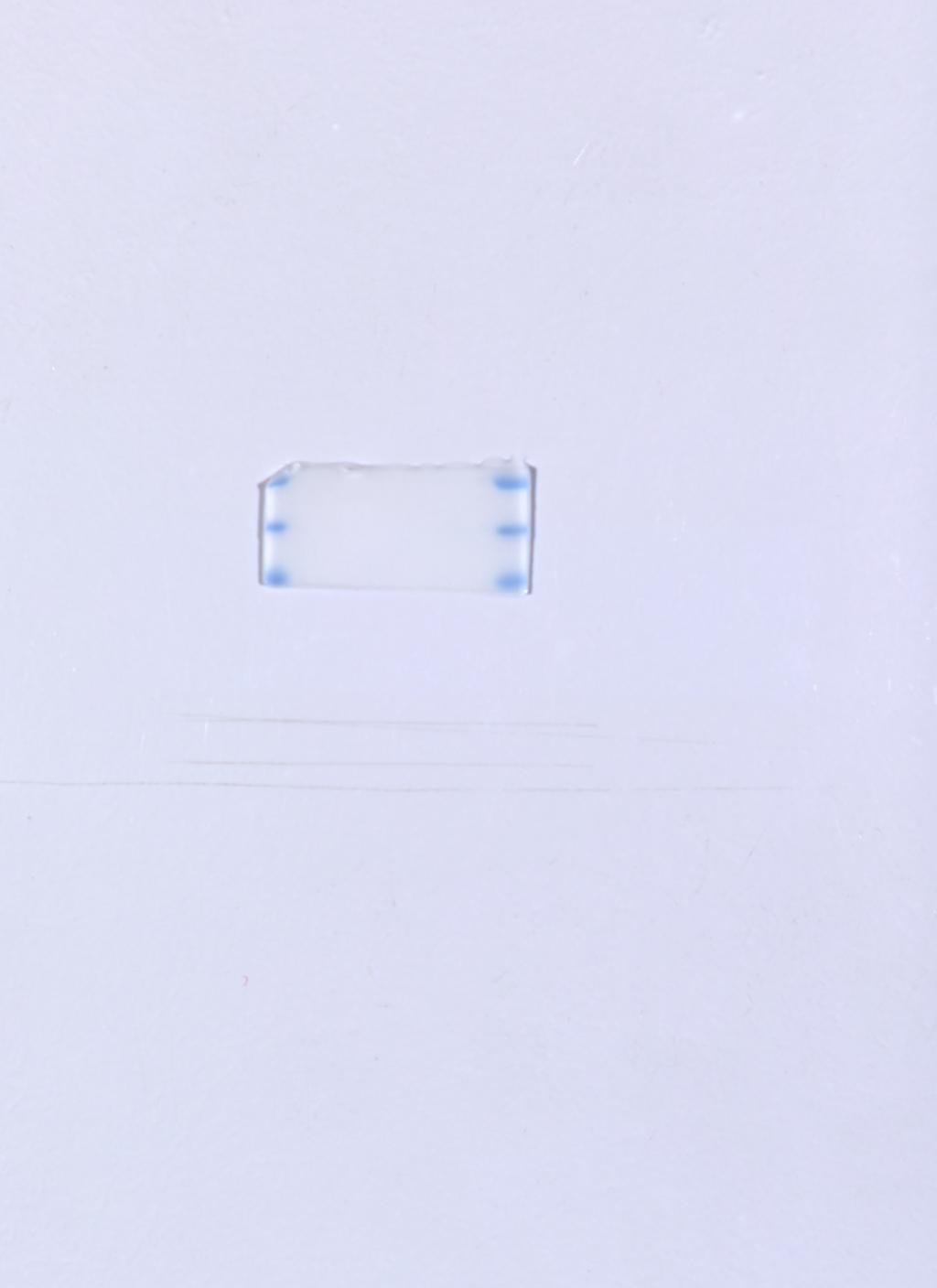

Supplement: Supplementary file 1 [file DataSheet_1.zip › Original Data 1/Figure 3D/CCNE2 M/CCNE2 M.jpg]

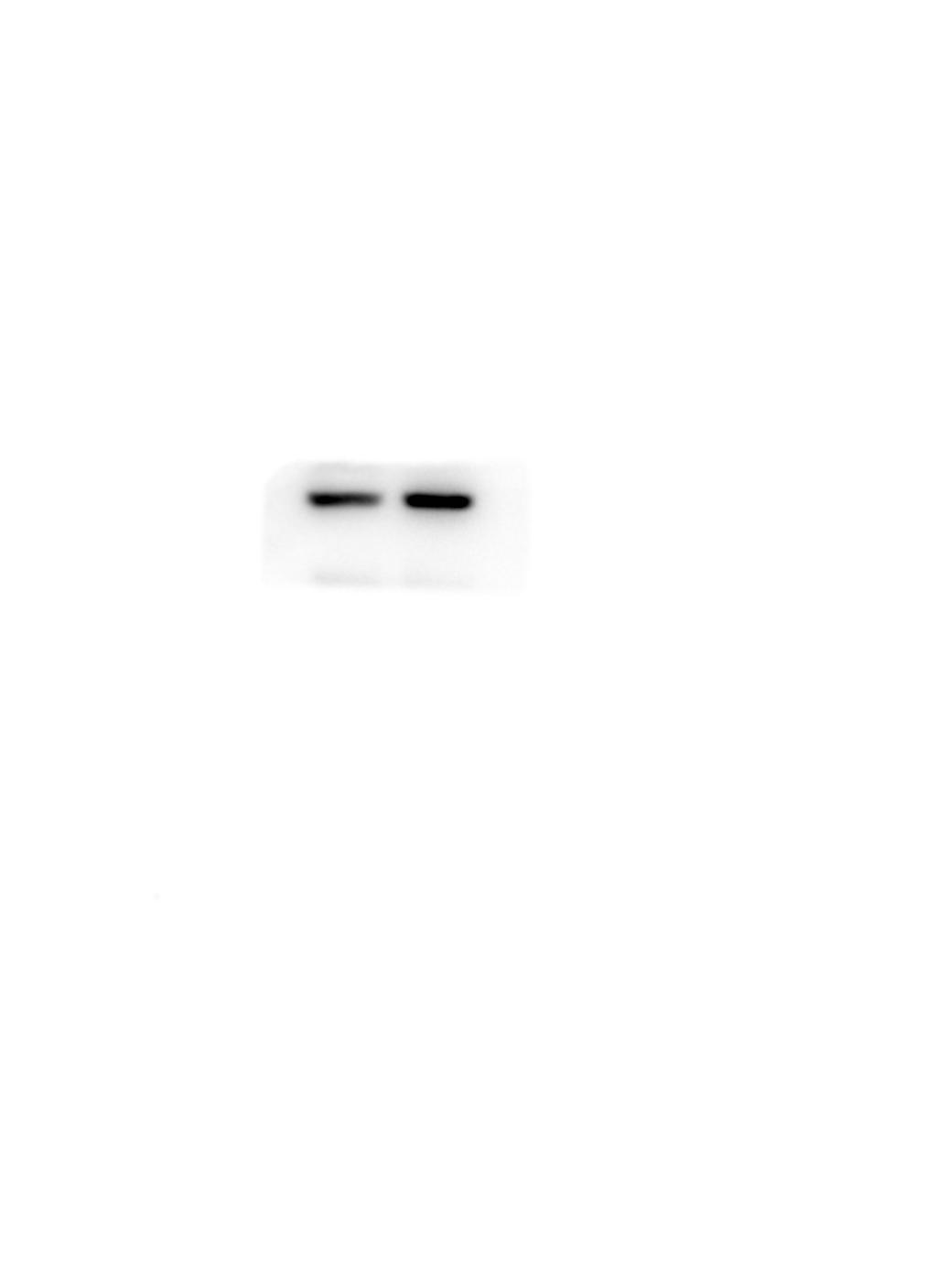

Supplement: Supplementary file 1 [file DataSheet_1.zip › Original Data 1/Figure 3D/CCNE2/CCNE2.jpg]

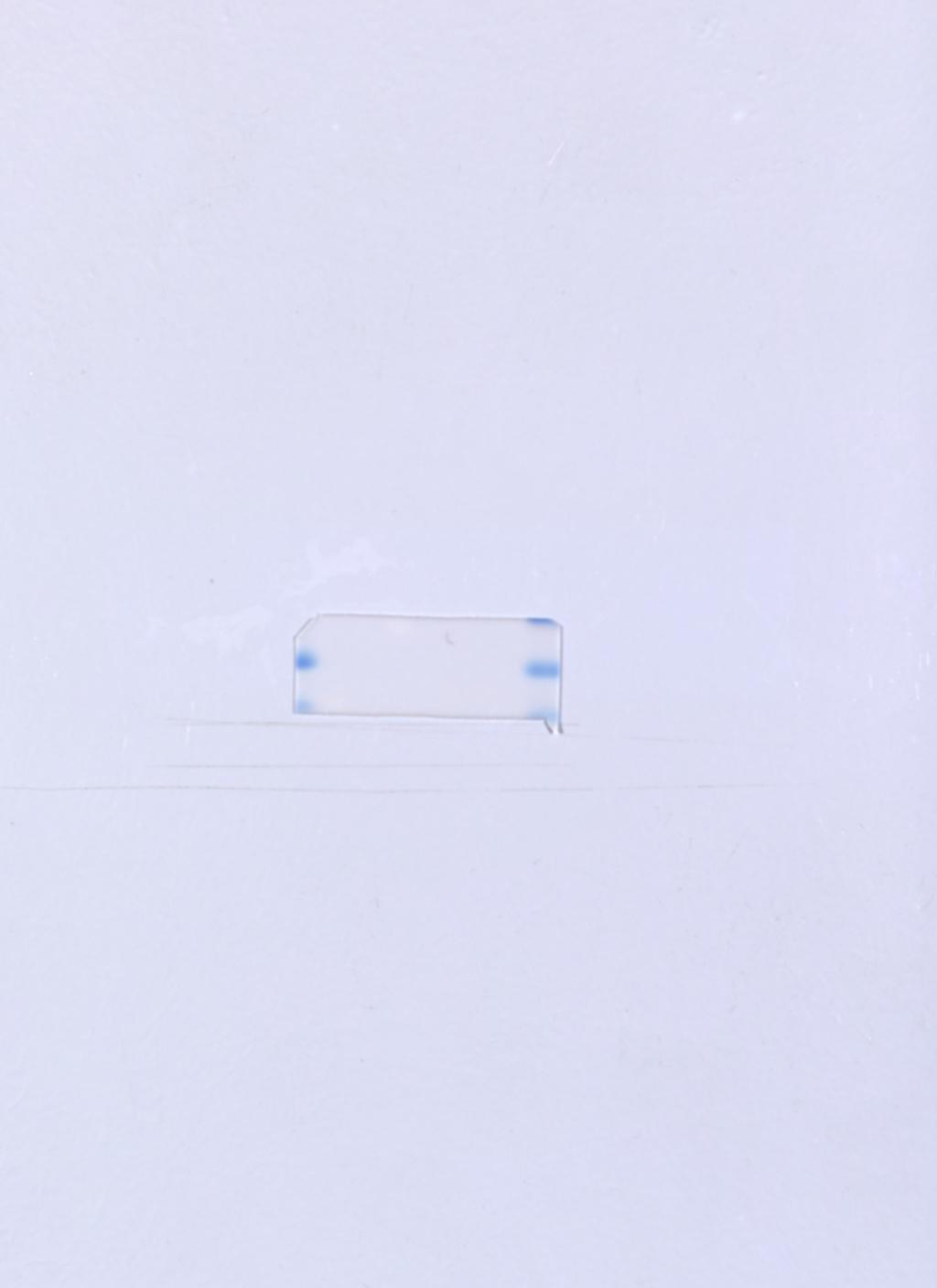

Supplement: Supplementary file 1 [file DataSheet_1.zip › Original Data 1/Figure 3D/CDK1 M/CDK1 M.jpg]

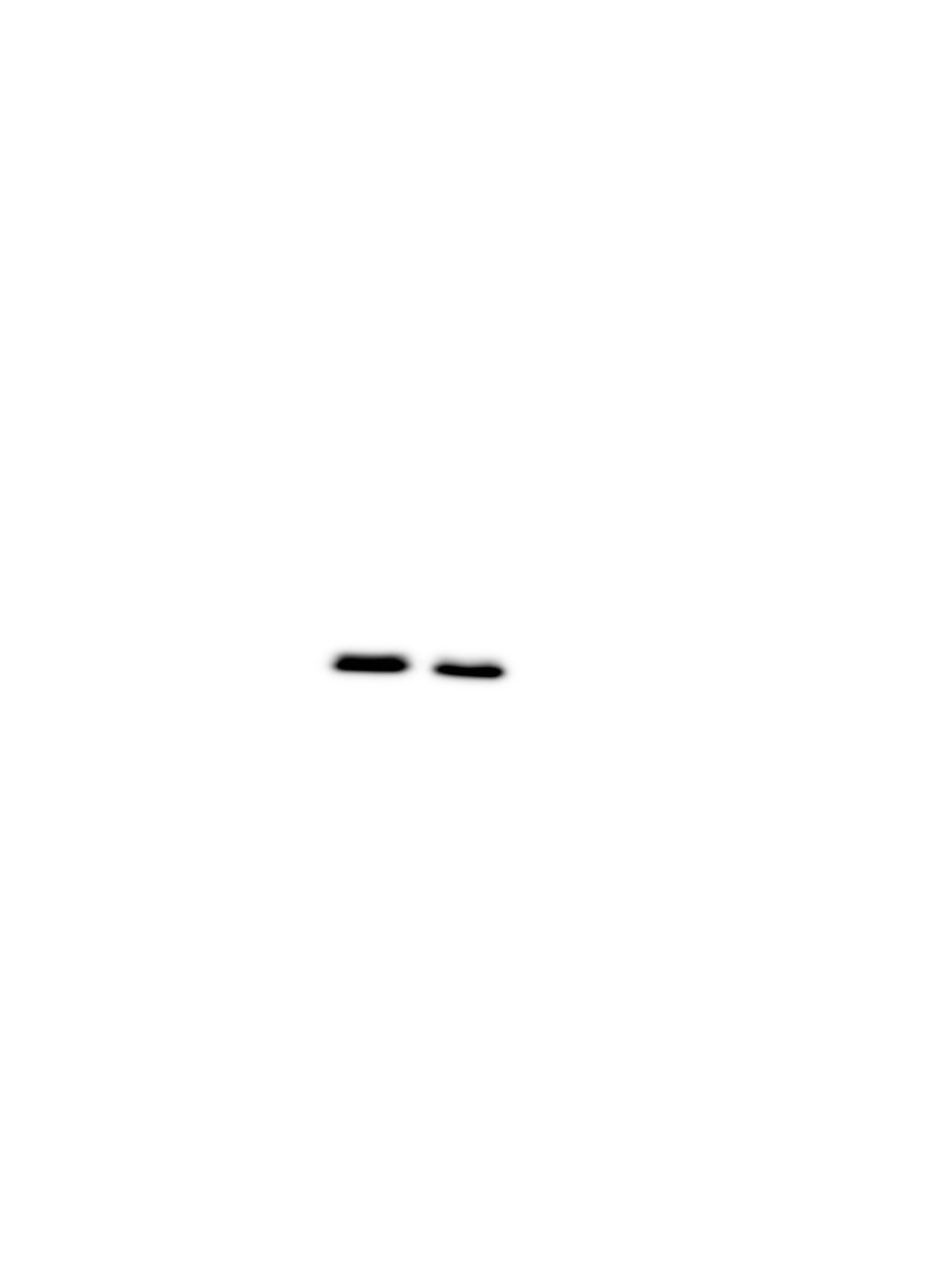

Supplement: Supplementary file 1 [file DataSheet_1.zip › Original Data 1/Figure 3D/CDK1/CDK1.jpg]

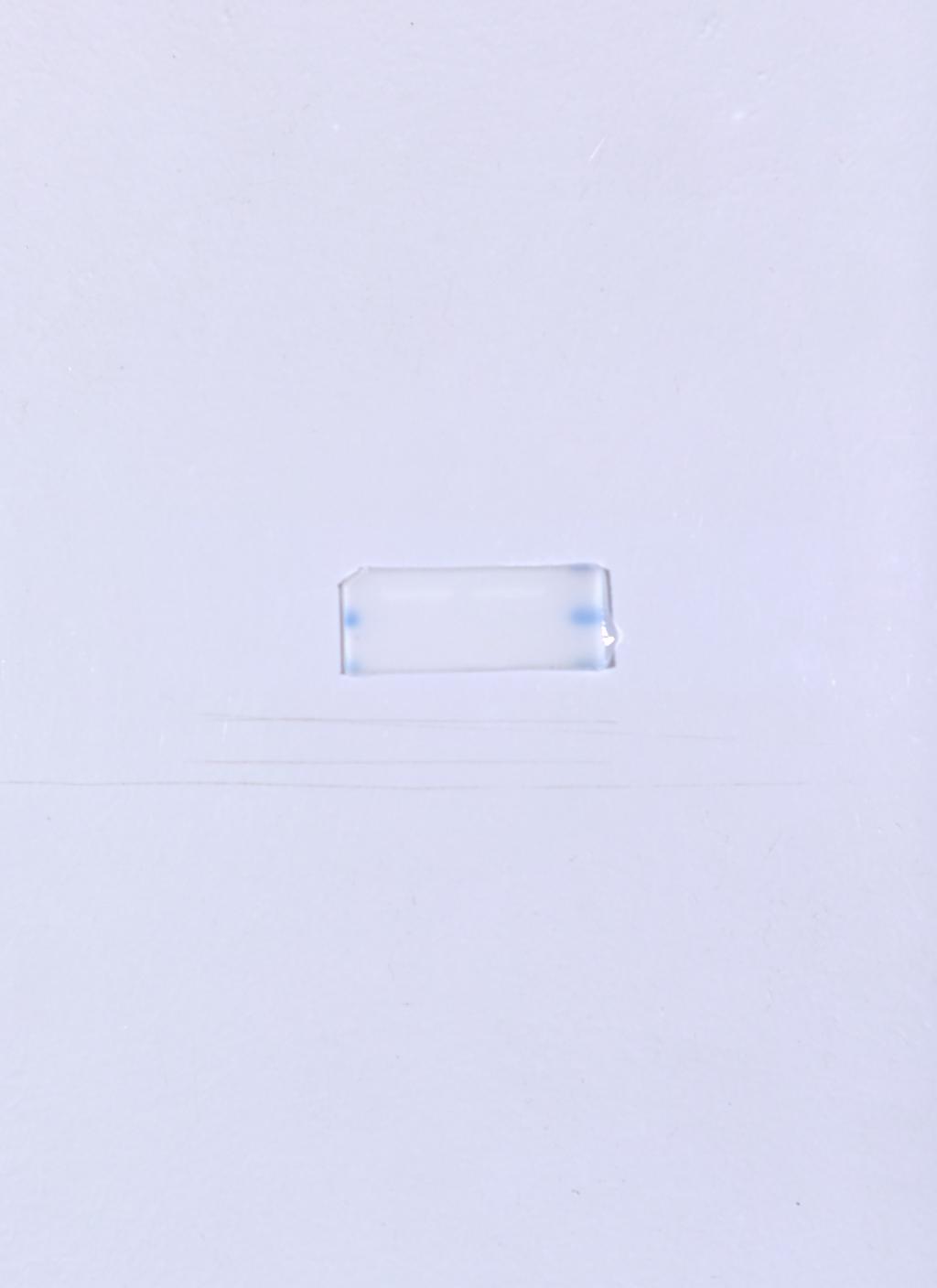

Supplement: Supplementary file 1 [file DataSheet_1.zip › Original Data 1/Figure 3D/GAPDH M/GAPDH M.jpg]

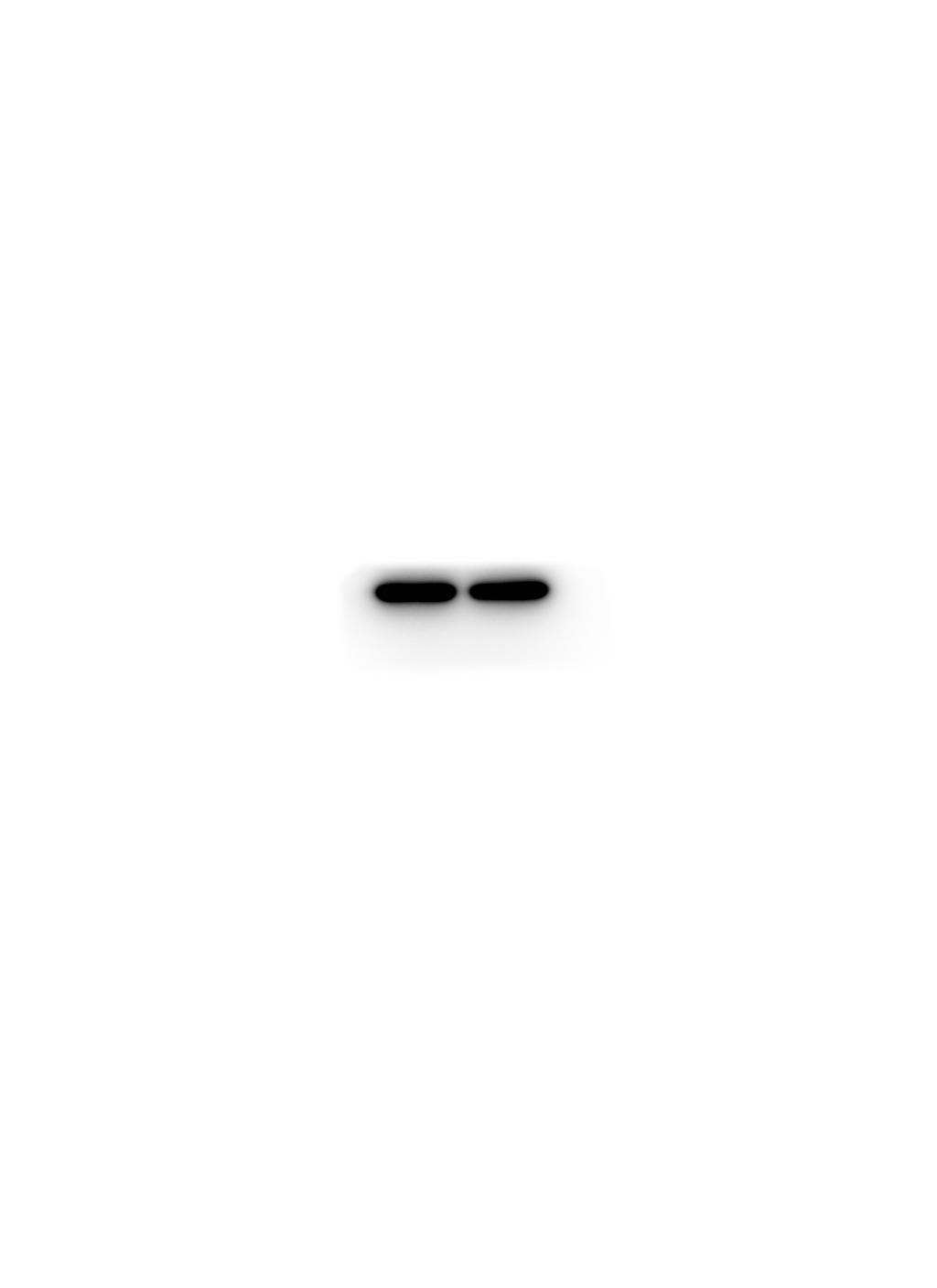

Supplement: Supplementary file 1 [file DataSheet_1.zip › Original Data 1/Figure 3D/GAPDH/GAPDH.jpg]

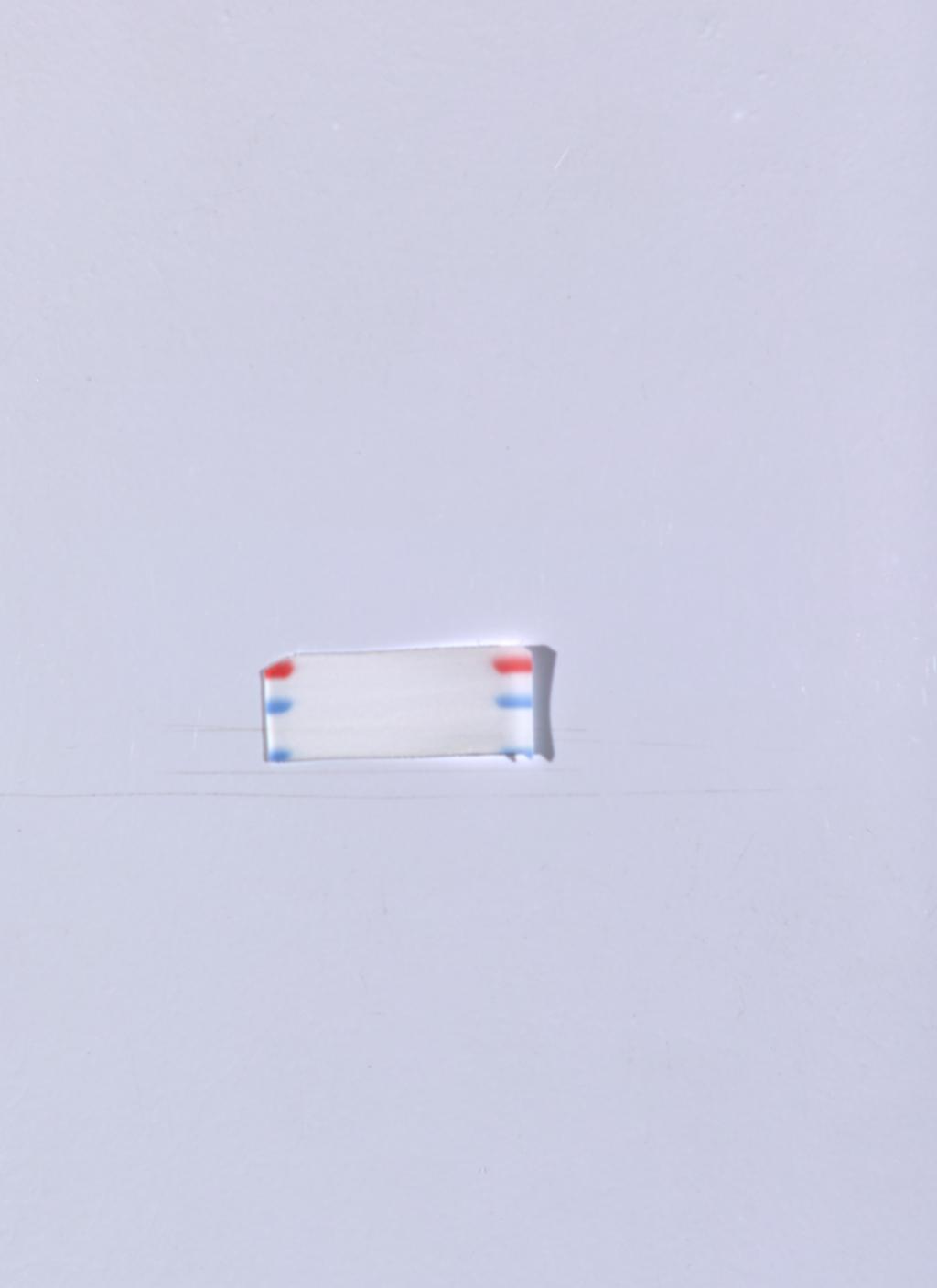

Supplement: Supplementary file 1 [file DataSheet_1.zip › Original Data 1/Figure 3D/PLK1 M/PLK1 M.jpg]

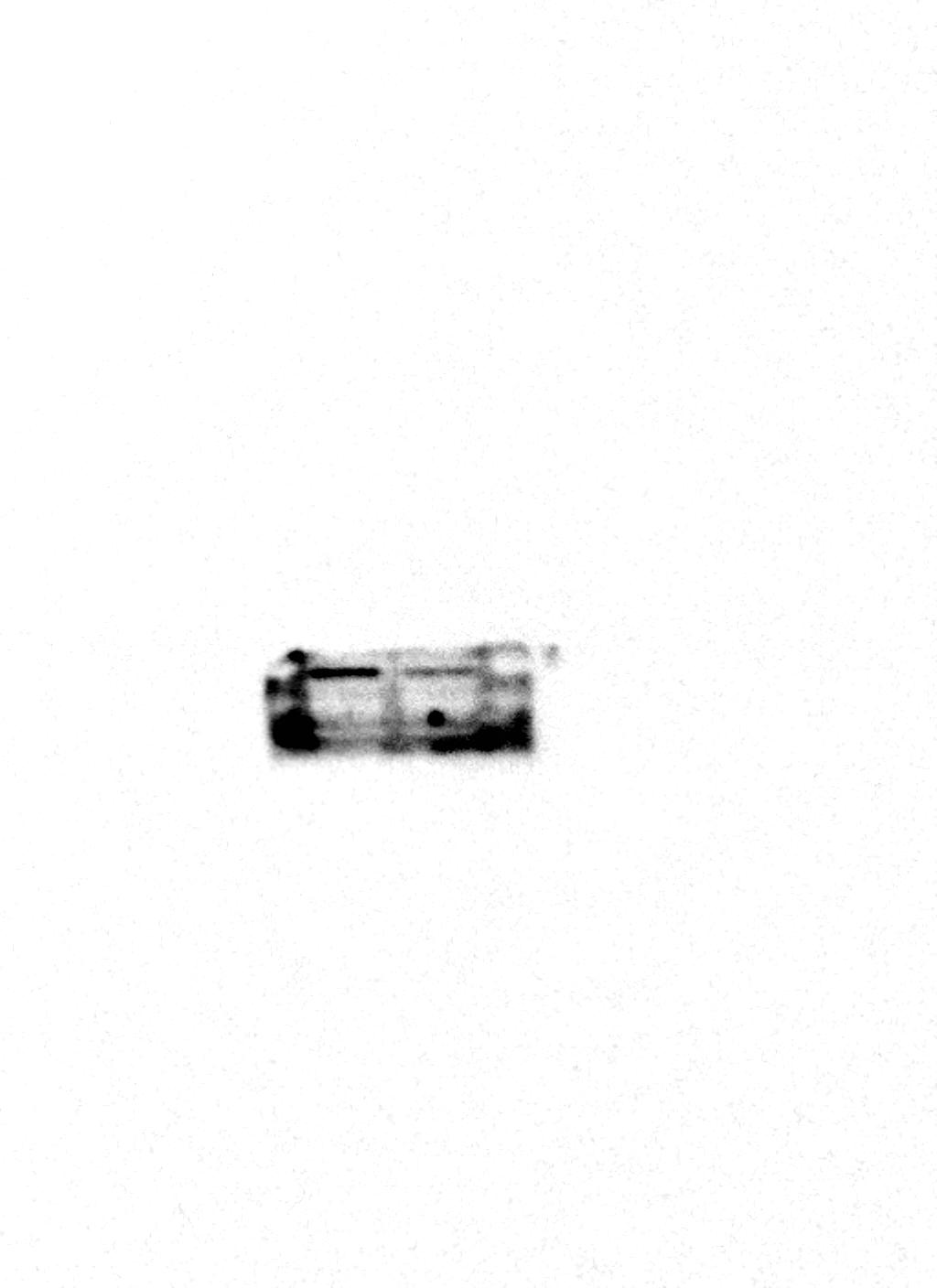

Supplement: Supplementary file 1 [file DataSheet_1.zip › Original Data 1/Figure 3D/PLK1/PLK1.jpg]

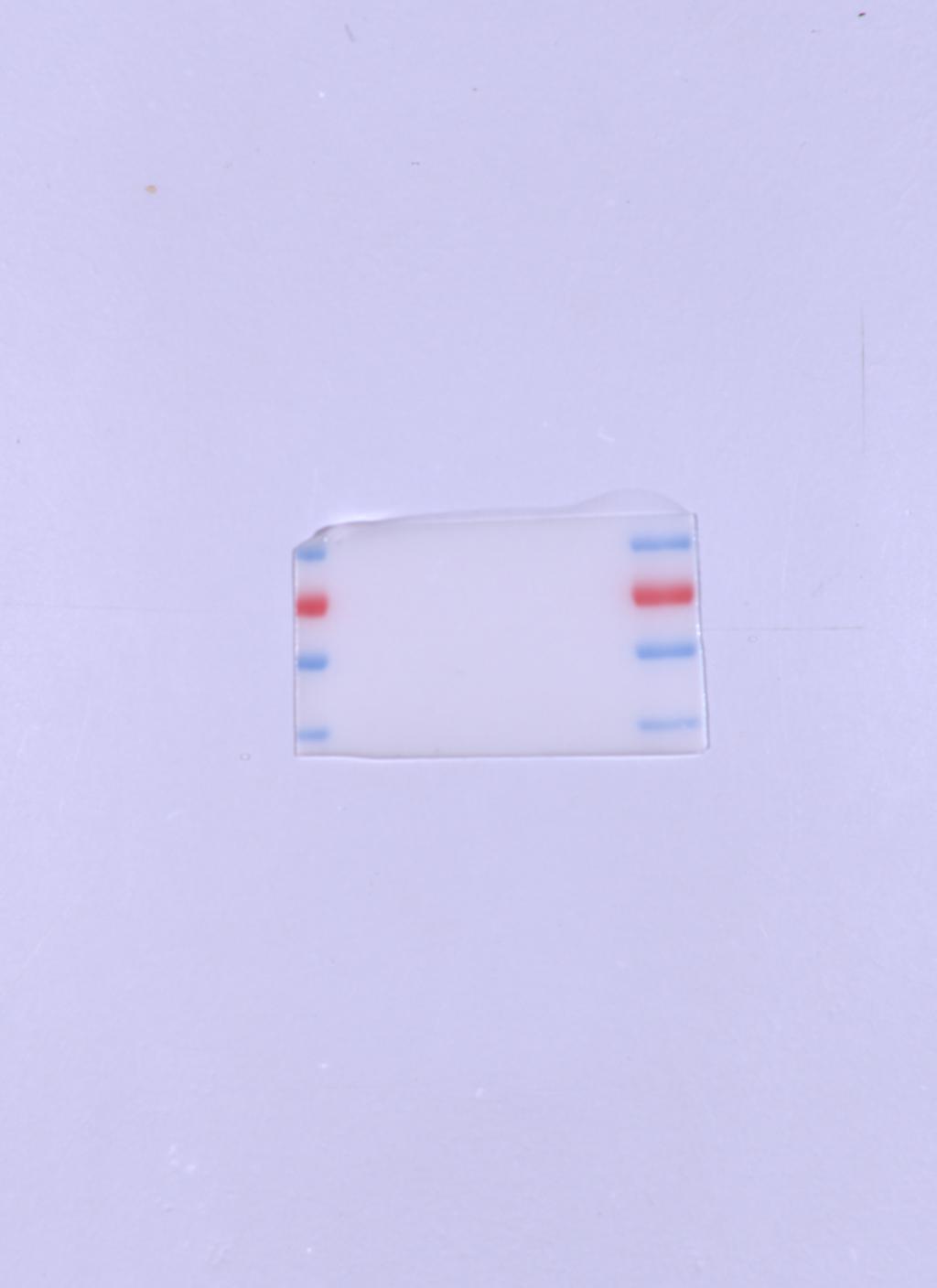

Supplement: Supplementary file 1 [file DataSheet_1.zip › Original Data 1/Figure 3E/E2F1 M/E2F1 M.jpg]

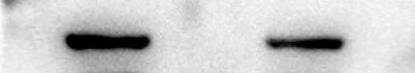

Supplement: Supplementary file 1 [file DataSheet_1.zip › Original Data 1/Figure 3E/E2F1.jpg]

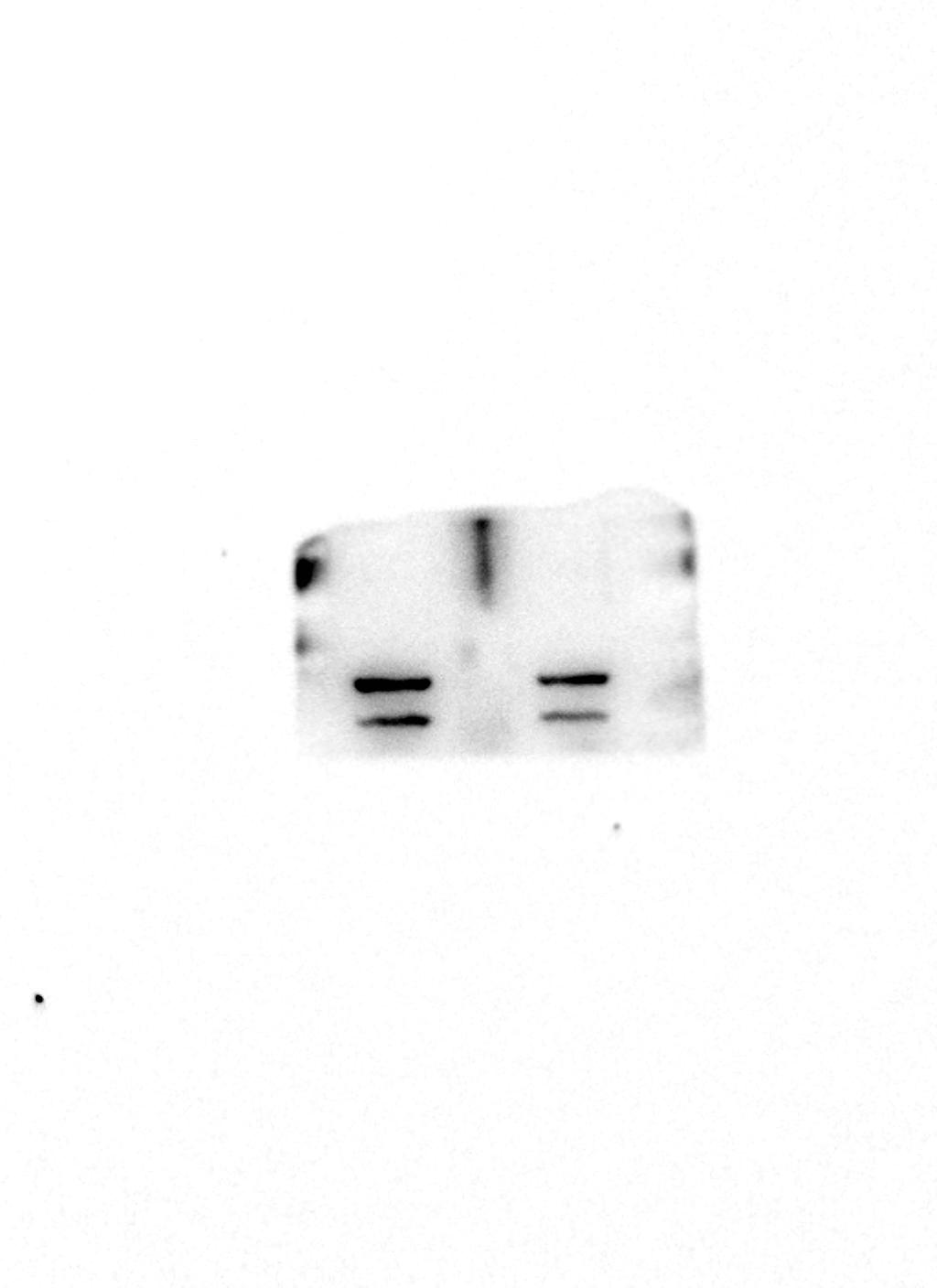

Supplement: Supplementary file 1 [file DataSheet_1.zip › Original Data 1/Figure 3E/E2F1/E2F1.jpg]

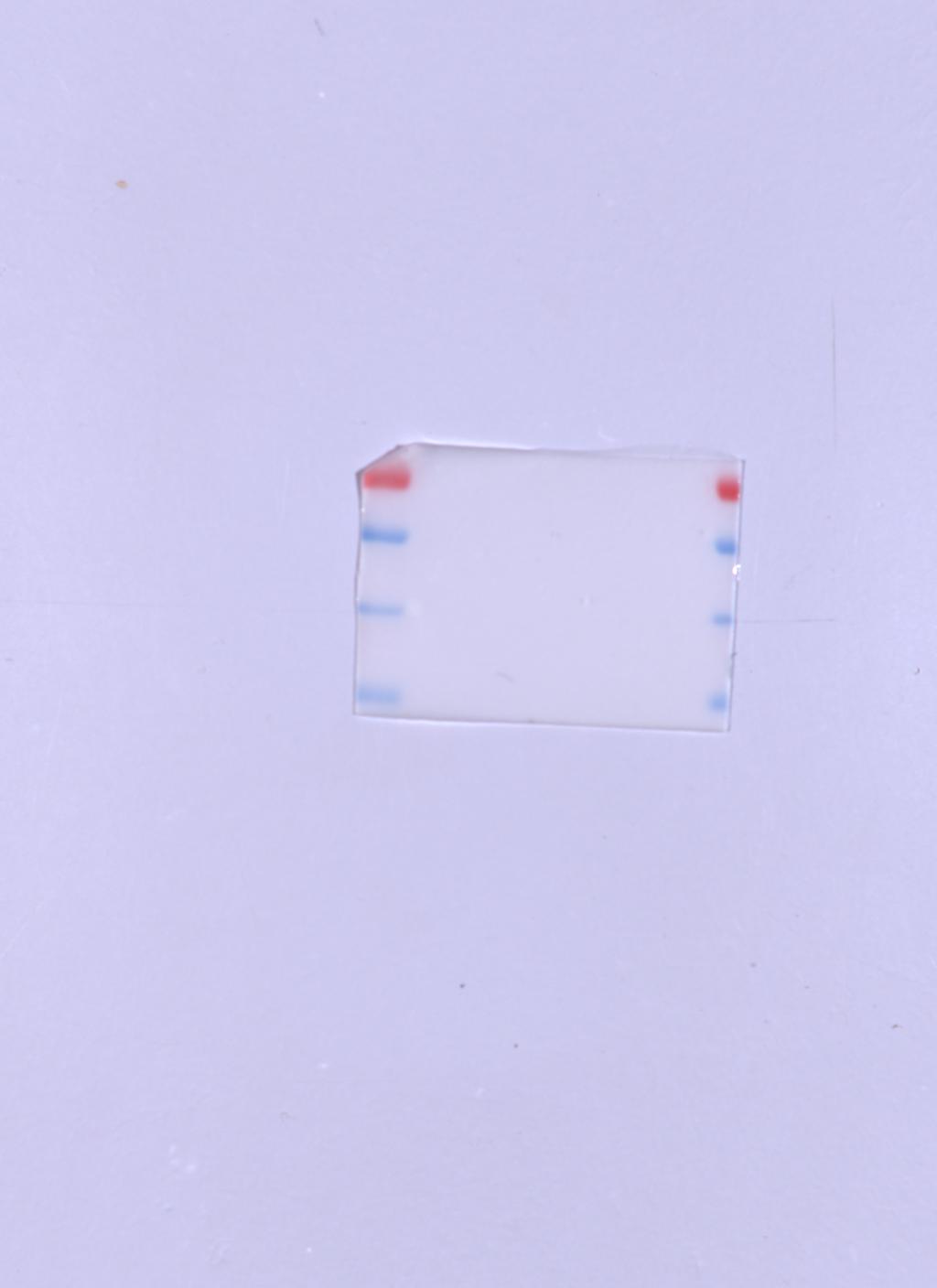

Supplement: Supplementary file 1 [file DataSheet_1.zip › Original Data 1/Figure 3E/NLE1 M/NLE1 M.jpg]

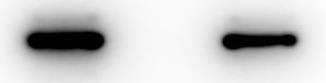

Supplement: Supplementary file 1 [file DataSheet_1.zip › Original Data 1/Figure 3E/NLE1.jpg]

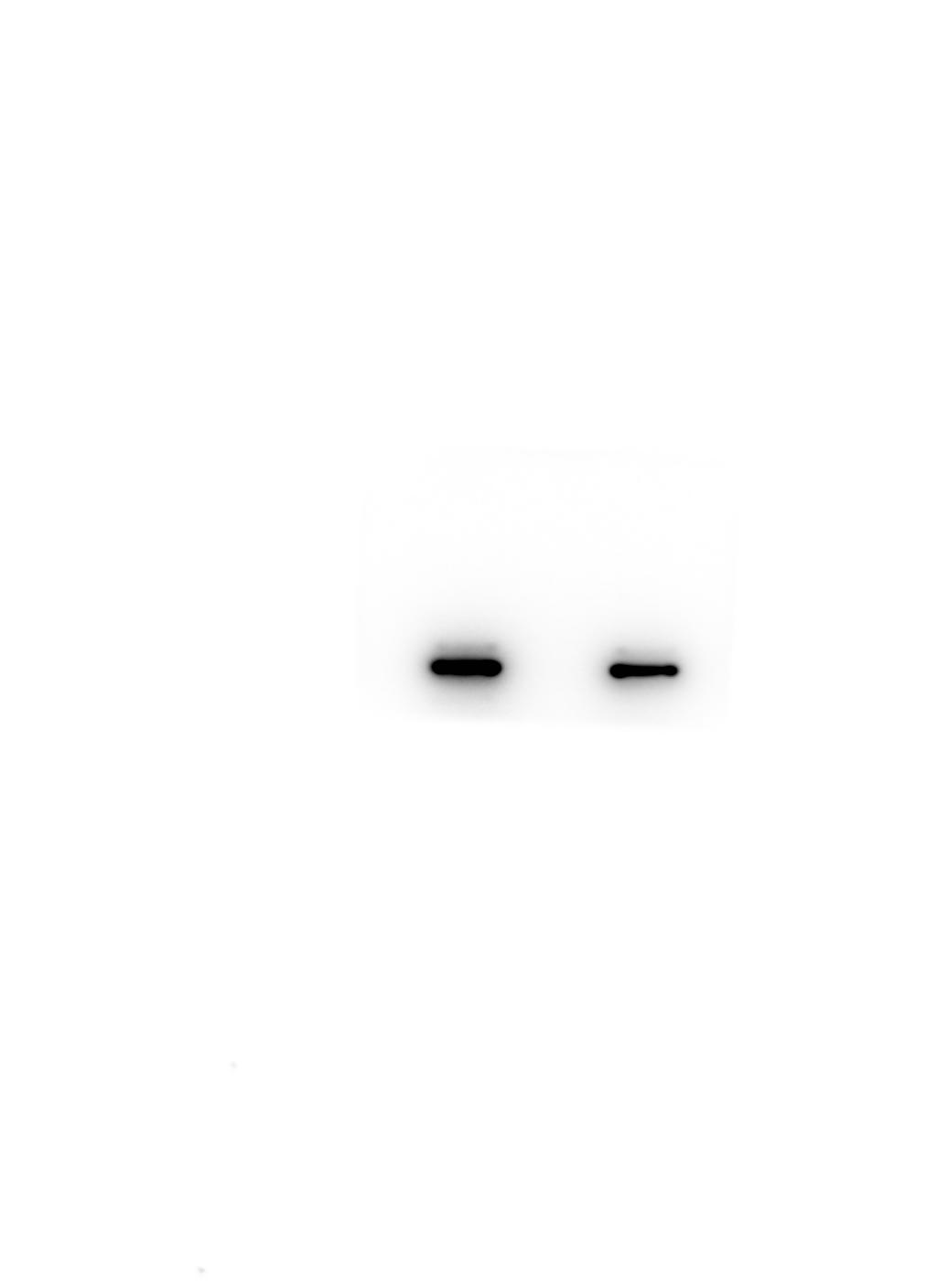

Supplement: Supplementary file 1 [file DataSheet_1.zip › Original Data 1/Figure 3E/NLE1/NLE1.jpg]

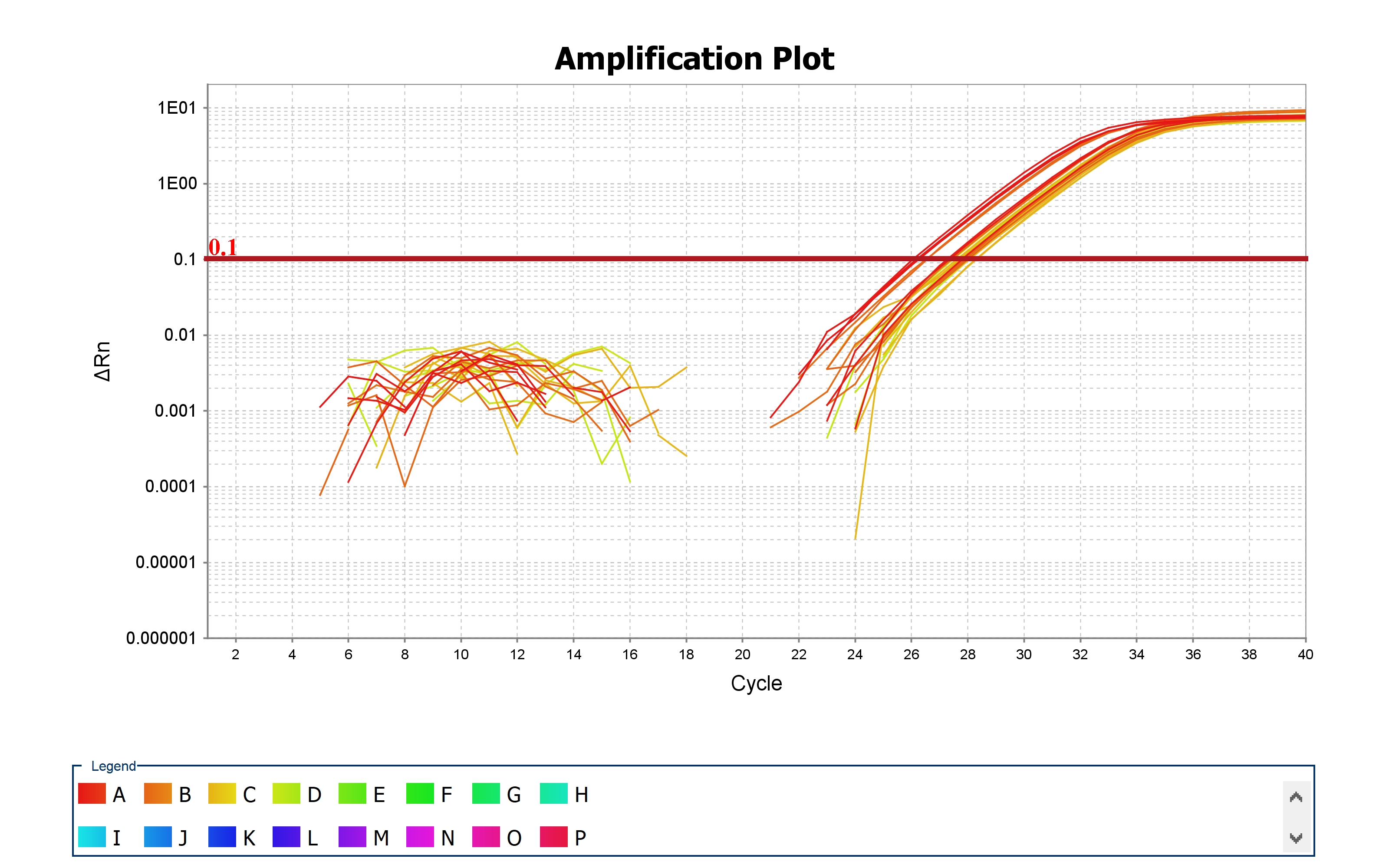

Supplement: Supplementary file 2 [file DataSheet_2.zip › Original Data 2/Figure 3F/H-CDK1-1 Amplification Plot.jpg]

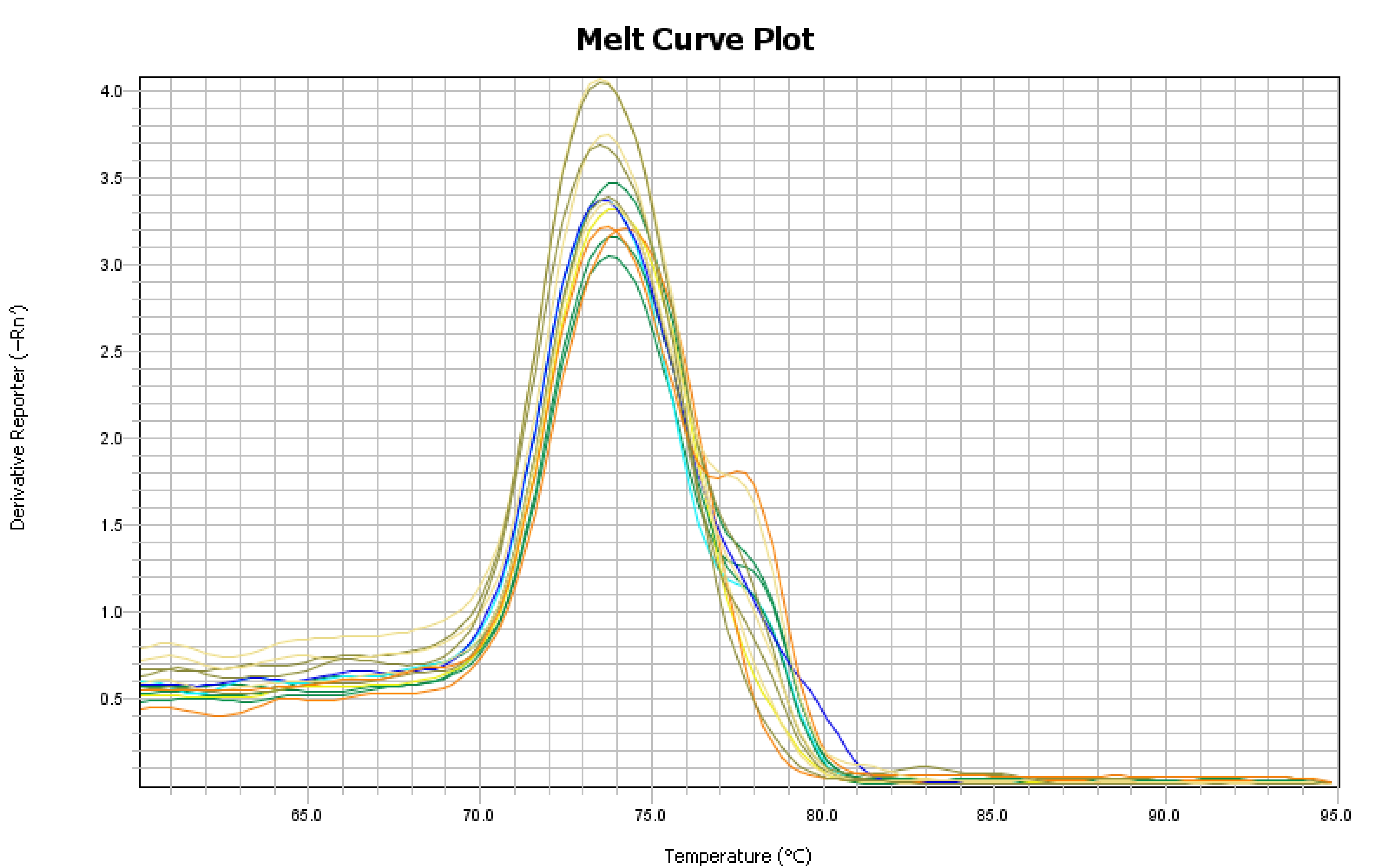

Supplement: Supplementary file 2 [file DataSheet_2.zip › Original Data 2/Figure 3F/H-CDK1-1 Melt Curve Plot.jpg]

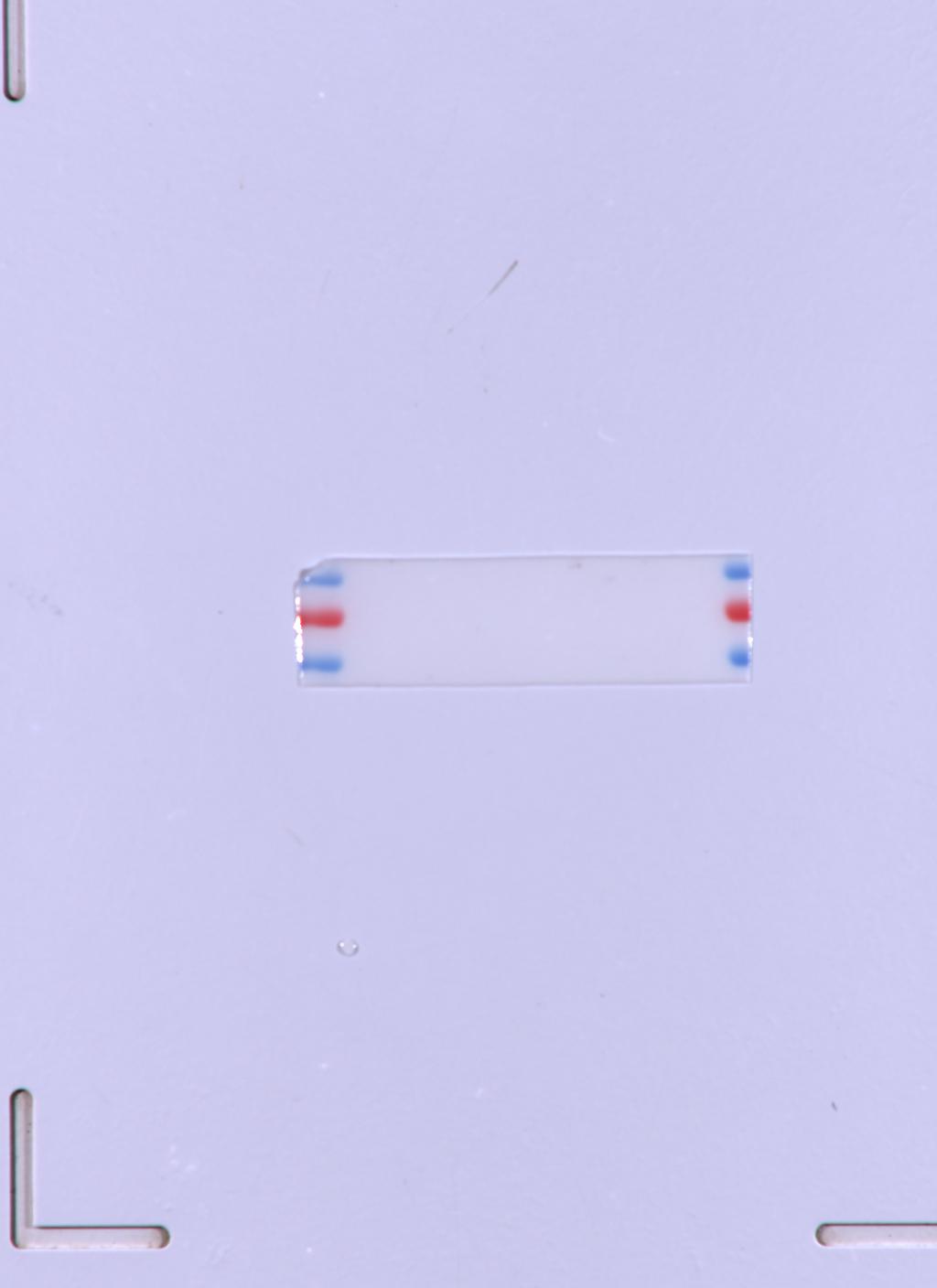

Supplement: Supplementary file 2 [file DataSheet_2.zip › Original Data 2/Figure 4D/A549/AKT M/AKT M.jpg]

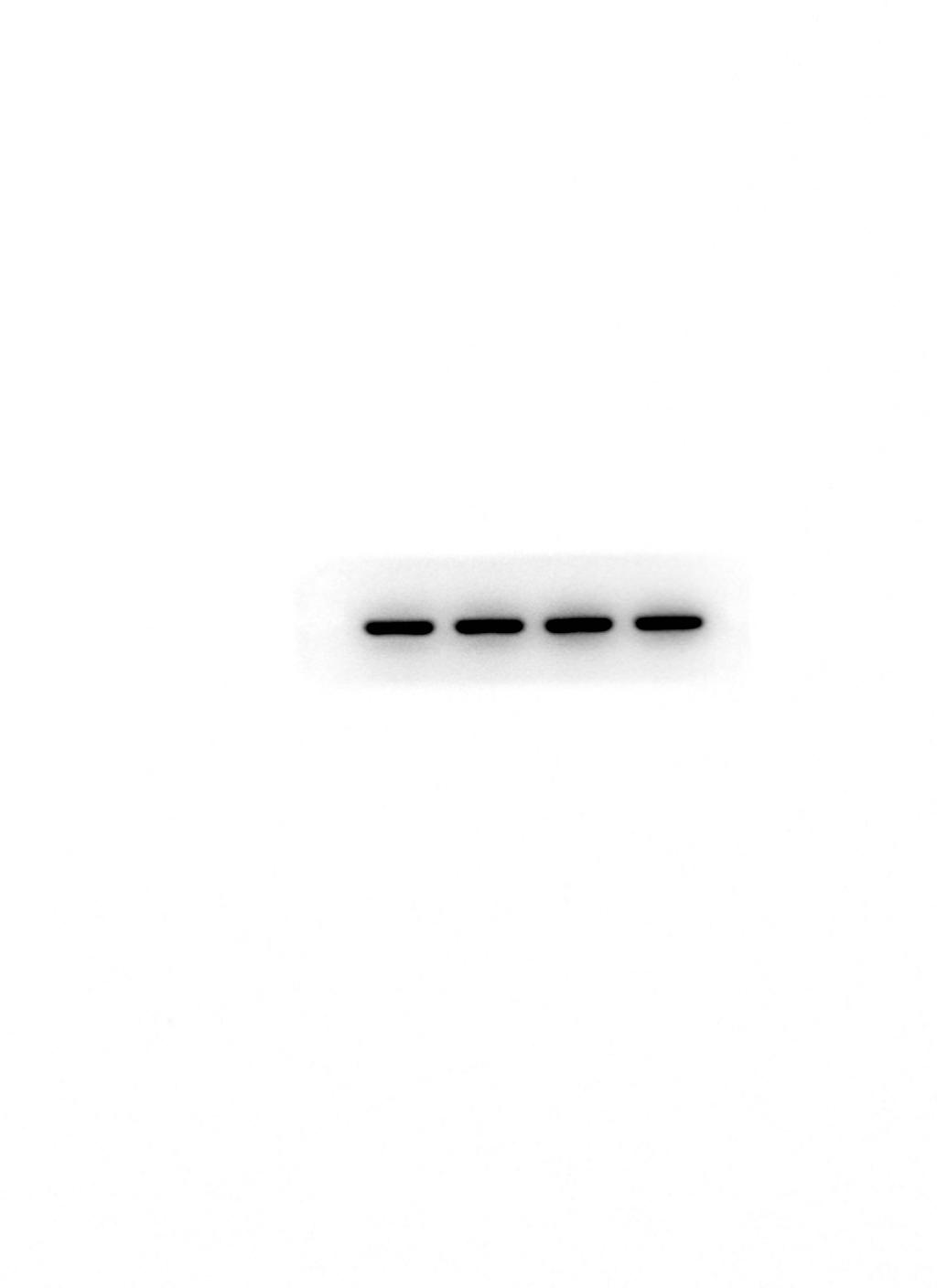

Supplement: Supplementary file 2 [file DataSheet_2.zip › Original Data 2/Figure 4D/A549/AKT/AKT.jpg]

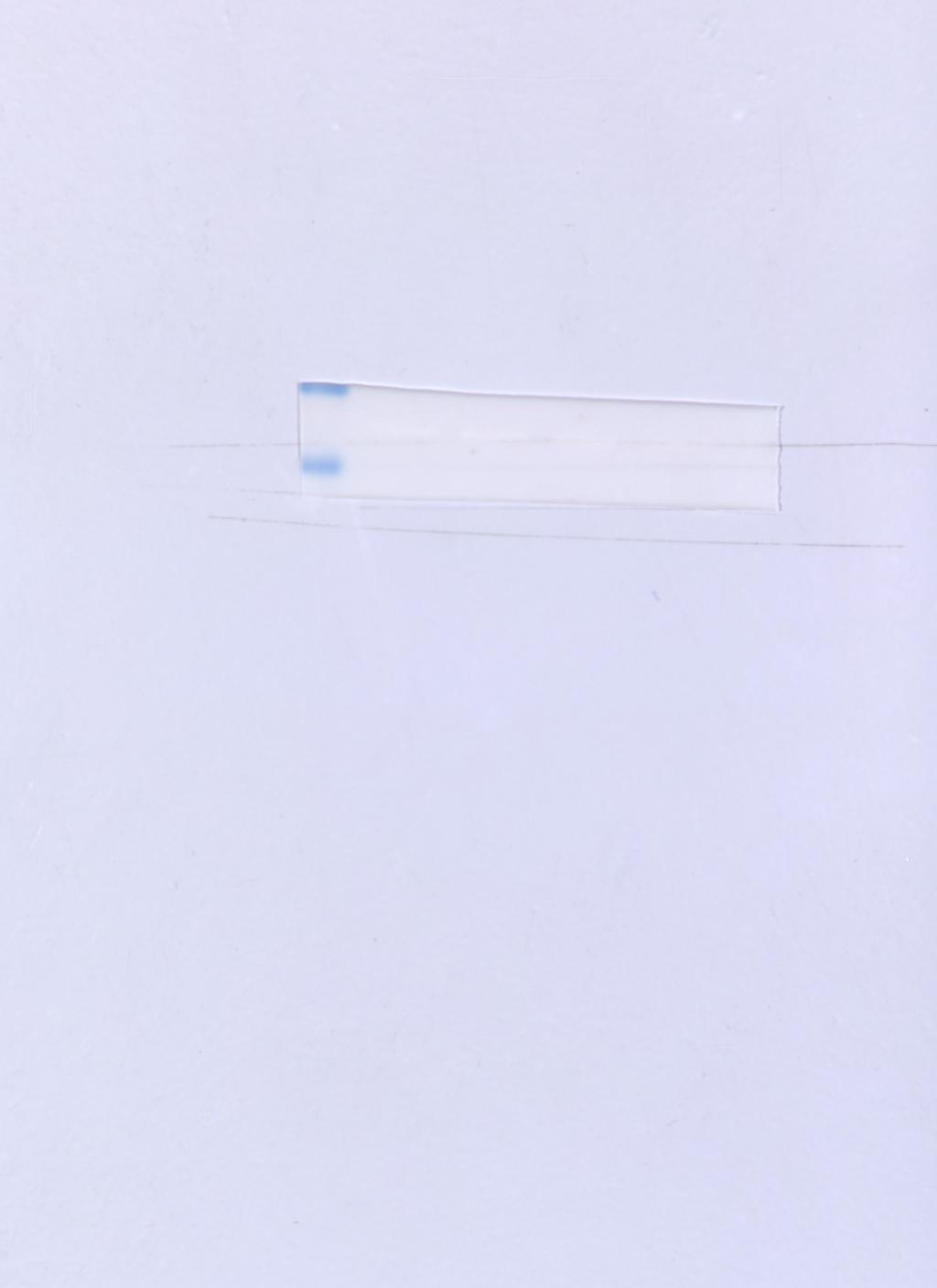

Supplement: Supplementary file 2 [file DataSheet_2.zip › Original Data 2/Figure 4D/A549/GAPDH M/GAPDH M.jpg]

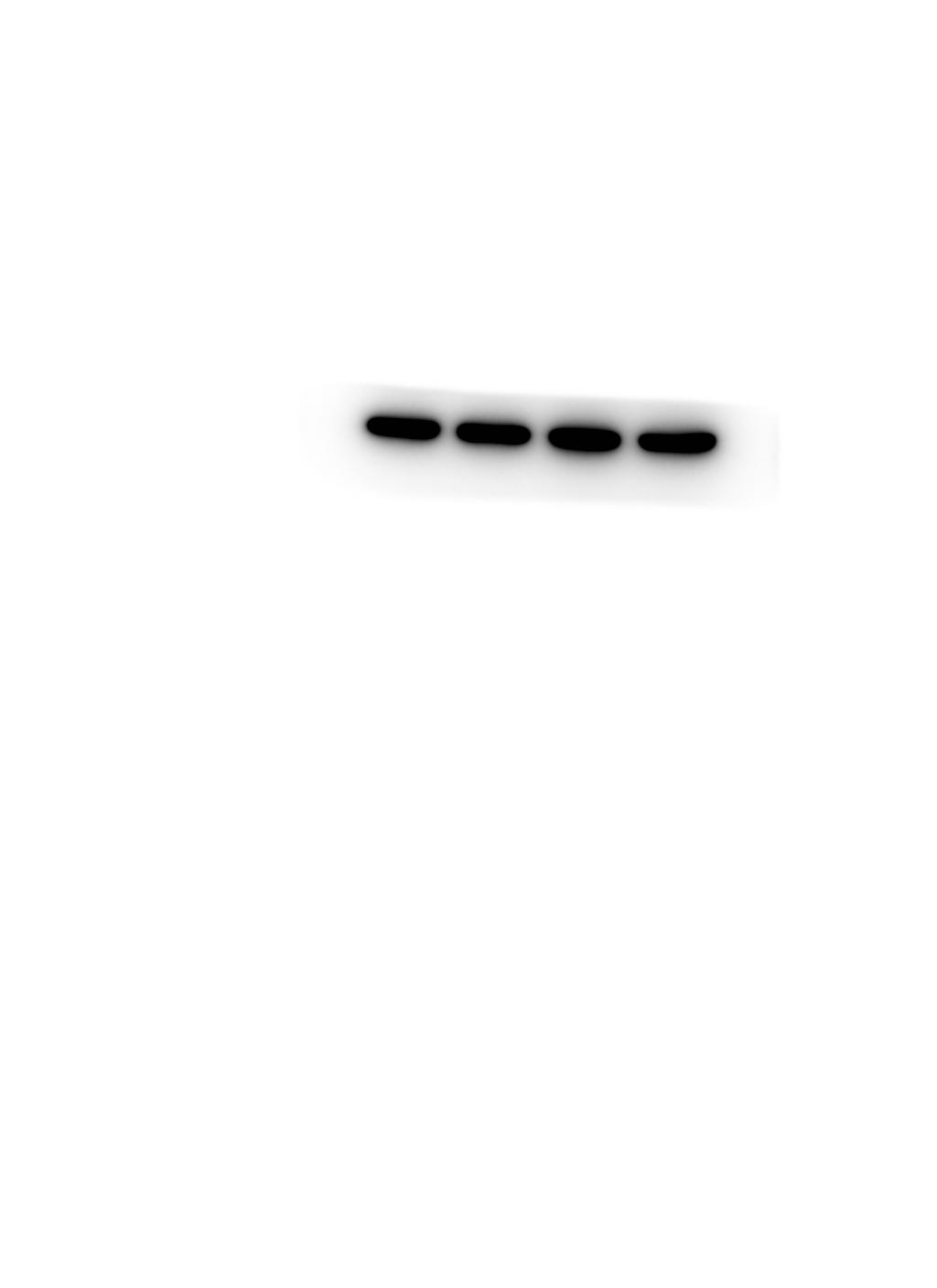

Supplement: Supplementary file 2 [file DataSheet_2.zip › Original Data 2/Figure 4D/A549/GAPDH/GAPDH.jpg]

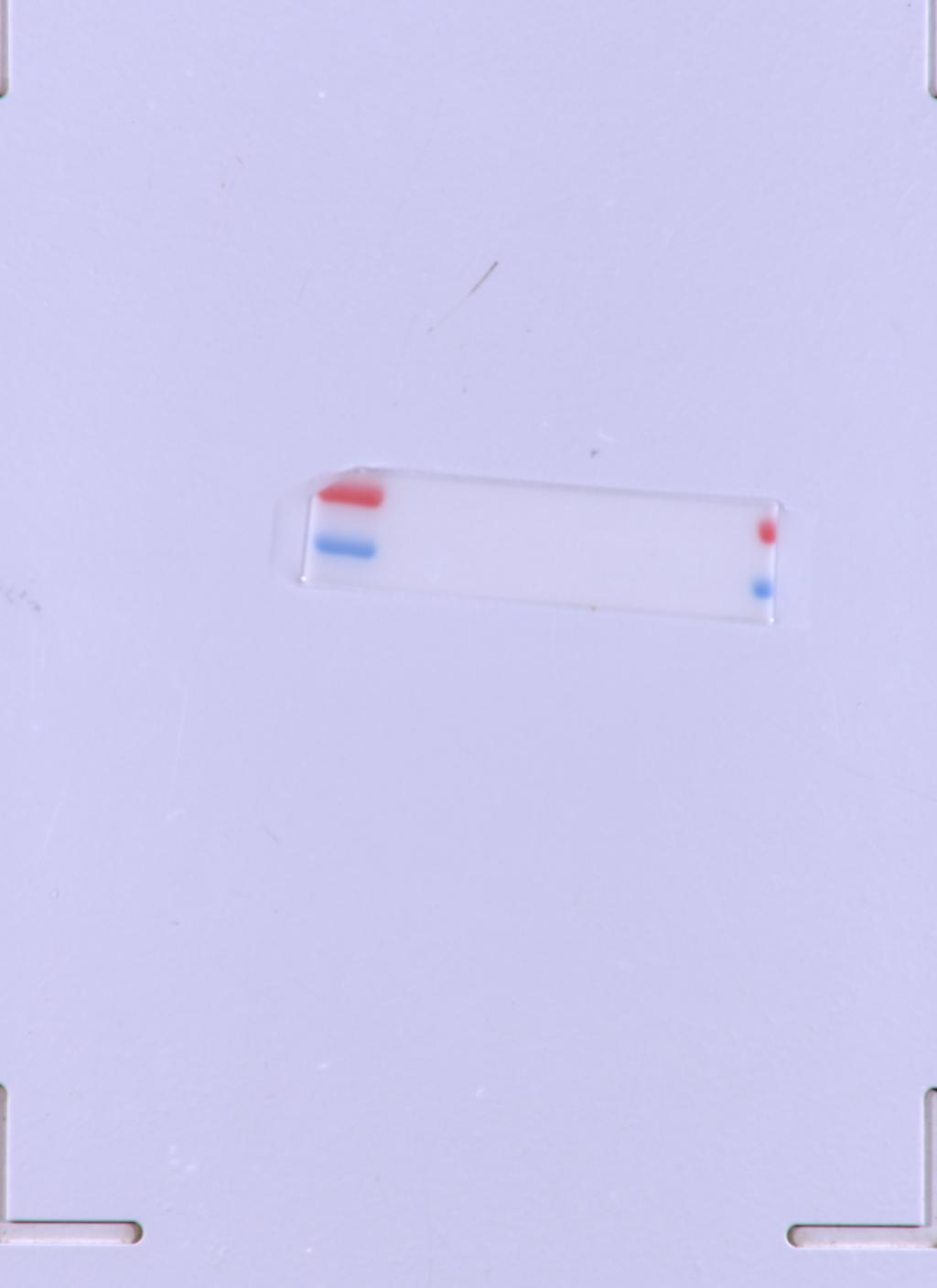

Supplement: Supplementary file 2 [file DataSheet_2.zip › Original Data 2/Figure 4D/A549/p-AKT M/p-AKT M.jpg]

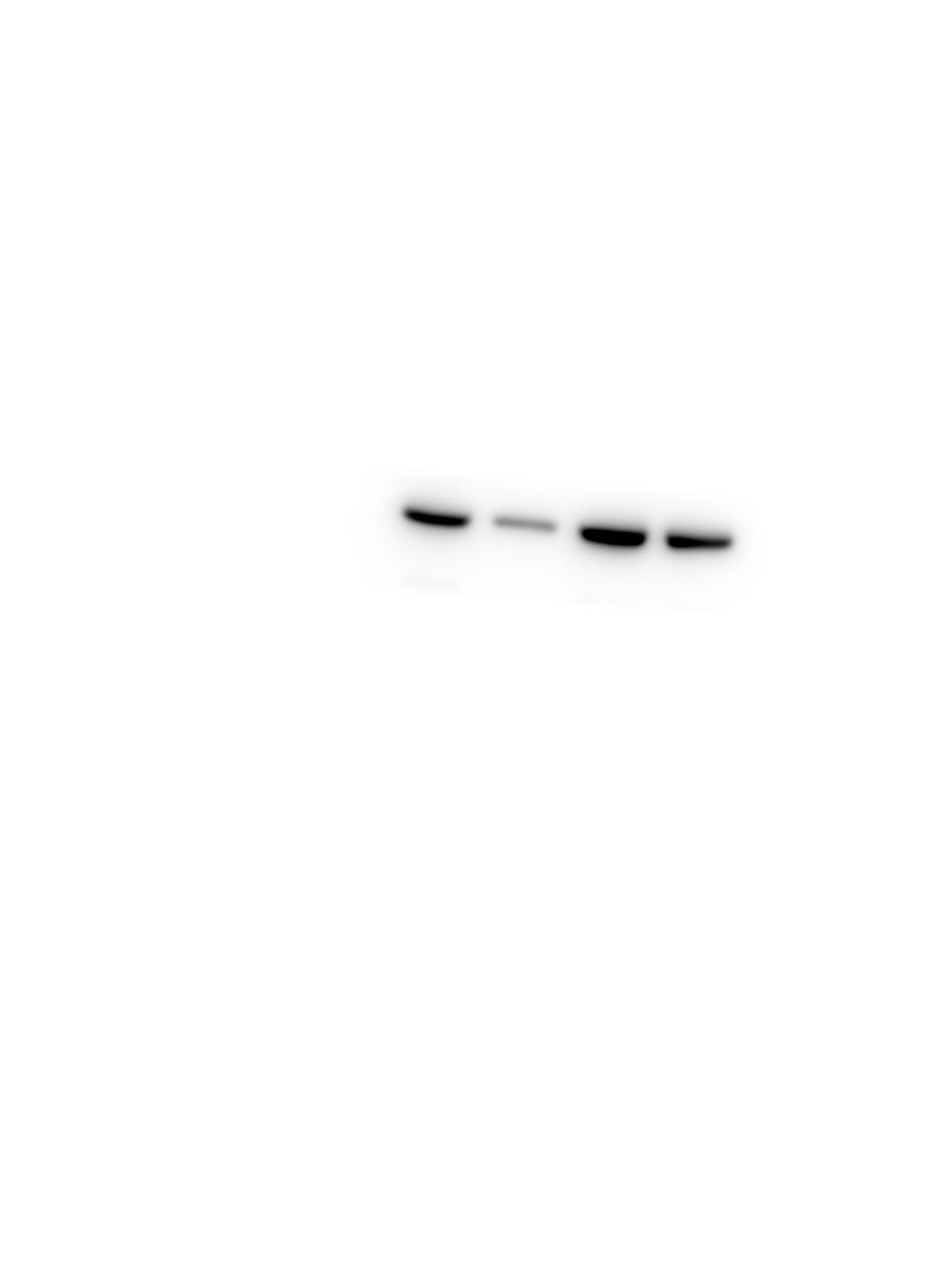

Supplement: Supplementary file 2 [file DataSheet_2.zip › Original Data 2/Figure 4D/A549/p-AKT/p-AKT.jpg]

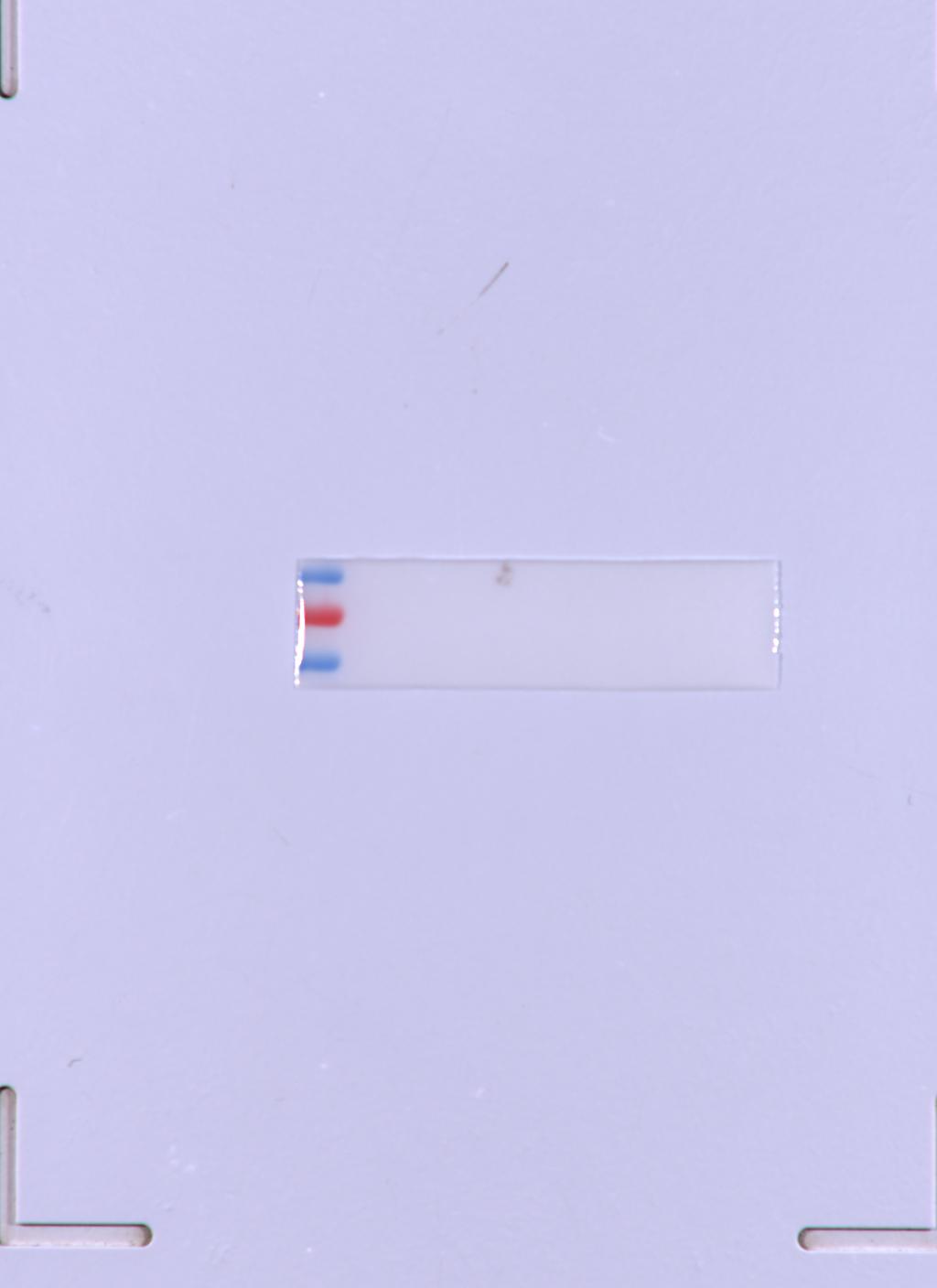

Supplement: Supplementary file 2 [file DataSheet_2.zip › Original Data 2/Figure 4D/NCI-H1299/AKT M/AKT M.jpg]

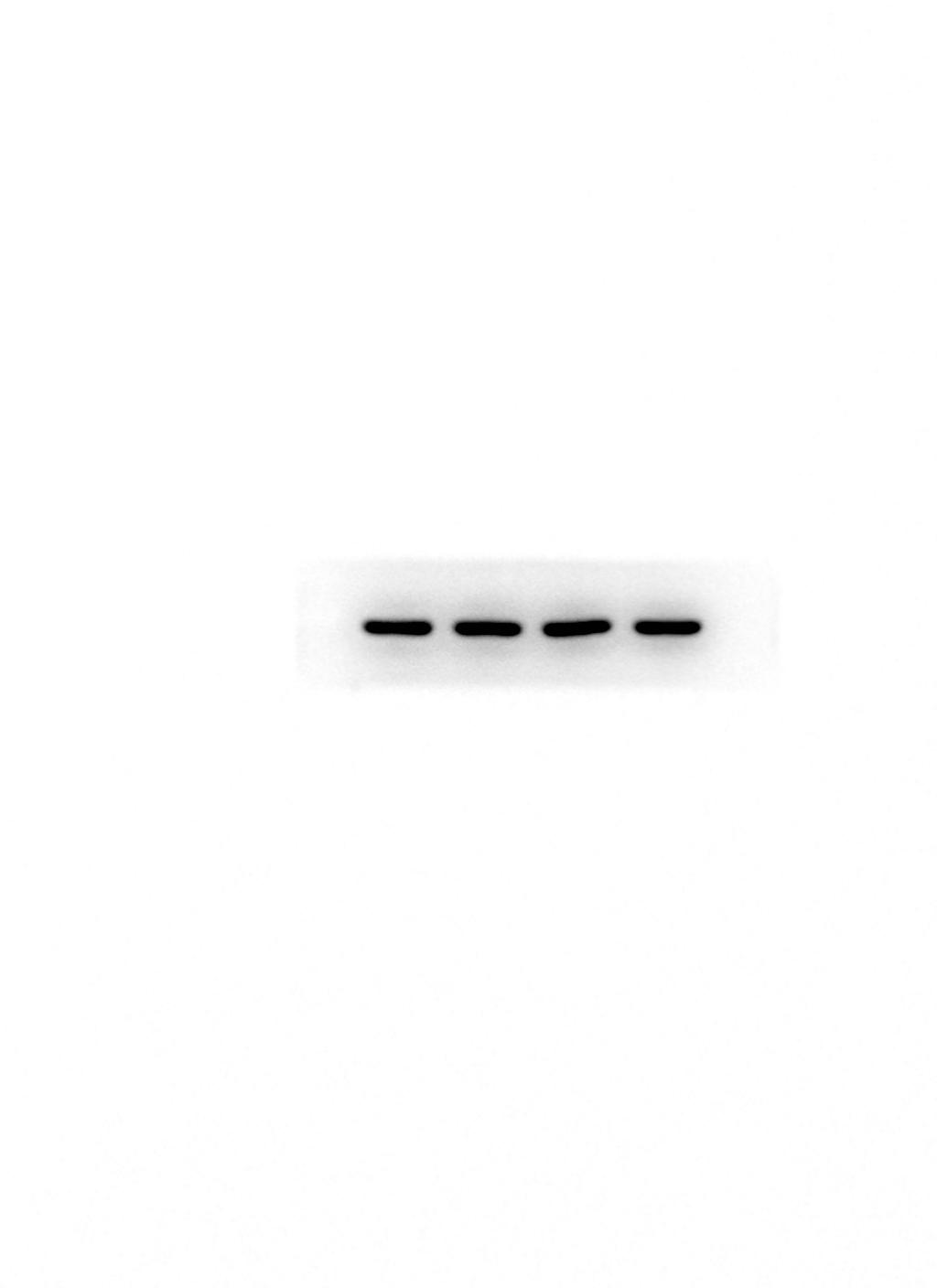

Supplement: Supplementary file 2 [file DataSheet_2.zip › Original Data 2/Figure 4D/NCI-H1299/AKT/AKT.jpg]

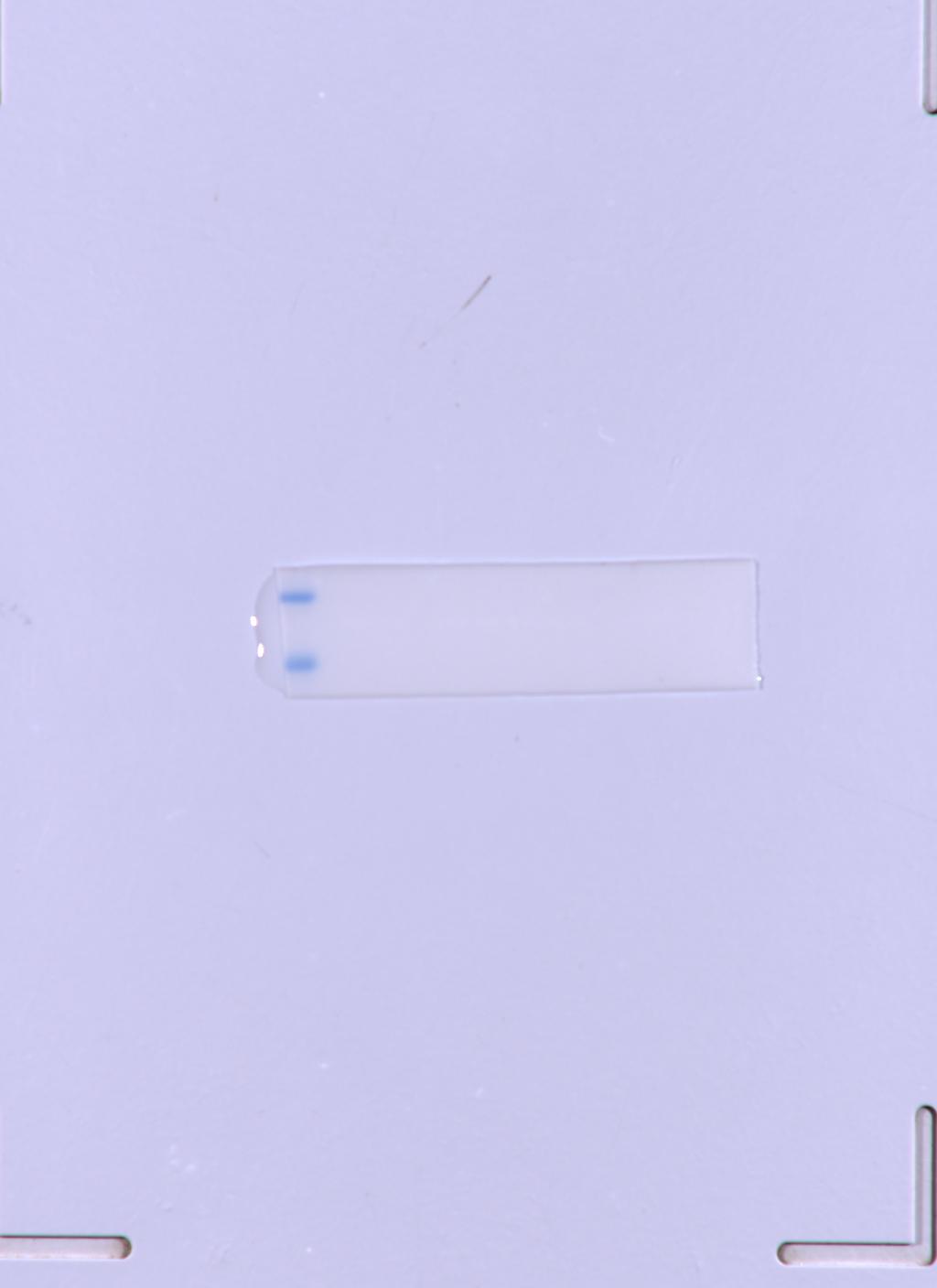

Supplement: Supplementary file 2 [file DataSheet_2.zip › Original Data 2/Figure 4D/NCI-H1299/GAPDH M/GAPDH M.jpg]

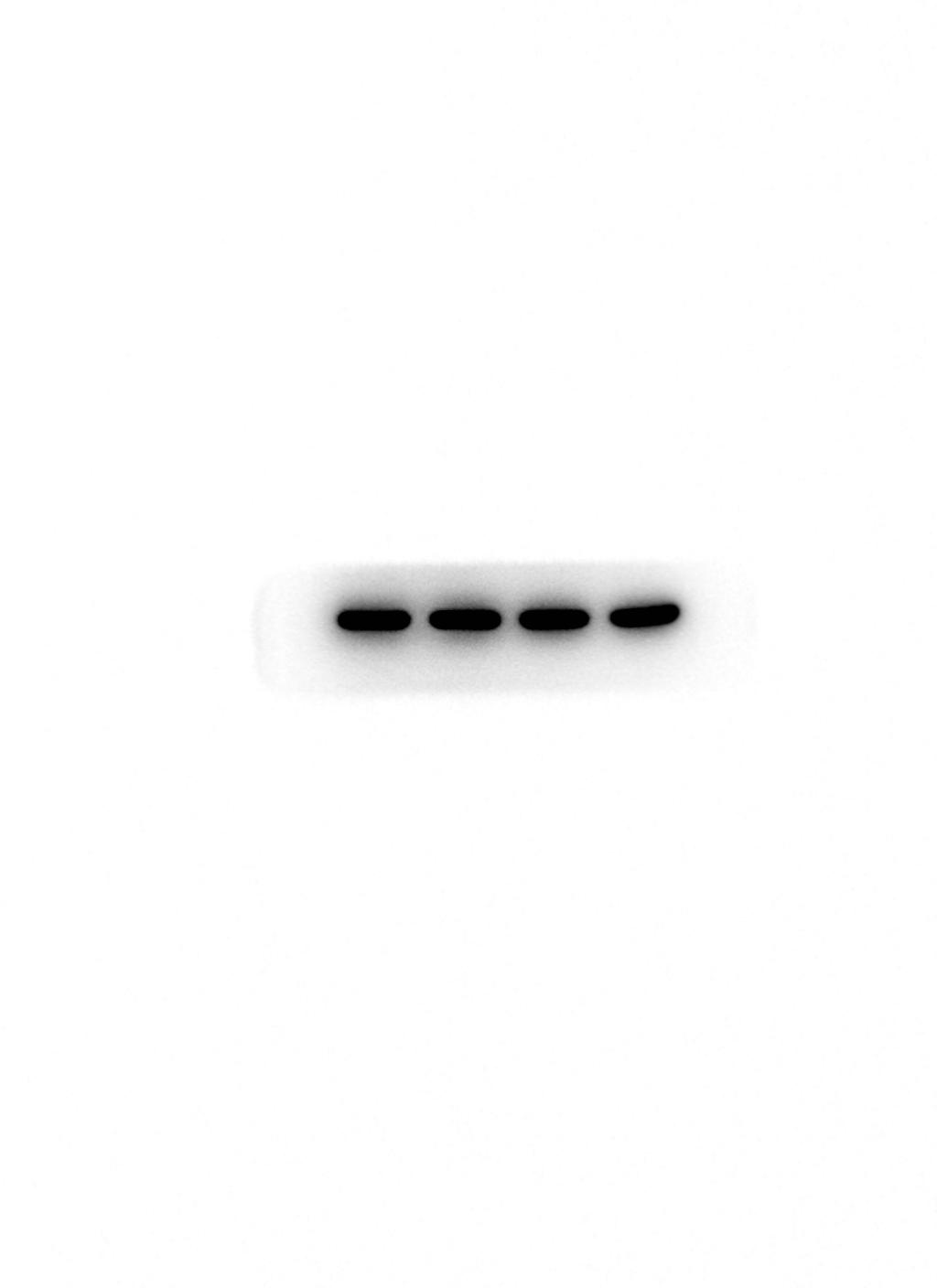

Supplement: Supplementary file 2 [file DataSheet_2.zip › Original Data 2/Figure 4D/NCI-H1299/GAPDH/GAPDH.jpg]

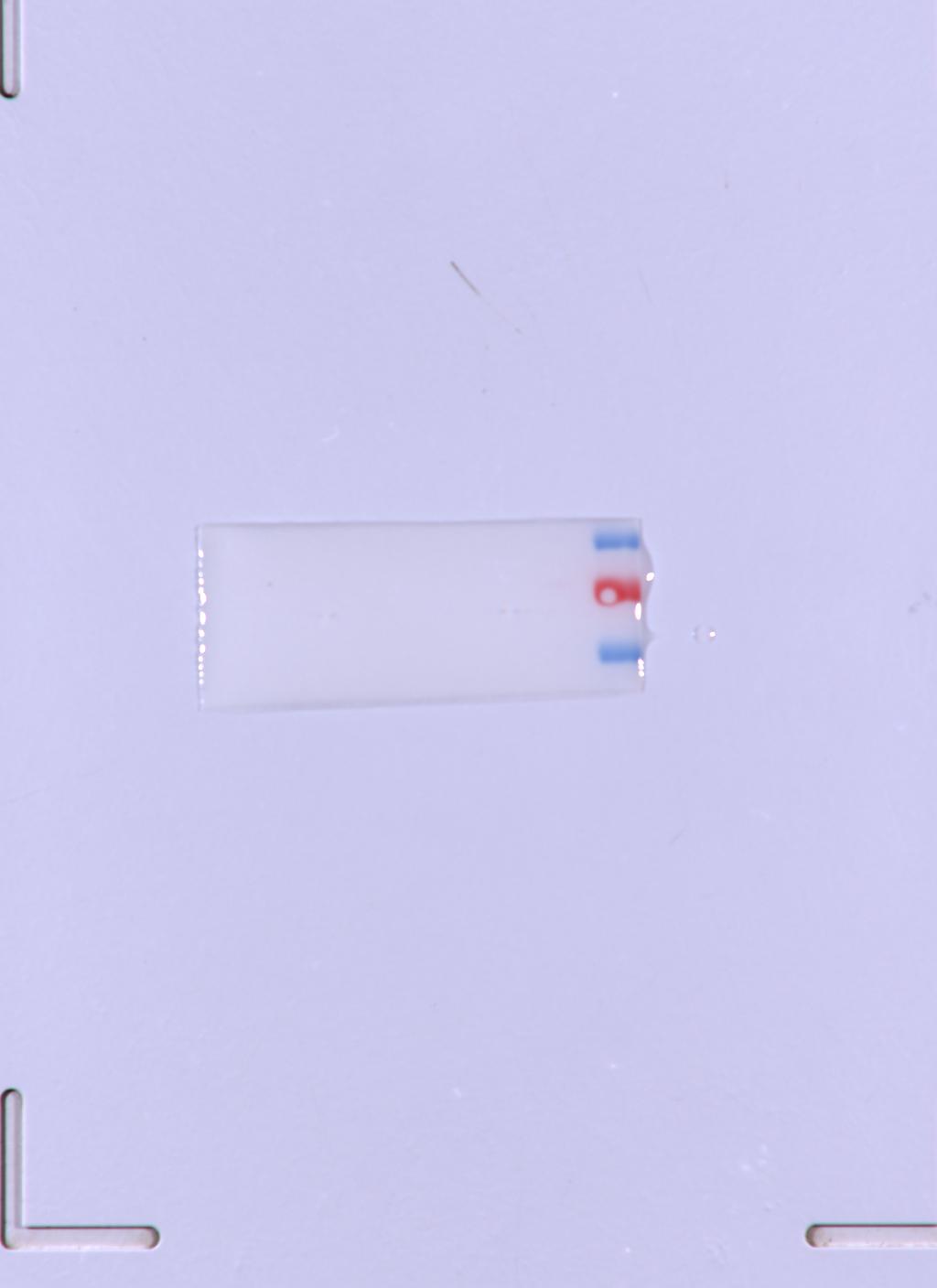

Supplement: Supplementary file 2 [file DataSheet_2.zip › Original Data 2/Figure 4D/NCI-H1299/p-AKT M/p-AKT M.jpg]

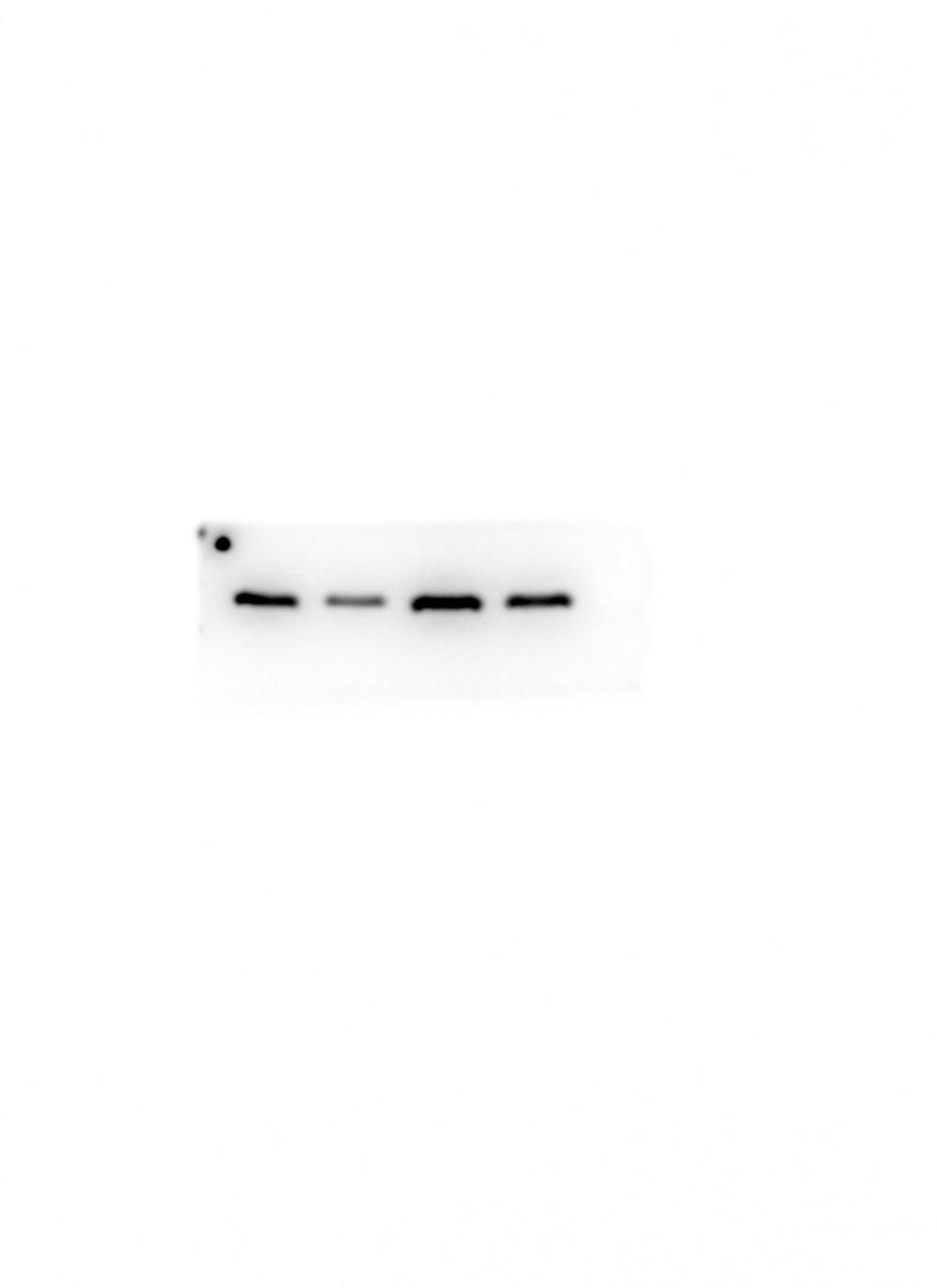

Supplement: Supplementary file 2 [file DataSheet_2.zip › Original Data 2/Figure 4D/NCI-H1299/p-AKT/p-AKT.jpg]

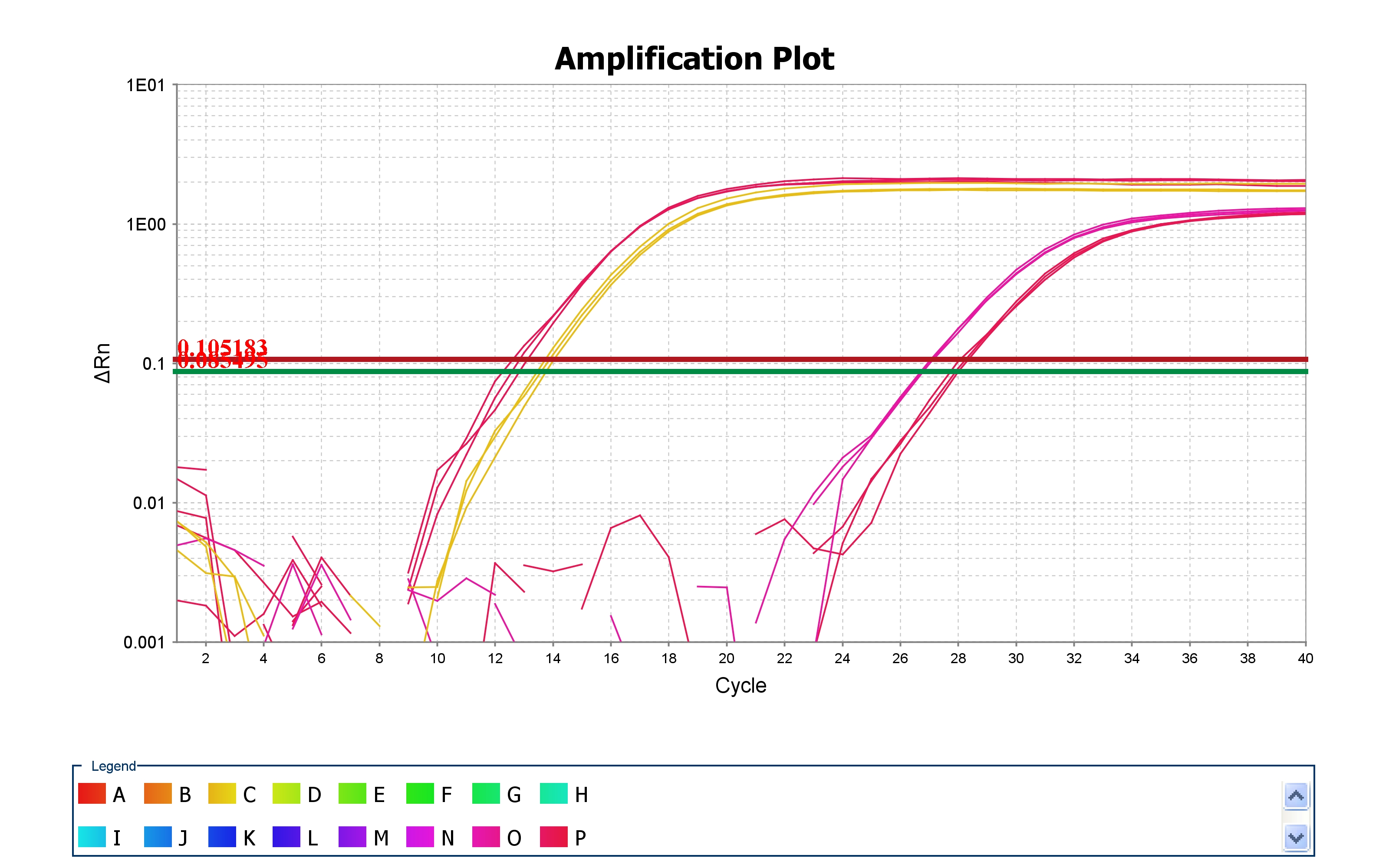

Supplement: Supplementary file 2 [file DataSheet_2.zip › Original Data 2/Figure S1B/A549/Amplification Plot.jpg]

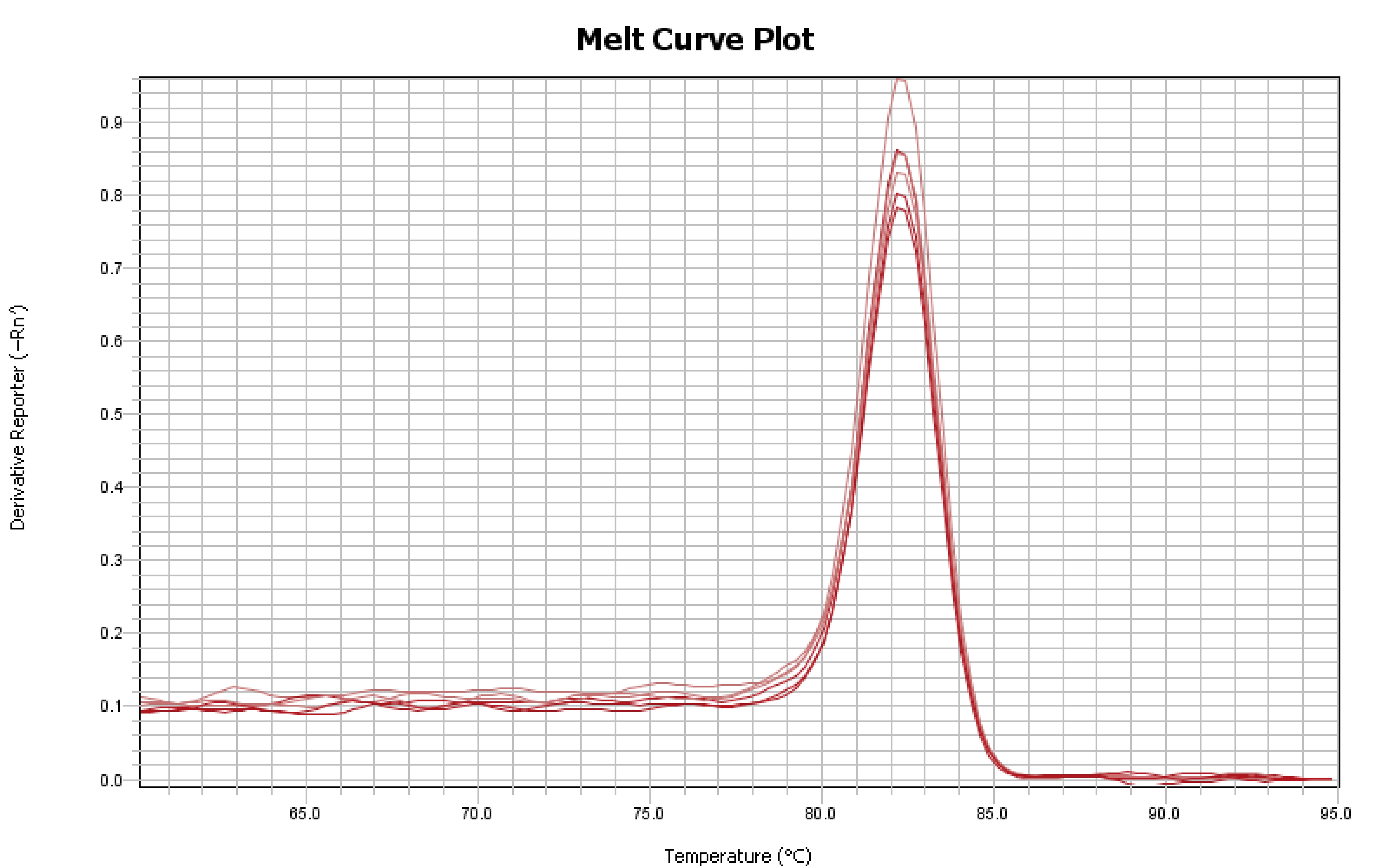

Supplement: Supplementary file 2 [file DataSheet_2.zip › Original Data 2/Figure S1B/A549/Melt Curve Plot H-GAPDH.jpg]

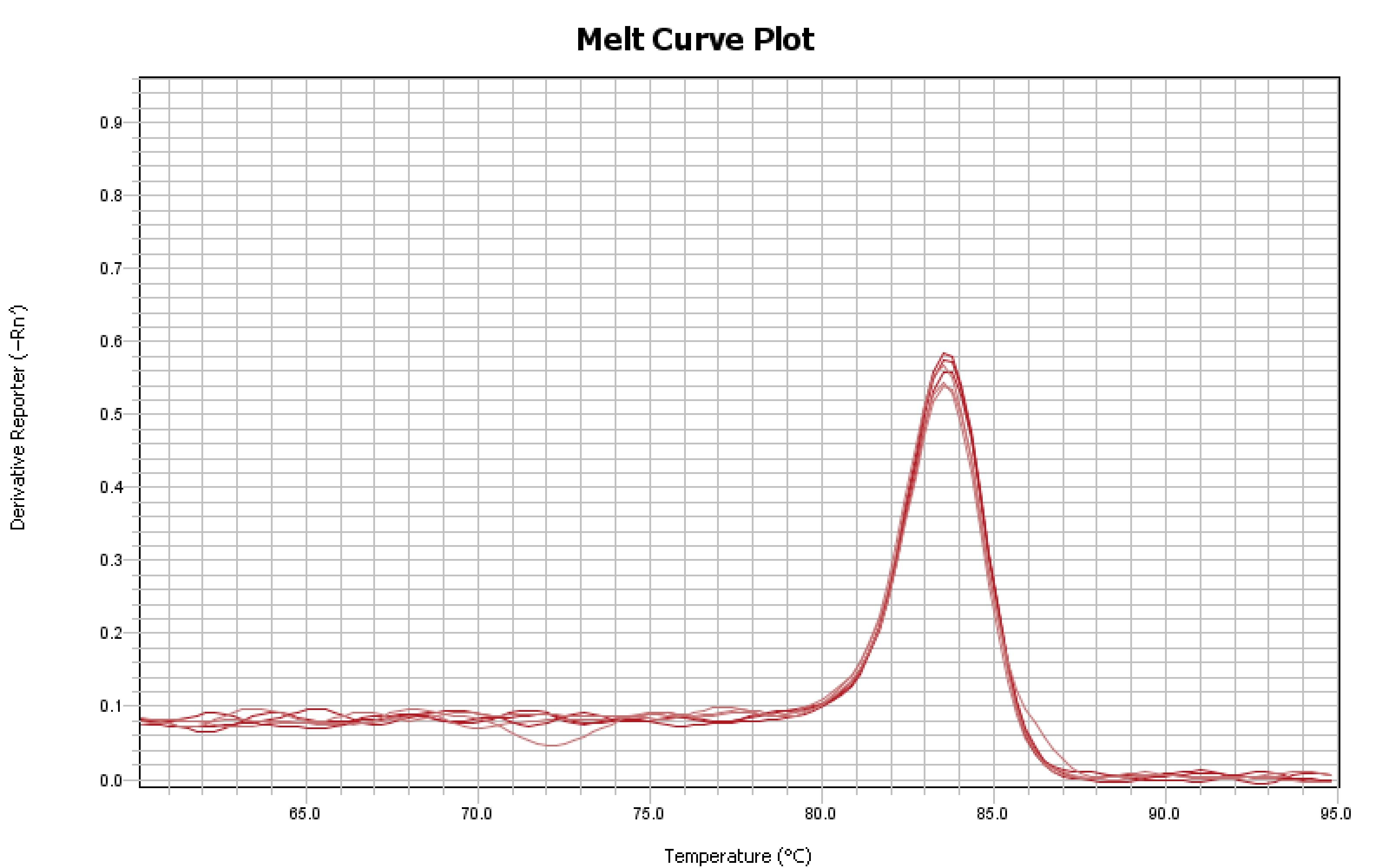

Supplement: Supplementary file 2 [file DataSheet_2.zip › Original Data 2/Figure S1B/A549/Melt Curve Plot H-NLE1.jpg]

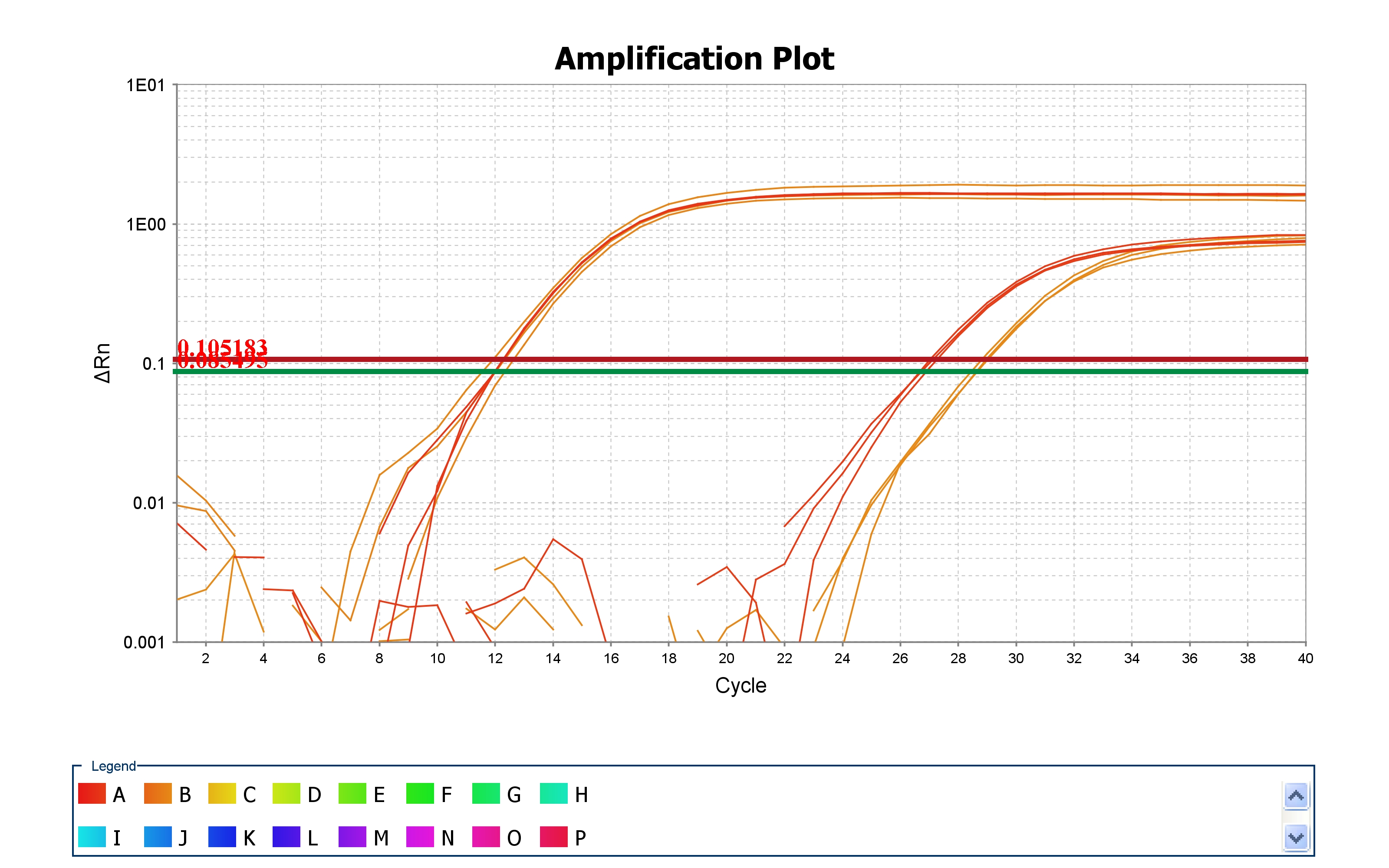

Supplement: Supplementary file 2 [file DataSheet_2.zip › Original Data 2/Figure S1B/NCI-H1299/Amplification Plot.jpg]

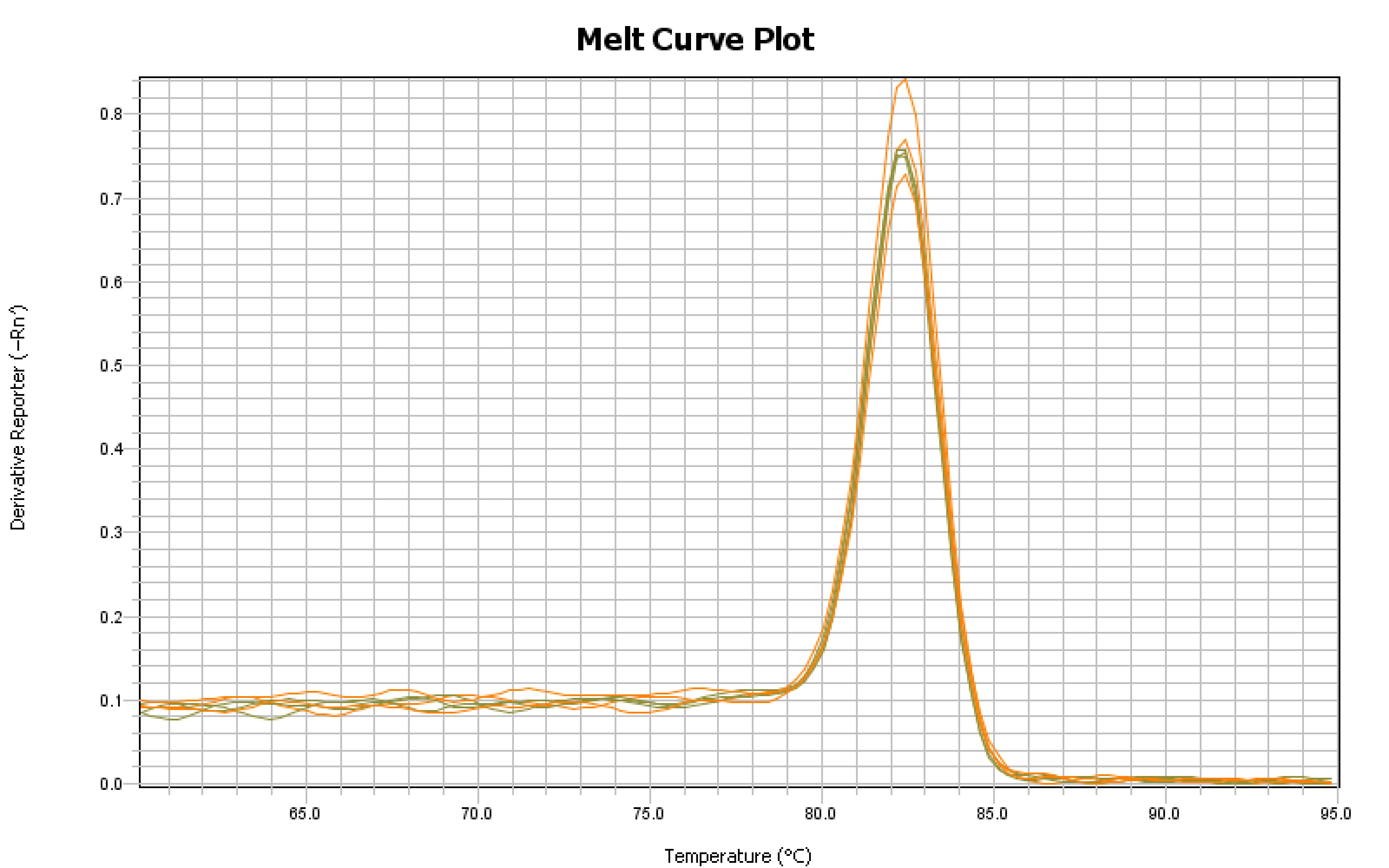

Supplement: Supplementary file 2 [file DataSheet_2.zip › Original Data 2/Figure S1B/NCI-H1299/Melt Curve Plot H-GAPDH.jpg]

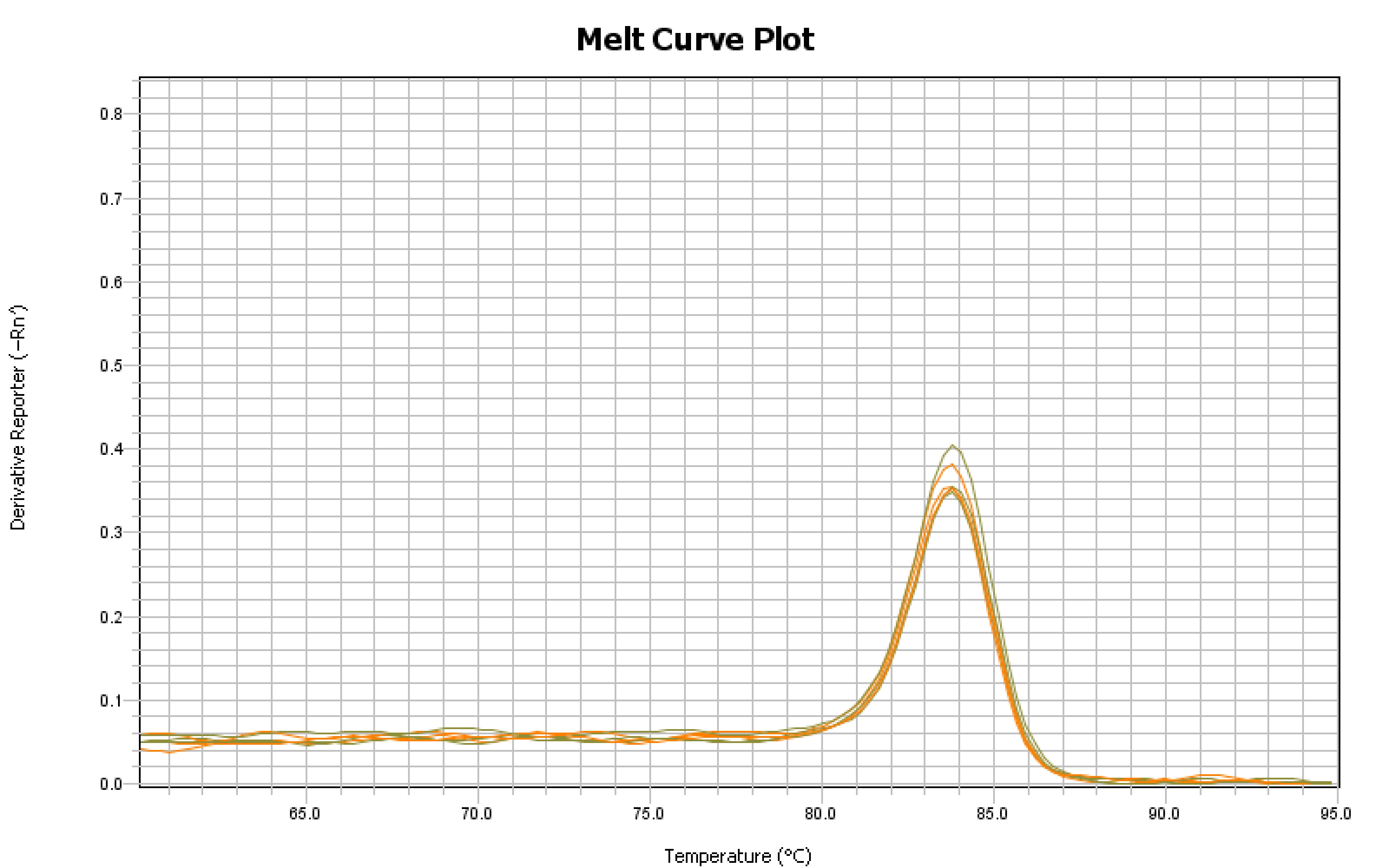

Supplement: Supplementary file 2 [file DataSheet_2.zip › Original Data 2/Figure S1B/NCI-H1299/Melt Curve Plot H-NLE1.jpg]

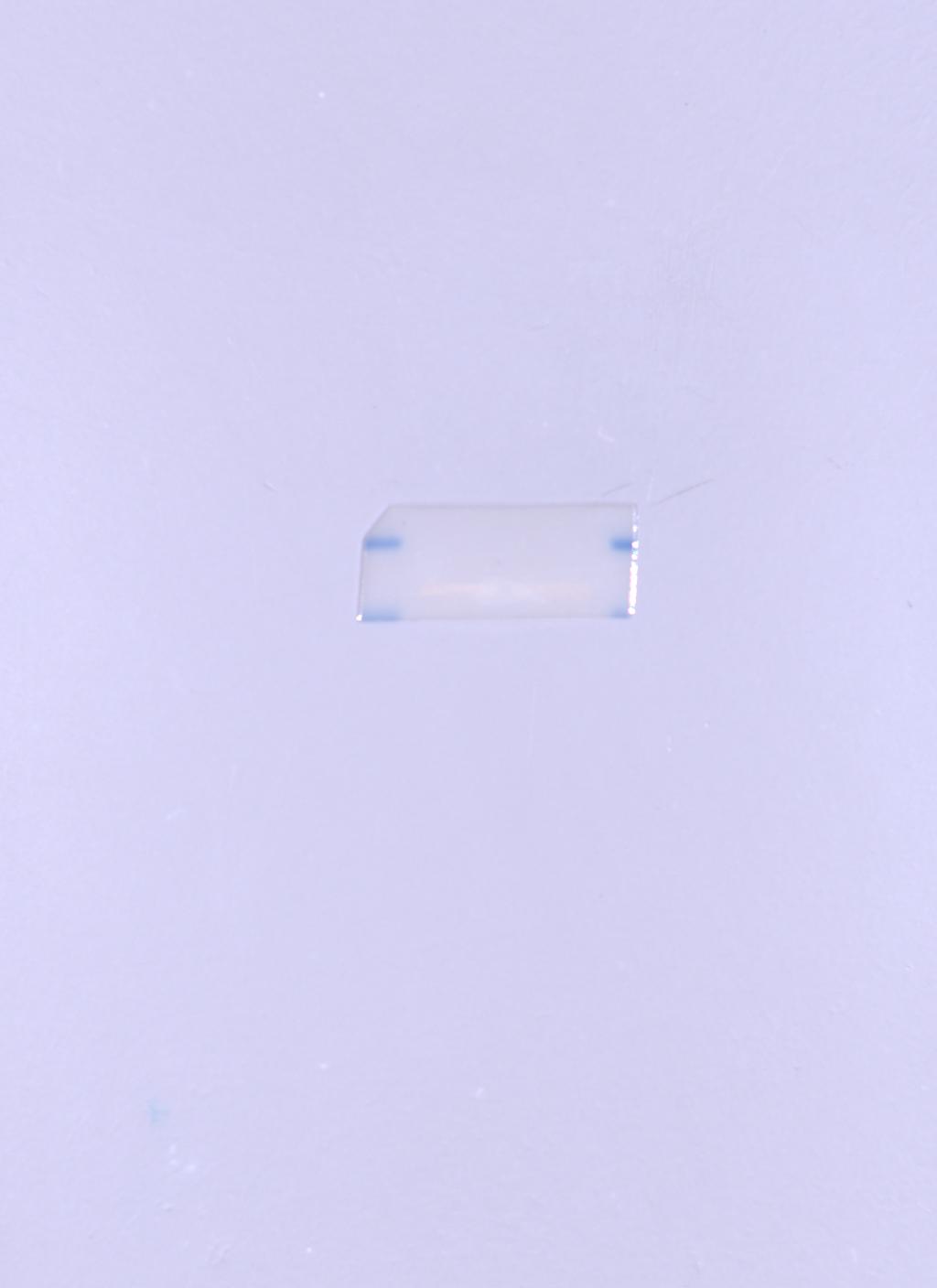

Supplement: Supplementary file 2 [file DataSheet_2.zip › Original Data 2/Figure S1C/A549/A549 GAPDH M/A549 GAPDH M.jpg]

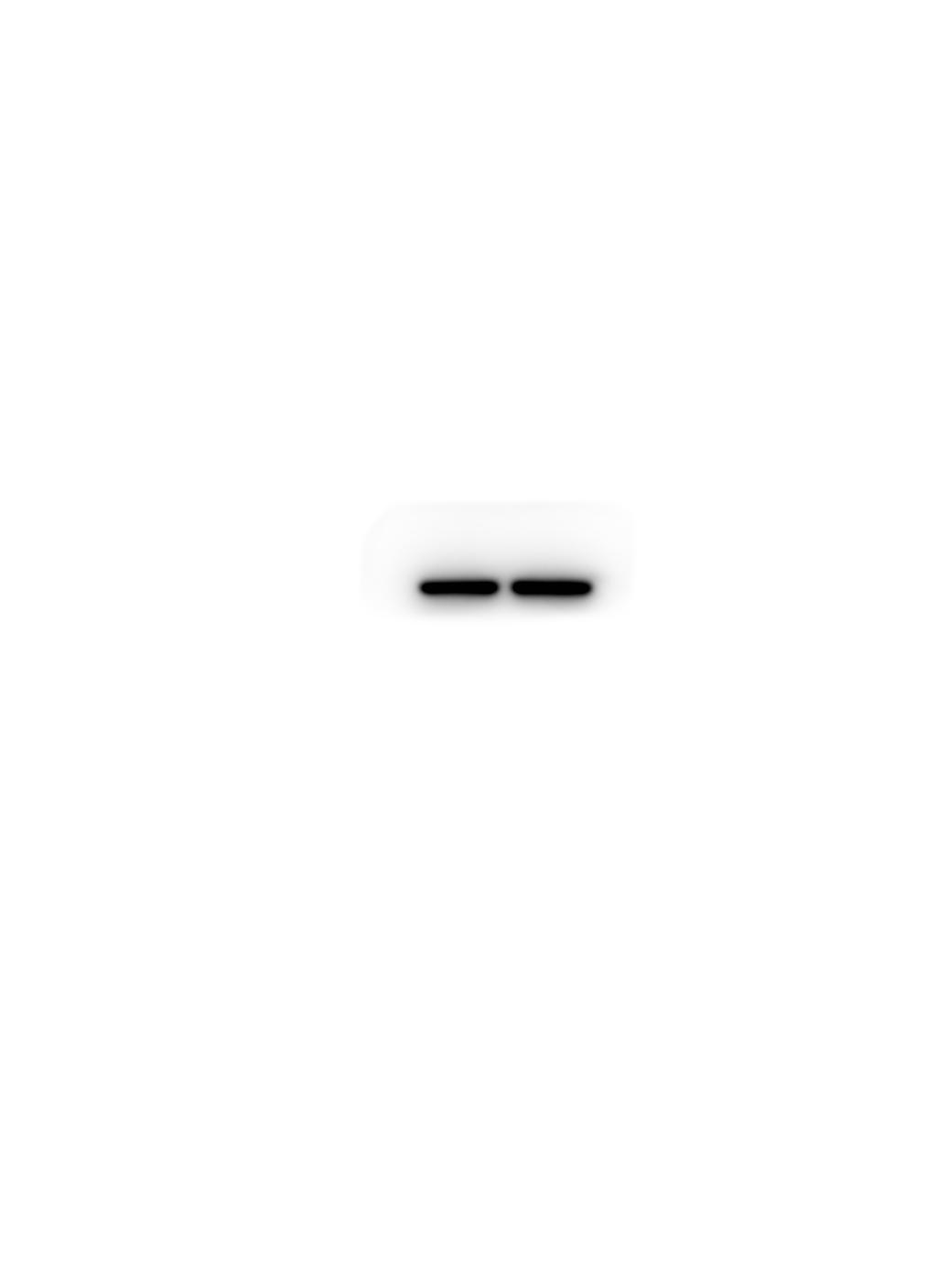

Supplement: Supplementary file 2 [file DataSheet_2.zip › Original Data 2/Figure S1C/A549/A549 GAPDH/A549 GAPDH.jpg]

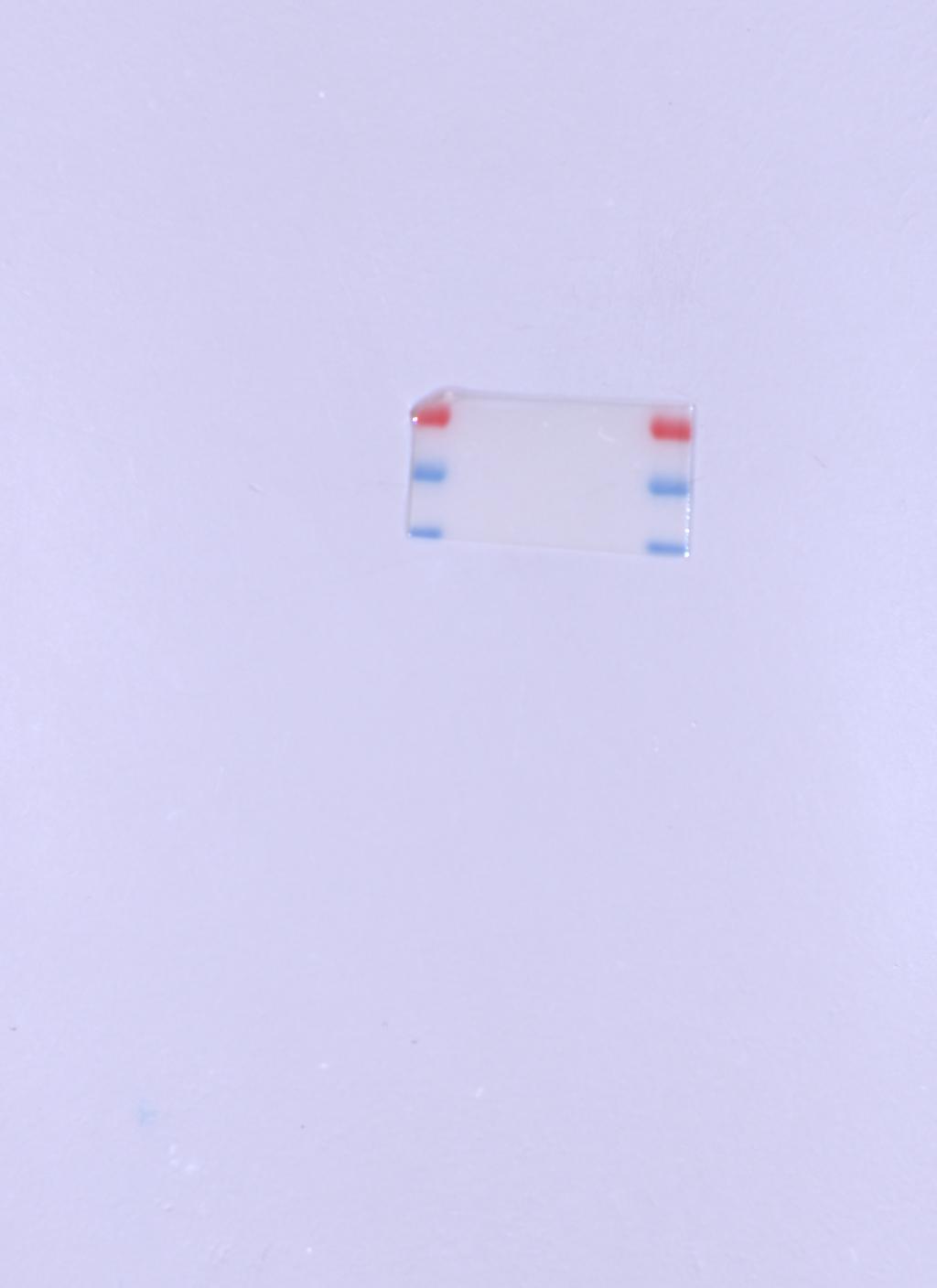

Supplement: Supplementary file 2 [file DataSheet_2.zip › Original Data 2/Figure S1C/A549/A549 NLE1 M/A549 NLE1 M.jpg]

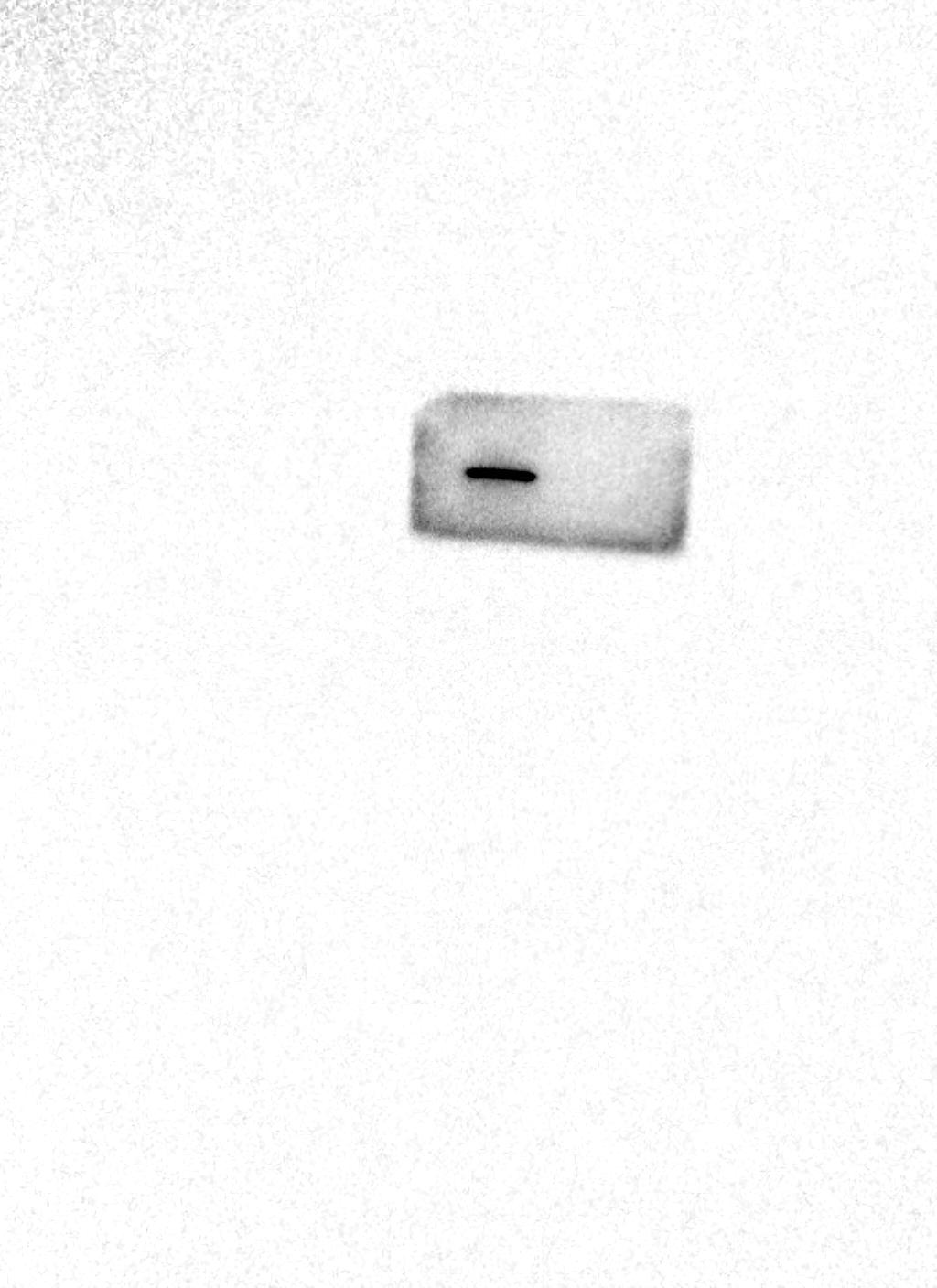

Supplement: Supplementary file 2 [file DataSheet_2.zip › Original Data 2/Figure S1C/A549/A549 NLE1/A549 NLE1.jpg]

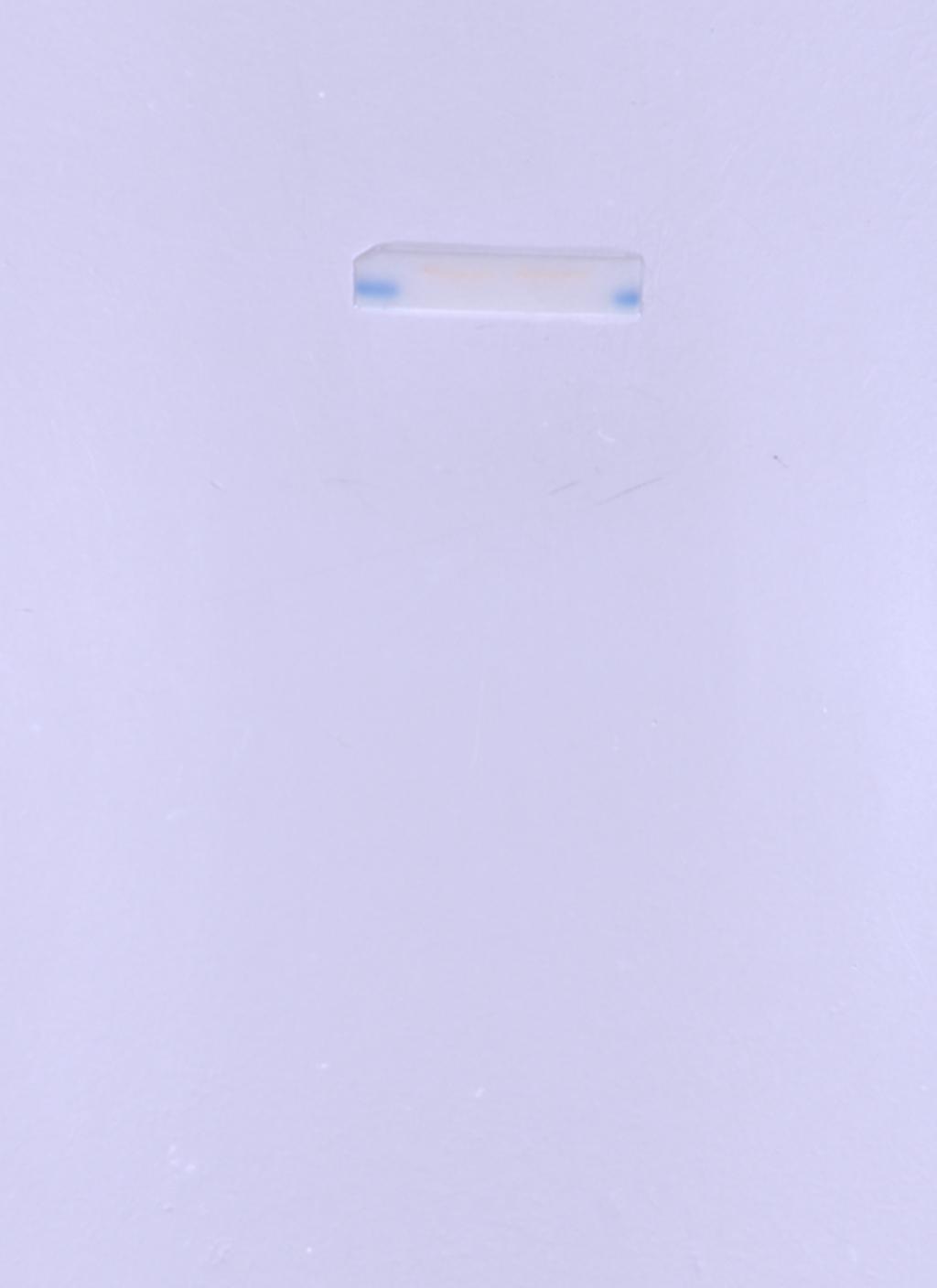

Supplement: Supplementary file 2 [file DataSheet_2.zip › Original Data 2/Figure S1C/NCI-H1299/NCI-H1299 GAPDH M/NCI-H1299 GAPDH M.jpg]

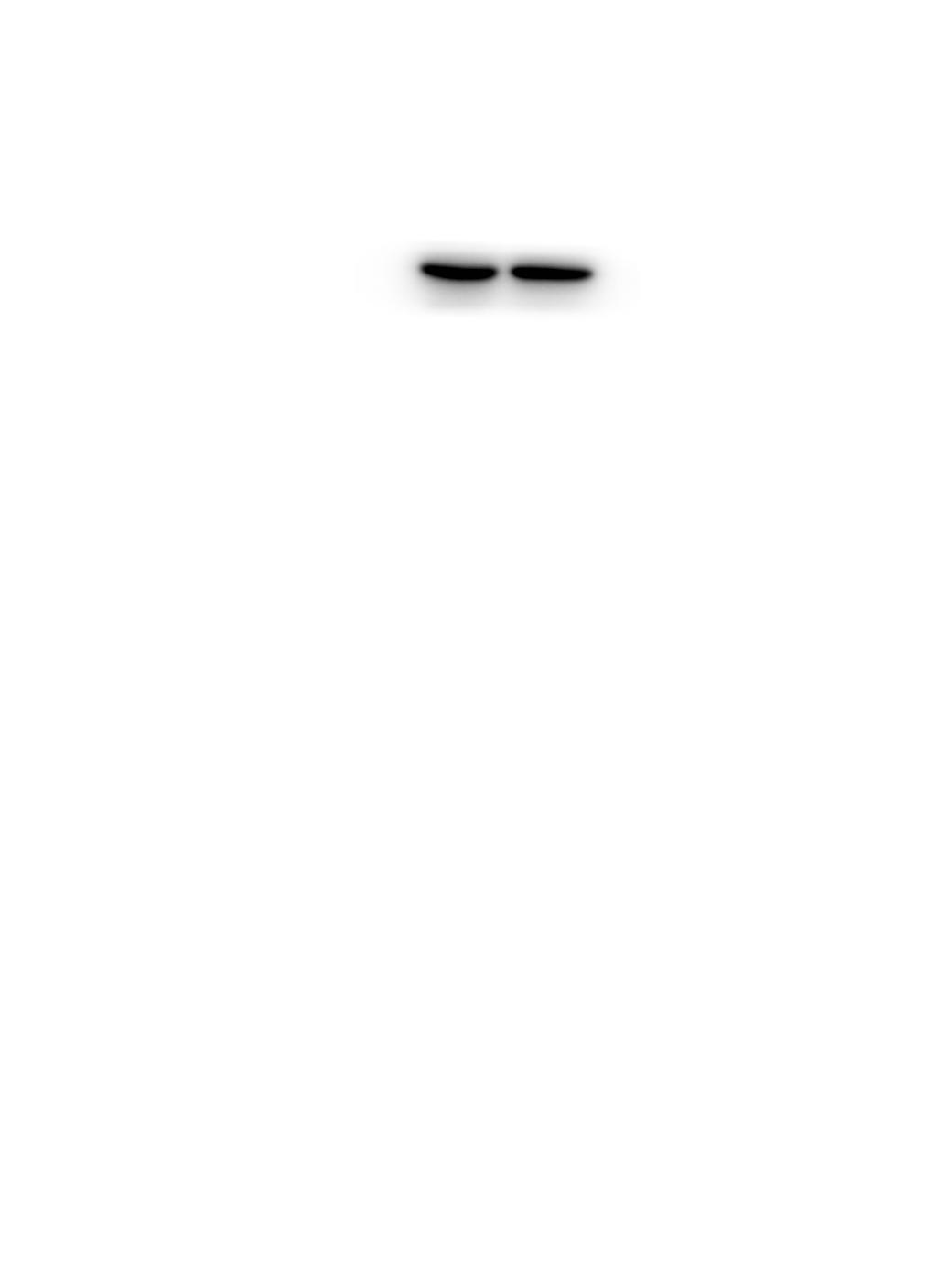

Supplement: Supplementary file 2 [file DataSheet_2.zip › Original Data 2/Figure S1C/NCI-H1299/NCI-H1299 GAPDH/NCI-H1299 GAPDH.jpg]

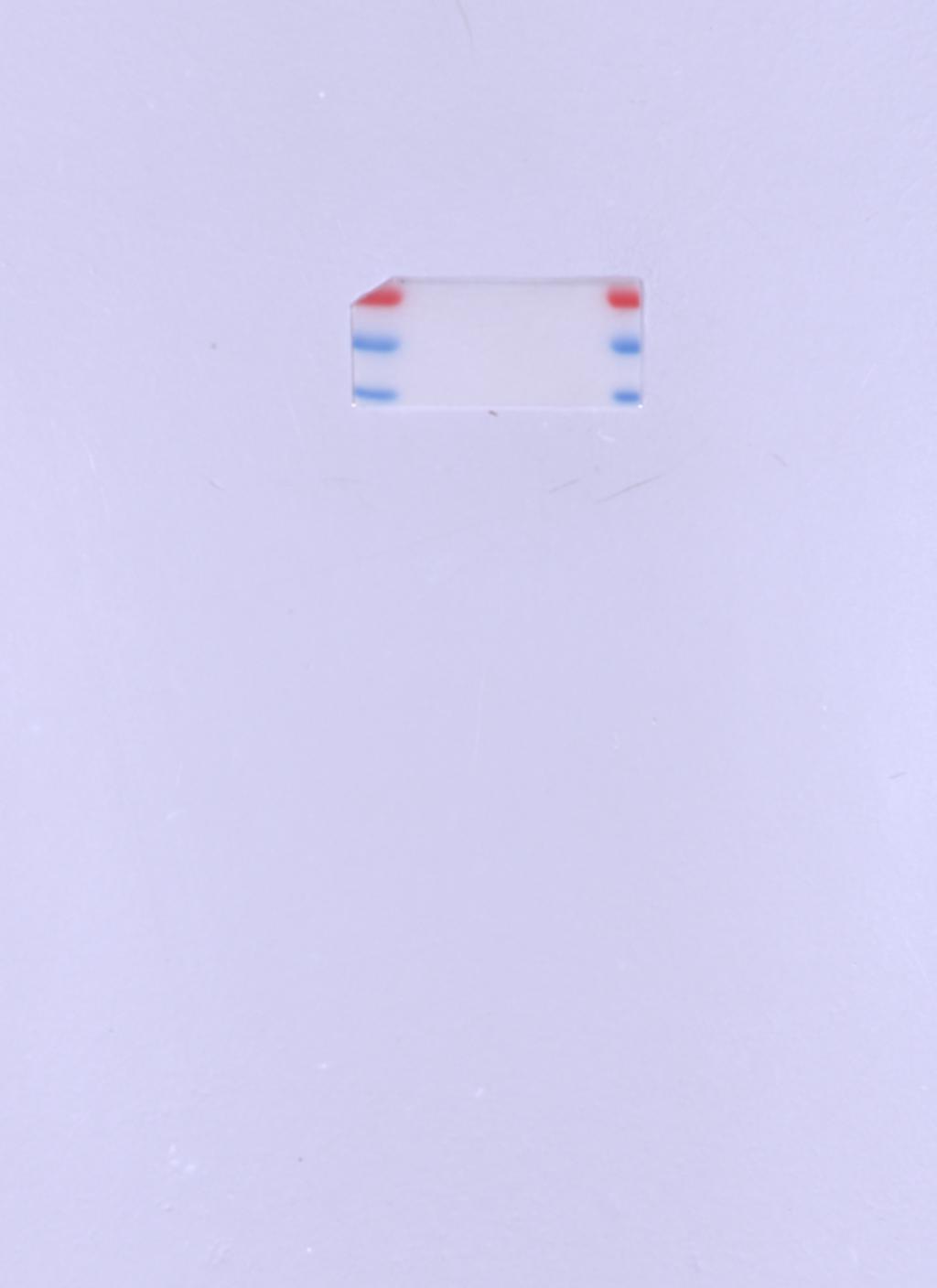

Supplement: Supplementary file 2 [file DataSheet_2.zip › Original Data 2/Figure S1C/NCI-H1299/NCI-H1299 NLE1 M/NCI-H1299 NLE1 M.jpg]

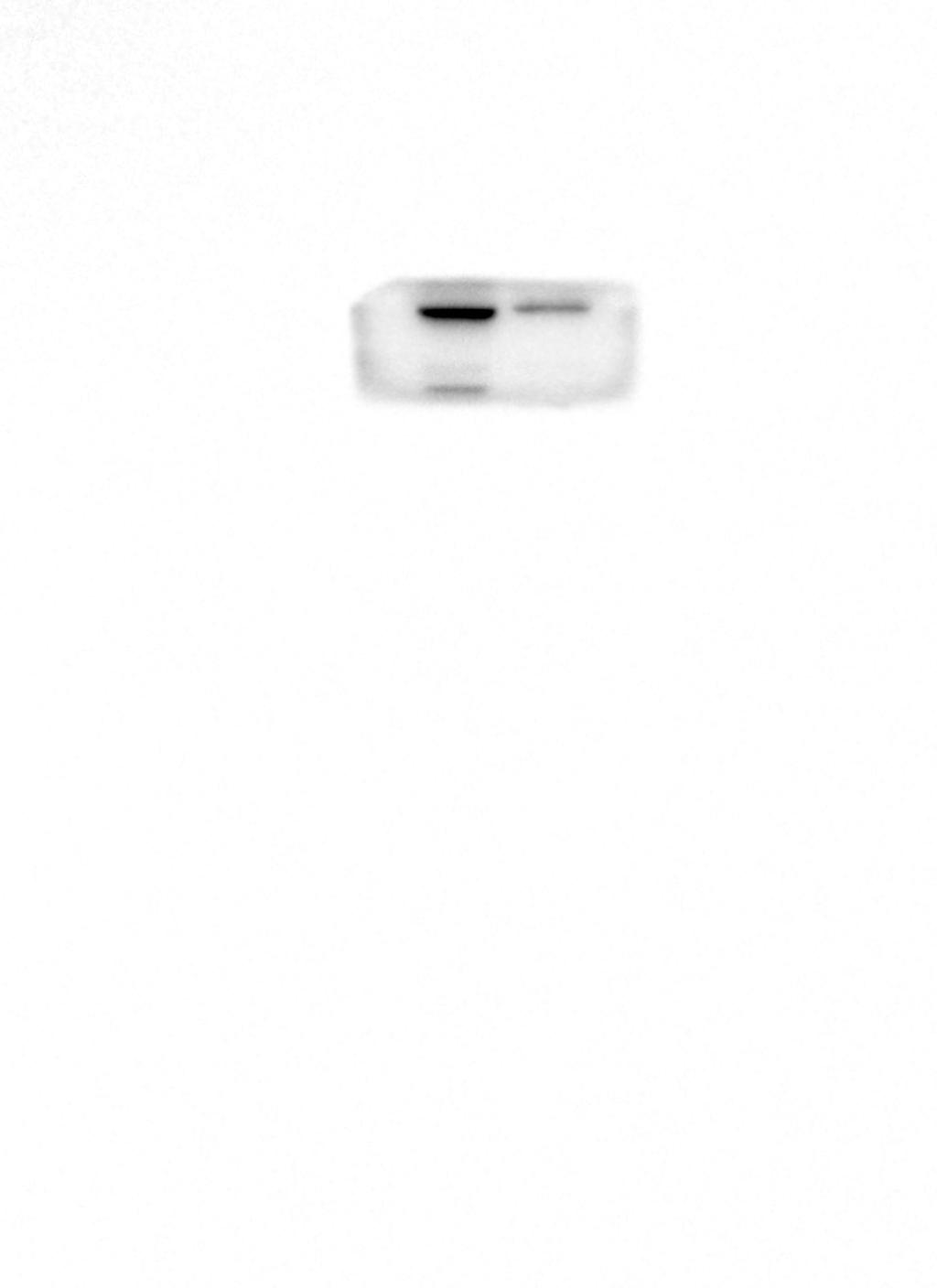

Supplement: Supplementary file 2 [file DataSheet_2.zip › Original Data 2/Figure S1C/NCI-H1299/NCI-H1299 NLE1/NCI-H1299 NLE1.jpg]

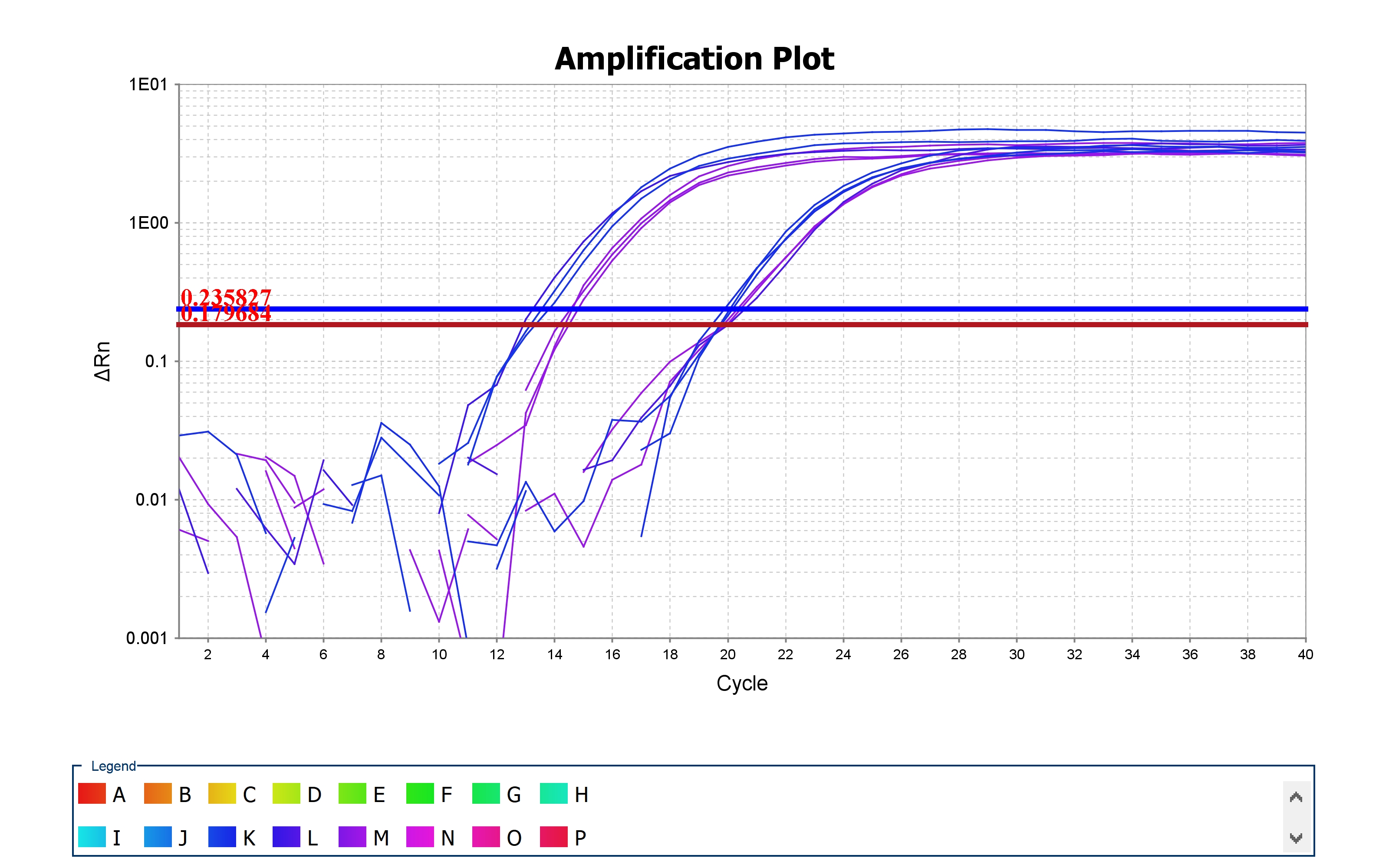

Supplement: Supplementary file 2 [file DataSheet_2.zip › Original Data 2/Figure S3B/CDK1/Amplification Plot.jpg]

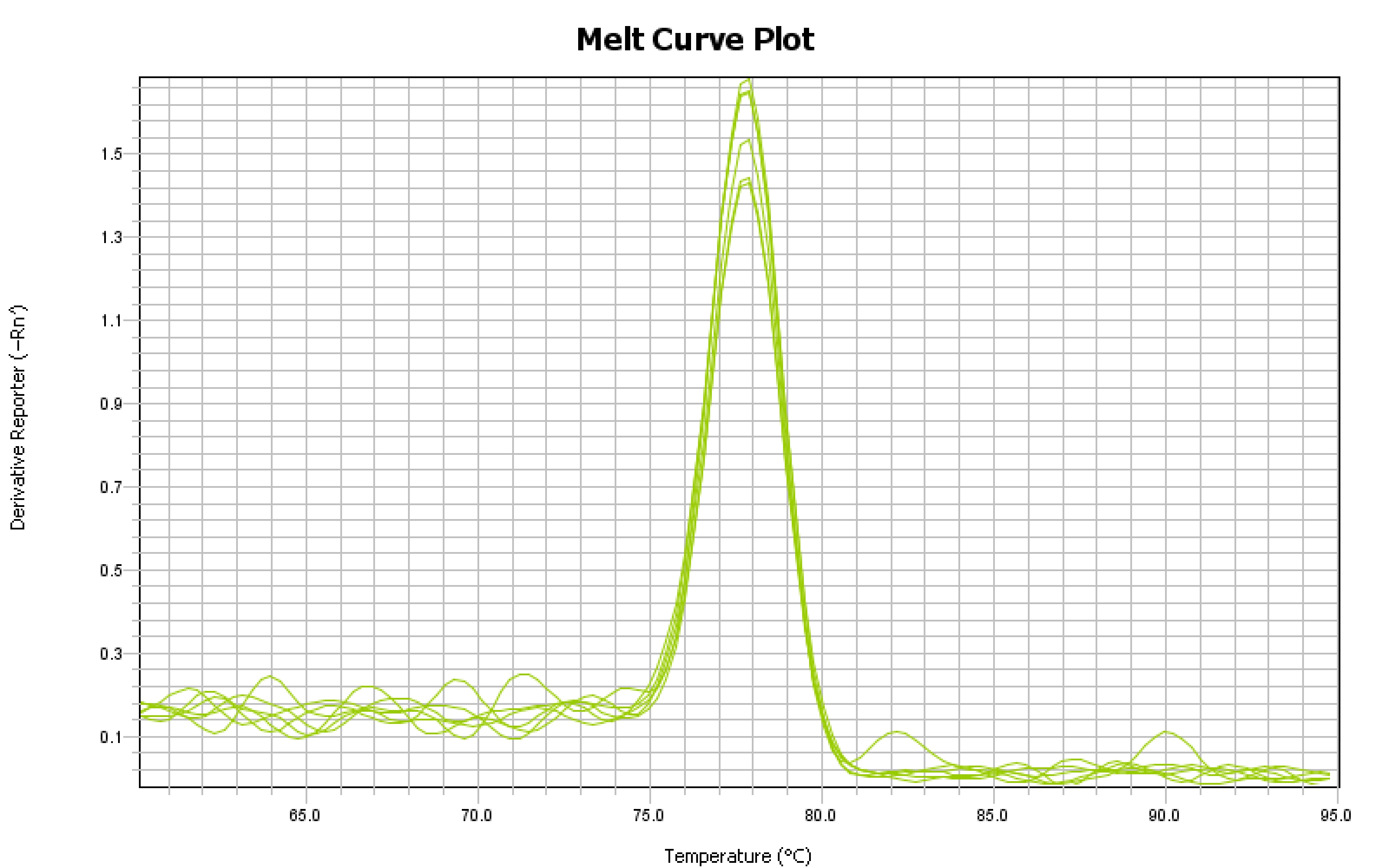

Supplement: Supplementary file 2 [file DataSheet_2.zip › Original Data 2/Figure S3B/CDK1/Melt Curve Plot H-CDK1.jpg]

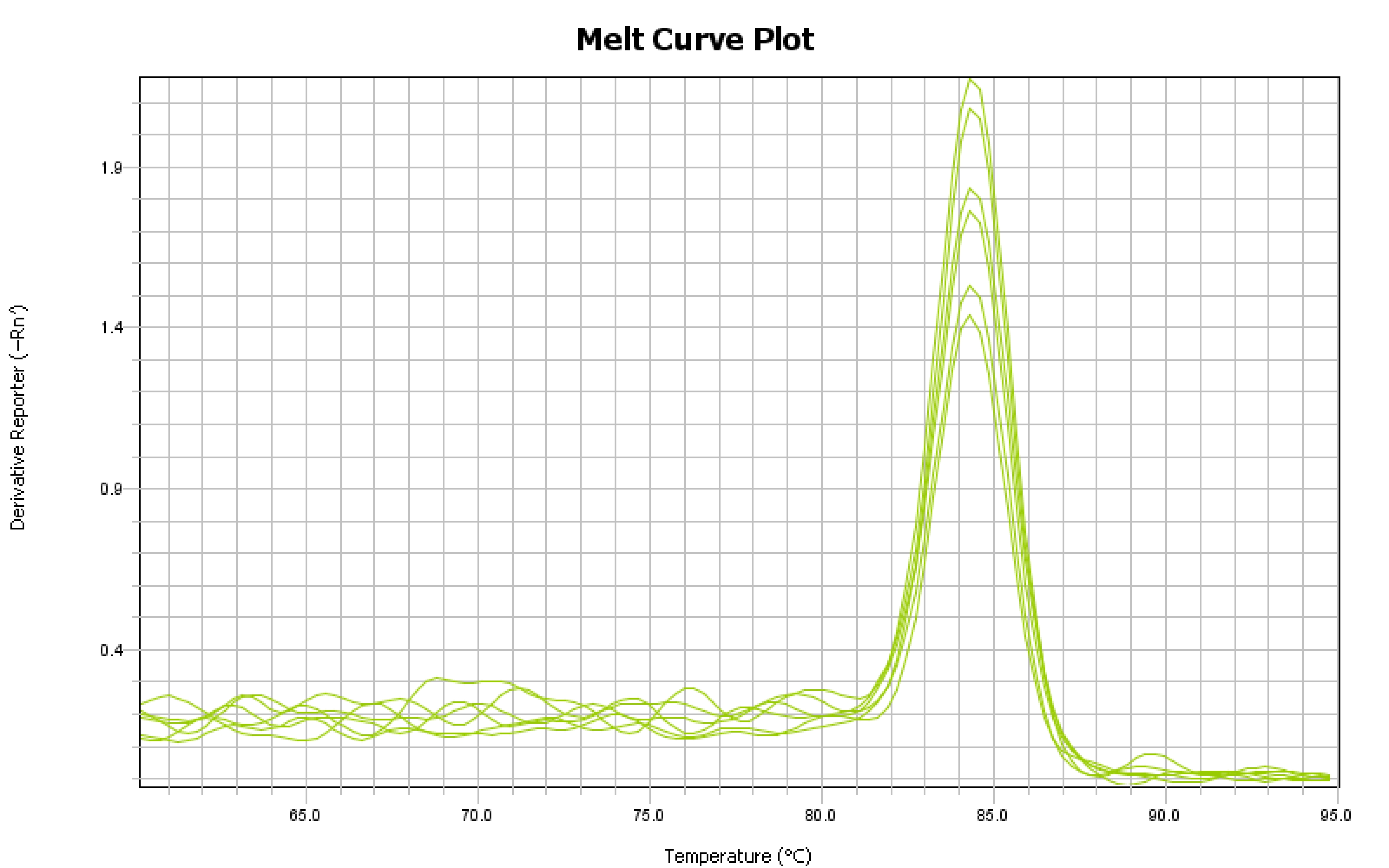

Supplement: Supplementary file 2 [file DataSheet_2.zip › Original Data 2/Figure S3B/CDK1/Melt Curve Plot H-GAPDH.jpg]

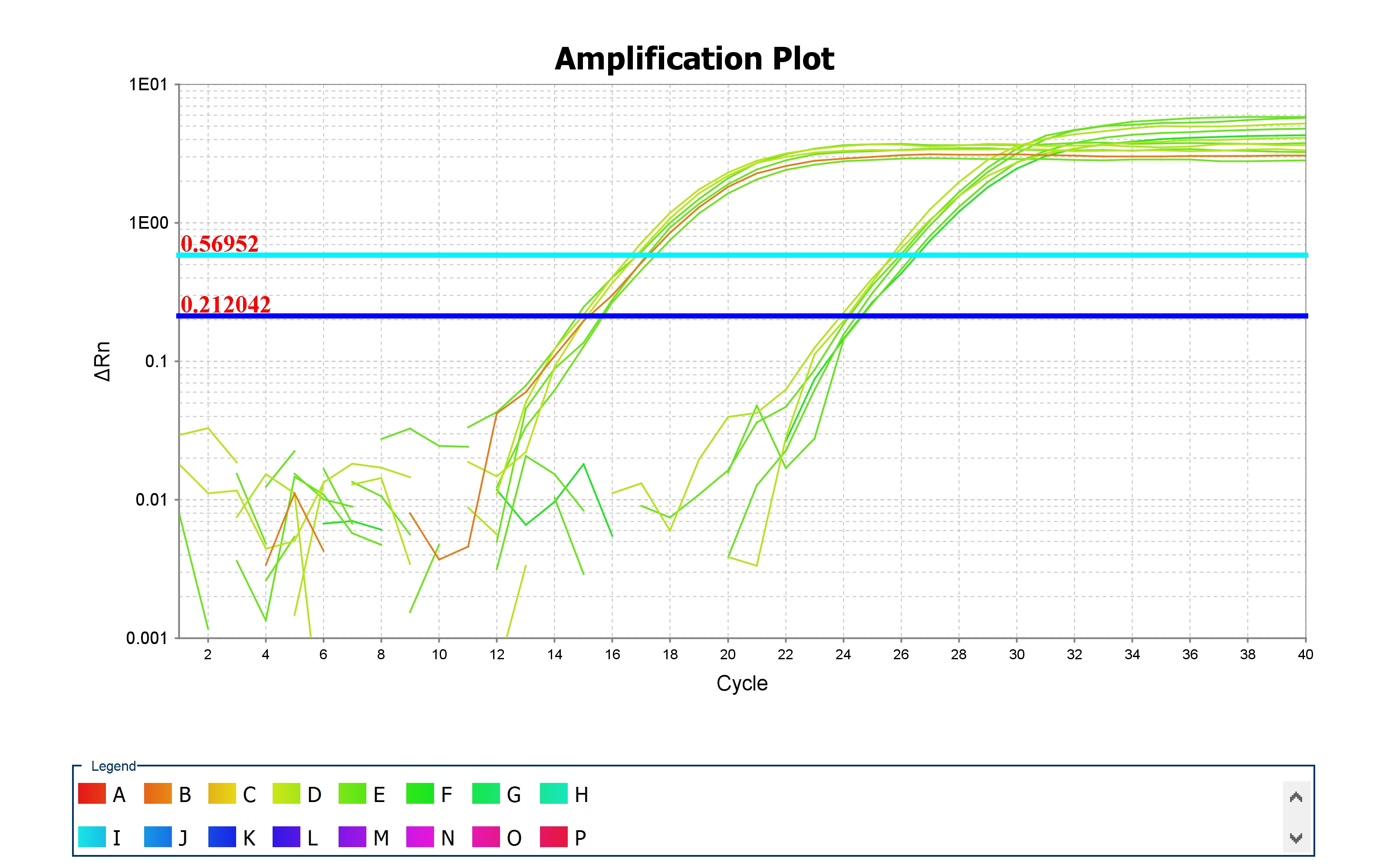

Supplement: Supplementary file 2 [file DataSheet_2.zip › Original Data 2/Figure S3B/shNLE1+CDK1/CDK1/Amplification Plot.jpg]

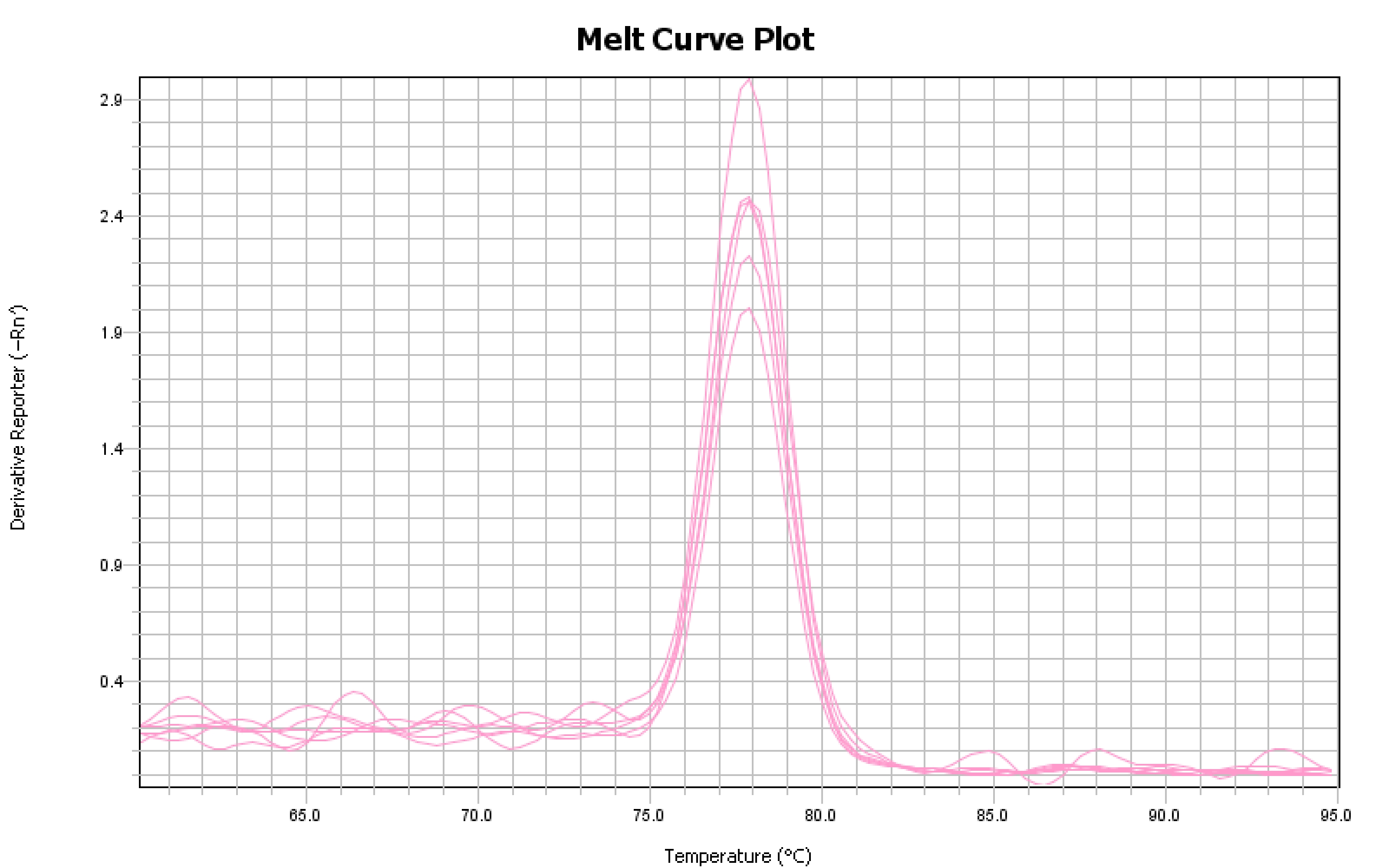

Supplement: Supplementary file 2 [file DataSheet_2.zip › Original Data 2/Figure S3B/shNLE1+CDK1/CDK1/Melt Curve Plot H-CDK1.jpg]

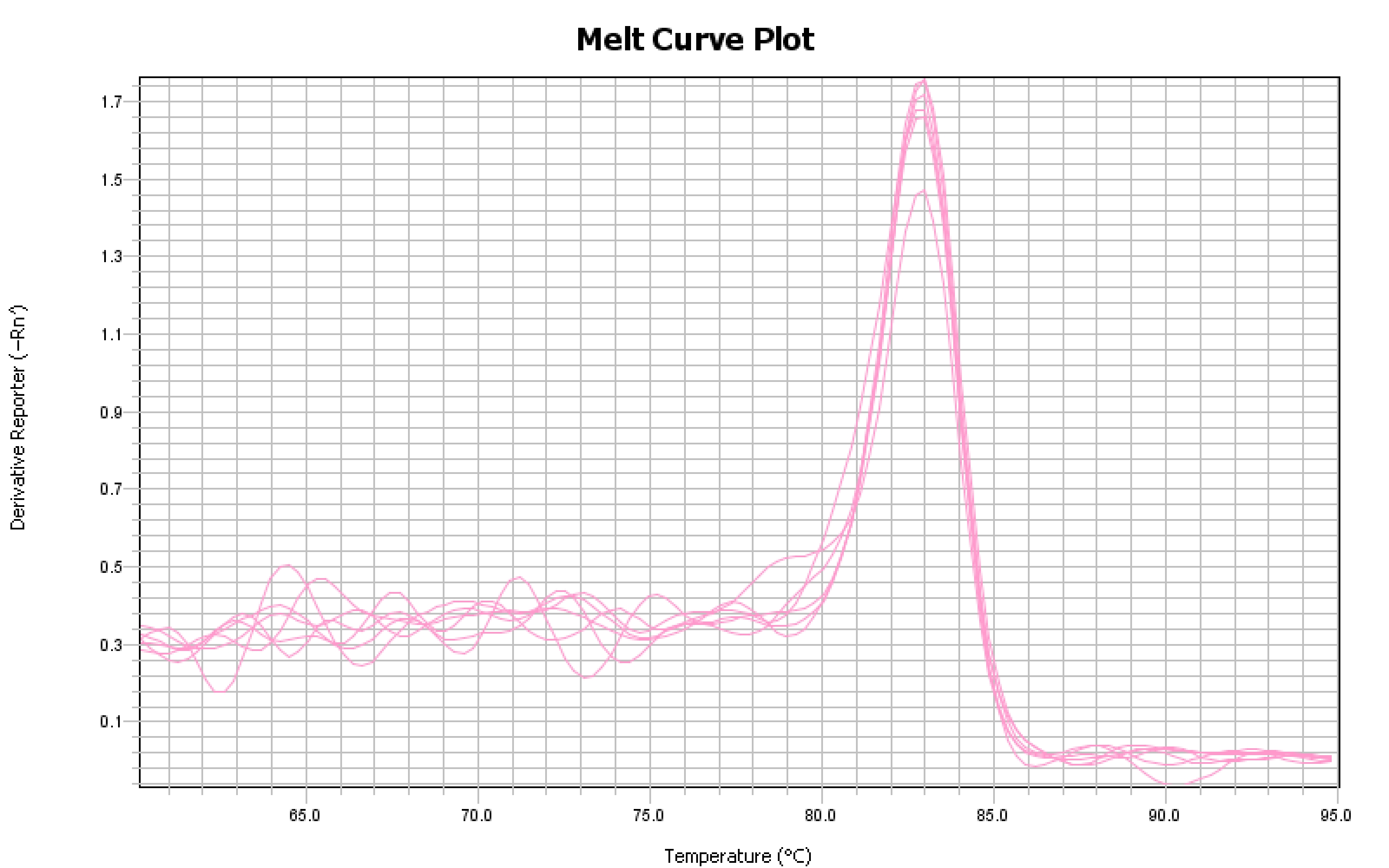

Supplement: Supplementary file 2 [file DataSheet_2.zip › Original Data 2/Figure S3B/shNLE1+CDK1/CDK1/Melt Curve Plot H-GAPDH.jpg]

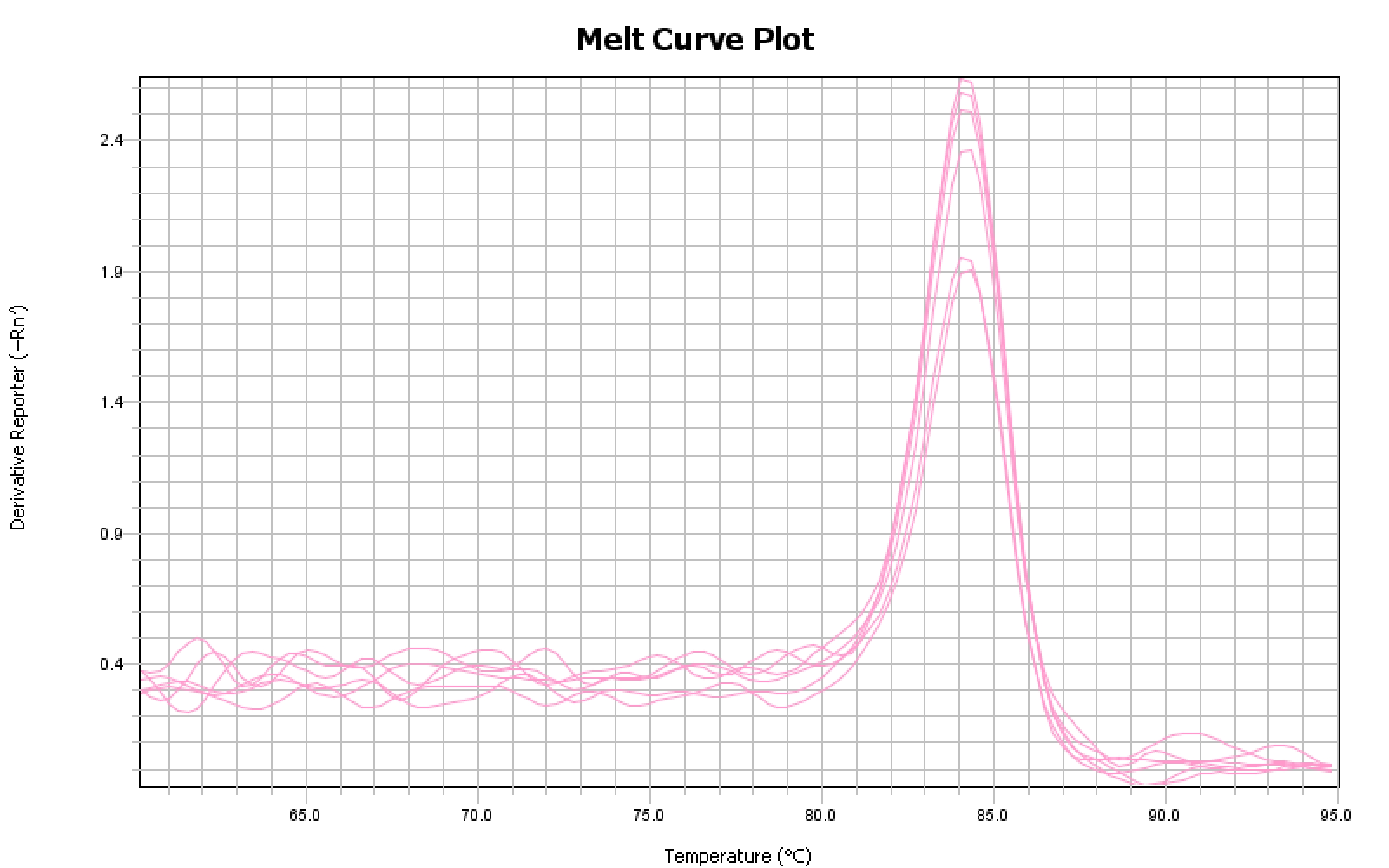

Supplement: Supplementary file 2 [file DataSheet_2.zip › Original Data 2/Figure S3B/shNLE1+CDK1/NLE1/Melt Curve Plot H-NLE1.jpg]
