# Supplementary material for: Identification of NLE1/CDK1 axis as key regulator in the development and progression of non-small cell lung cancer
Source: Front Oncol. 2023 Feb 1;12:985827. doi: 10.3389/fonc.2022.985827 (PMC9931185; doi:10.3389/fonc.2022.985827)
Supplement: Supplementary file 2 [file DataSheet_2.zip › Original Data 2/Figure 4C/A549/shCtrl+AKT-1.pdf]

Well Number: E05 Sample ID: E05

File Name: //192.168.1.121/正在处理/华东套餐组（石艳娇 汪波 丁婕 高仁杰 纪文超 蔡阳）/015 高仁杰/GRJ/凋亡周期/20210813 549 LIUZU/2

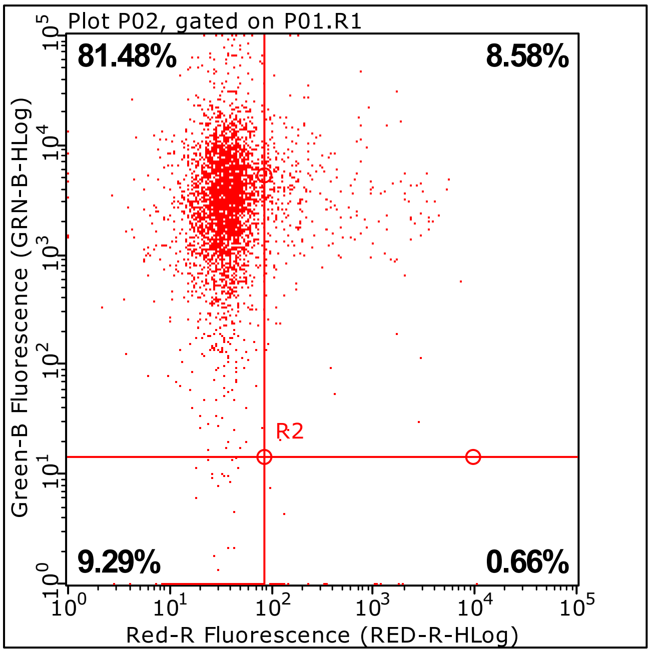

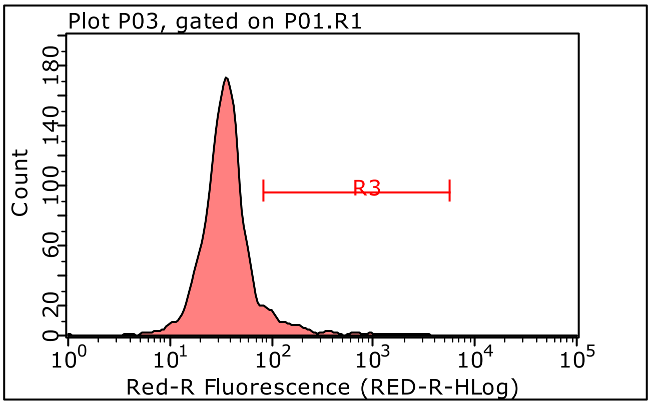

Well	Sample ID	Date	R2.Percent.UL Percent for R2 gated by P01.R1 (%)	R2.Percent.UR Percent for R2 gated by P01.R1 (%)	R2.Percent.LL Percent for R2 gated by P01.R1 (%)
E05	E05	08.22.2021	81.48	8.58	9.29

Well	R2.Percent.LR Percent for R2 gated by P01.R1 (%)	R3.Percent Percent for R3 gated by P01.R1 (%)
E05	0.66	9.31
