# Supplementary material for: Identification of NLE1/CDK1 axis as key regulator in the development and progression of non-small cell lung cancer
Source: Front Oncol. 2023 Feb 1;12:985827. doi: 10.3389/fonc.2022.985827 (PMC9931185; doi:10.3389/fonc.2022.985827)
Supplement: Supplementary file 2 [file DataSheet_2.zip › Original Data 2/Figure 4C/NCI-H1299/shNLE1+AKT-2.pdf]

Well Number: F10 Sample ID: F10
File Name: //192.168.1.121/正在处理/华东套餐组（石艳娇 汪波 丁婕 高仁杰 纪文超 蔡阳）/015 高仁杰/GRJ/凋亡周期/20210807 1299/2021-

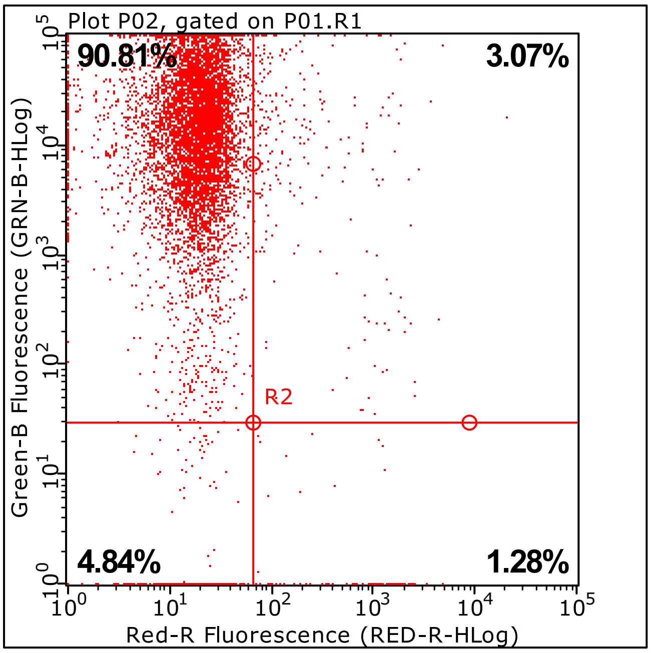


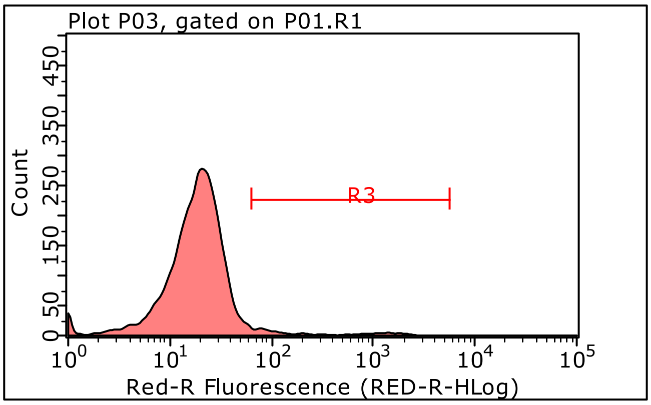

| Well | Sample ID | Date | R2.Percent.UL Percent for R2 gated by P01.R1 (%) | R2.Percent.UR Percent for R2 gated by P01.R1 (%) | R2.Percent.LL Percent for R2 gated by P01.R1 (%) |
|---|---|---|---|---|---|
| F10 | F10 | 08.22.2021 | 90.81 | 3.07 | 4.84 |

| Well | R2.Percent.LR Percent for R2 gated by P01.R1 (%) | R3.Percent Percent for R3 gated by P01.R1 (%) |
|---|---|---|
| F10 | 1.28 | 4.36 |
