# Supplementary material for: Identification of NLE1/CDK1 axis as key regulator in the development and progression of non-small cell lung cancer
Source: Front Oncol. 2023 Feb 1;12:985827. doi: 10.3389/fonc.2022.985827 (PMC9931185; doi:10.3389/fonc.2022.985827)
Supplement: Supplementary file 2 [file DataSheet_2.zip › Original Data 2/Figure 5E/Control-2.pdf]

Well Number: H02 Sample ID: H02
File Name: E:/HY/1/2020-04-17_at_06-05-16pm.fcs

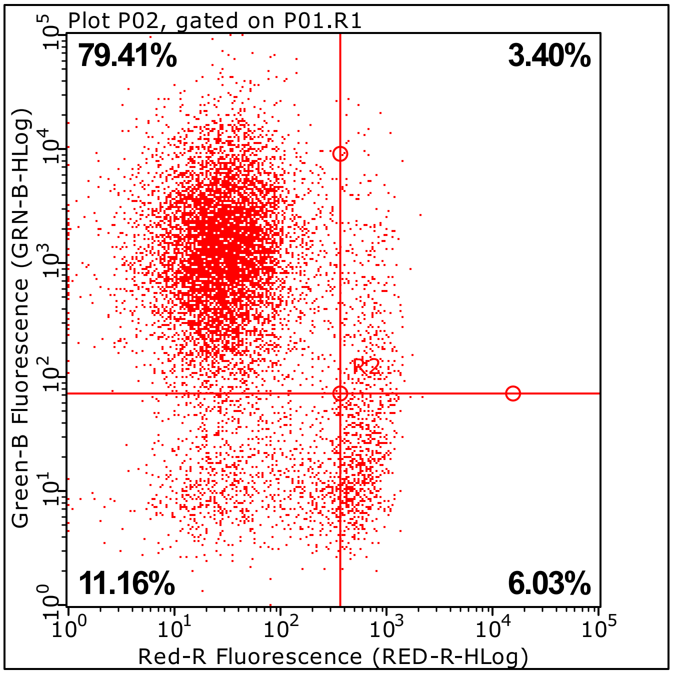

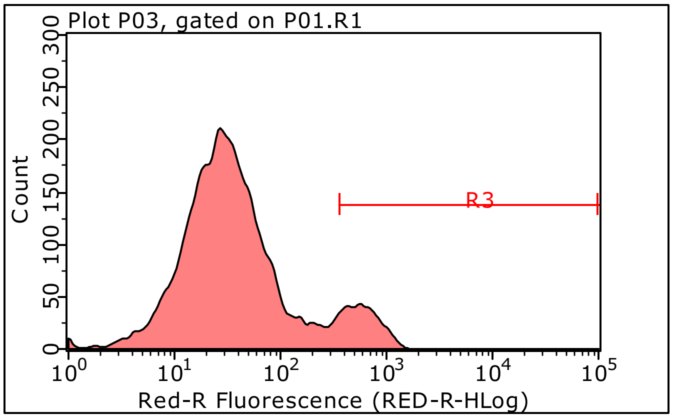

Well	Sample ID	Date	R2.Percent.UL Percent for R2 gated by P01.R1 (%)	R2.Percent.UR Percent for R2 gated by P01.R1 (%)	R2.Percent.LL Percent for R2 gated by P01.R1 (%)	R2.Percent.LR Percent for R2 gated by P01.R1 (%)
H02	H02	04.14.2020	79.41	3.40	11.16	6.03

Well	R3.Percent Percent for R3 gated by P01.R1 (%)
H02	9.46
