# Supplementary material for: Identification of NLE1/CDK1 axis as key regulator in the development and progression of non-small cell lung cancer
Source: Front Oncol. 2023 Feb 1;12:985827. doi: 10.3389/fonc.2022.985827 (PMC9931185; doi:10.3389/fonc.2022.985827)
Supplement: Supplementary file 2 [file DataSheet_2.zip › Original Data 2/Figure 5E/NC(KD+OE)-3.pdf]

Well Number: A11 Sample ID: A11
File Name: E:/tp/202003/New folder/2020-03-20_at_02-43-56pm.fcs

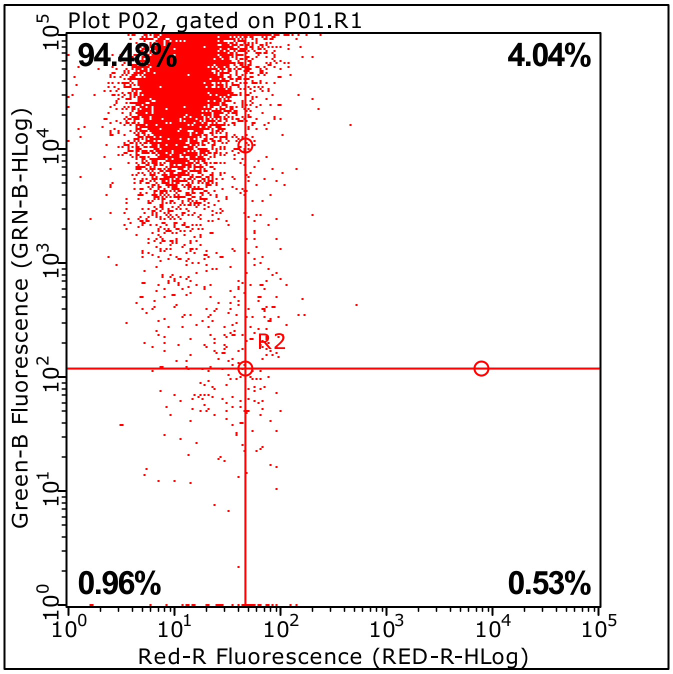

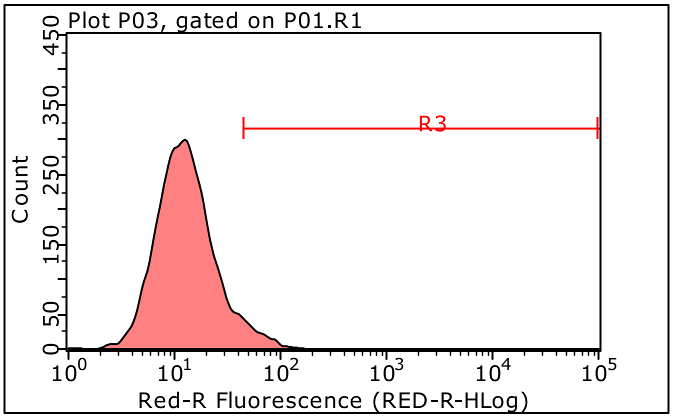

Well	Sample ID	Date	R2.Percent.UL Percent for R2 gated by P01.R1 (%)	R2.Percent.UR Percent for R2 gated by P01.R1 (%)	R2.Percent.LL Percent for R2 gated by P01.R1 (%)	R2.Percent.LR Percent for R2 gated by P01.R1 (%)
A11	A11	03.24.2020	94.48	4.04	0.96	0.53

Well	R3.Percent Percent for R3 gated by P01.R1 (%)
A11	4.56
