# Supplementary material for: Identification of NLE1/CDK1 axis as key regulator in the development and progression of non-small cell lung cancer
Source: Front Oncol. 2023 Feb 1;12:985827. doi: 10.3389/fonc.2022.985827 (PMC9931185; doi:10.3389/fonc.2022.985827)
Supplement: Supplementary file 3 [file Image_1.pdf]

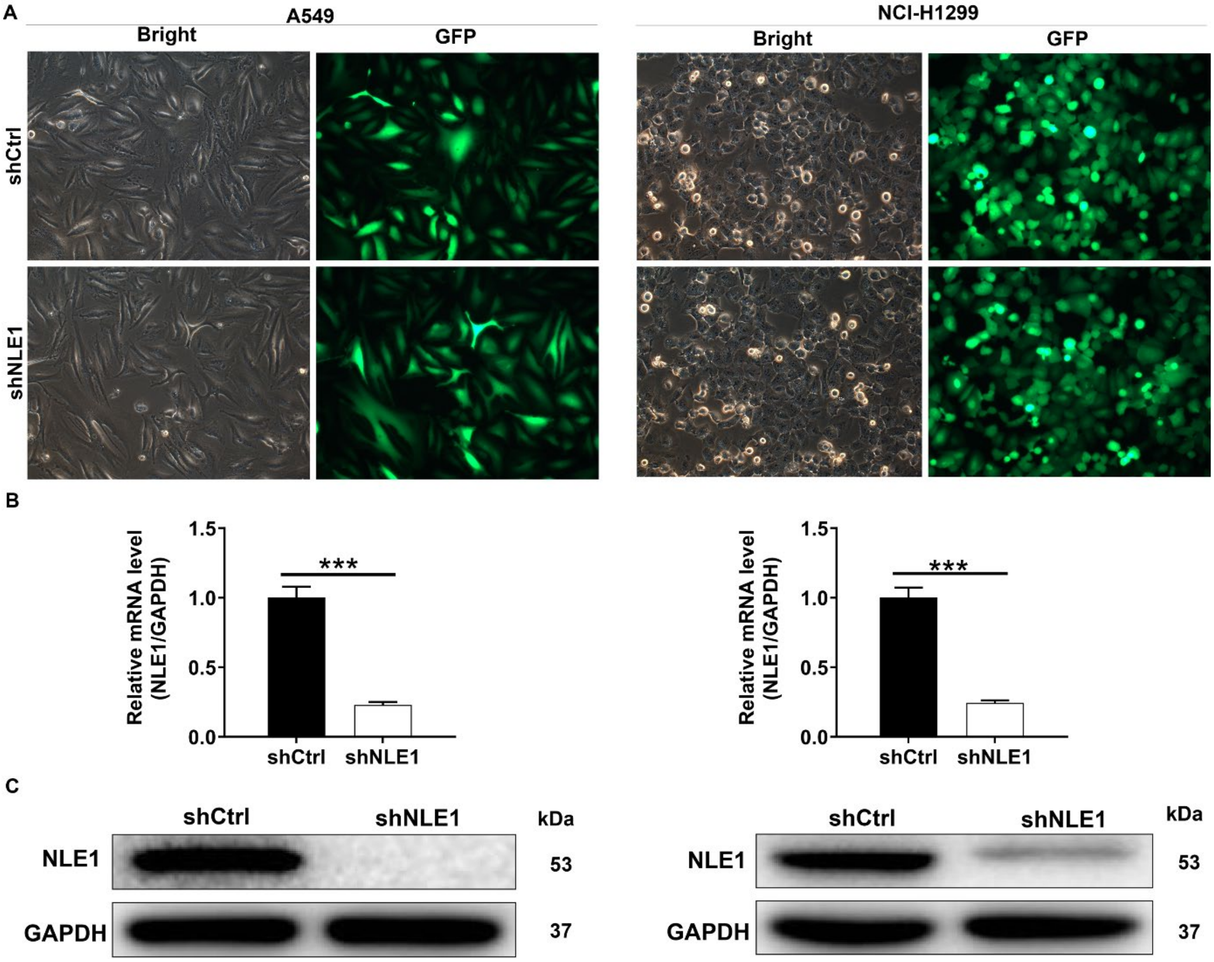

Figure S1. (A) The infection efficiencies of shCtrl and shNLE1 in A549 and NCI-H1299 cells were evaluated by observing the fluorescence of GFP. Magnification times: 200×. (B, C) The knockdown efficiencies of shCtrl and shNLE1 in A549 and NCI-H1299 cells were evaluated by qRT-PCR assay (B) and western blot analysis (C). Results were presented as mean ± SD. *** $P < 0.001$.

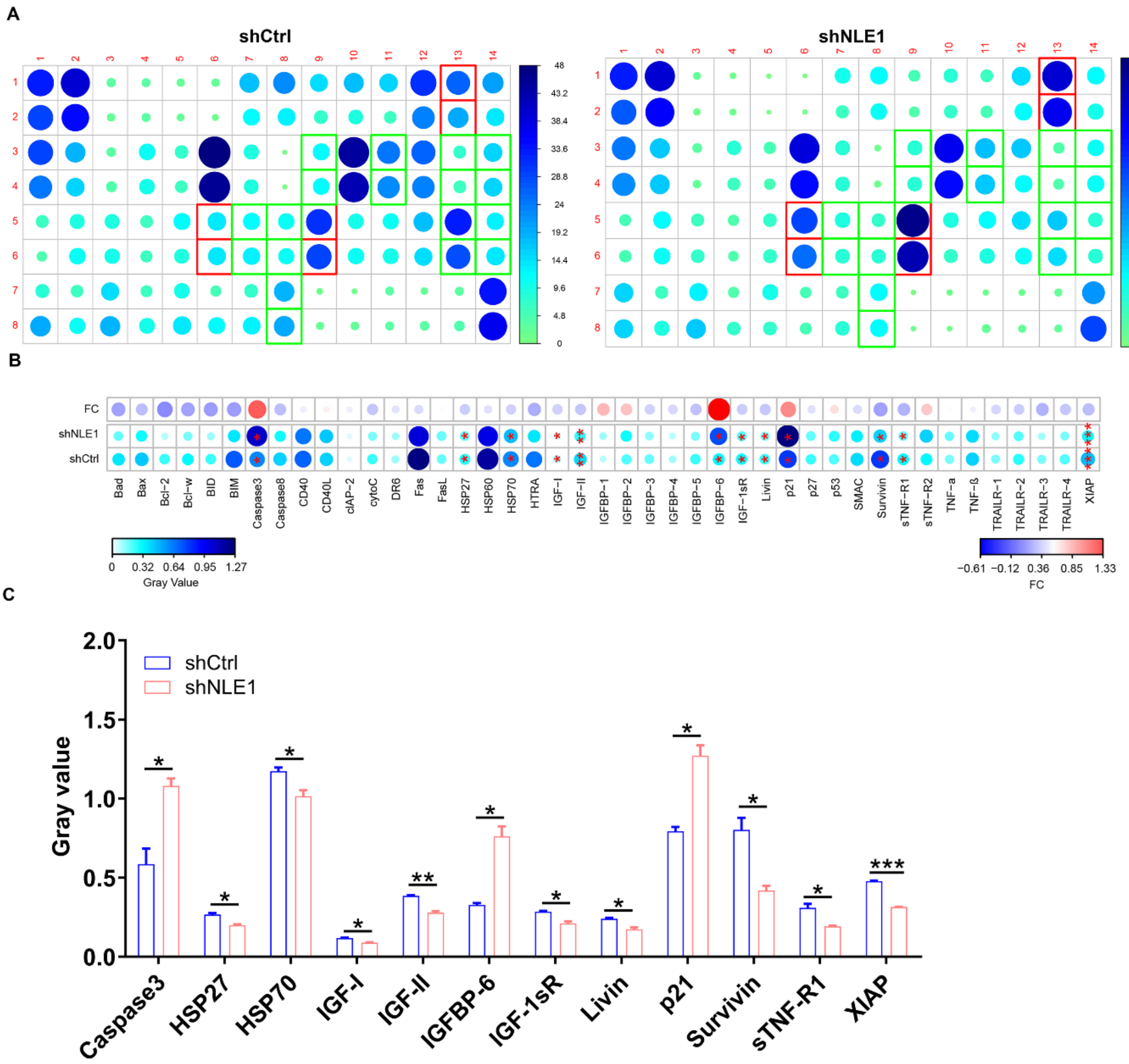

Figure S2. (A) The expression of apoptosis-related proteins was measured in NCI-H1299 cells infected with shCtrl and shNLE1 by ECL with Human Apoptosis Antibody Array. The results circled in red indicated that the protein expression was up-regulated and $P < 0.05$. (B, C) Protein expression was visualized by R studio (B) and presented in gray value (C). Results were presented as mean ± SD. * $P < 0.05$, ** $P < 0.01$, *** $P < 0.001$.

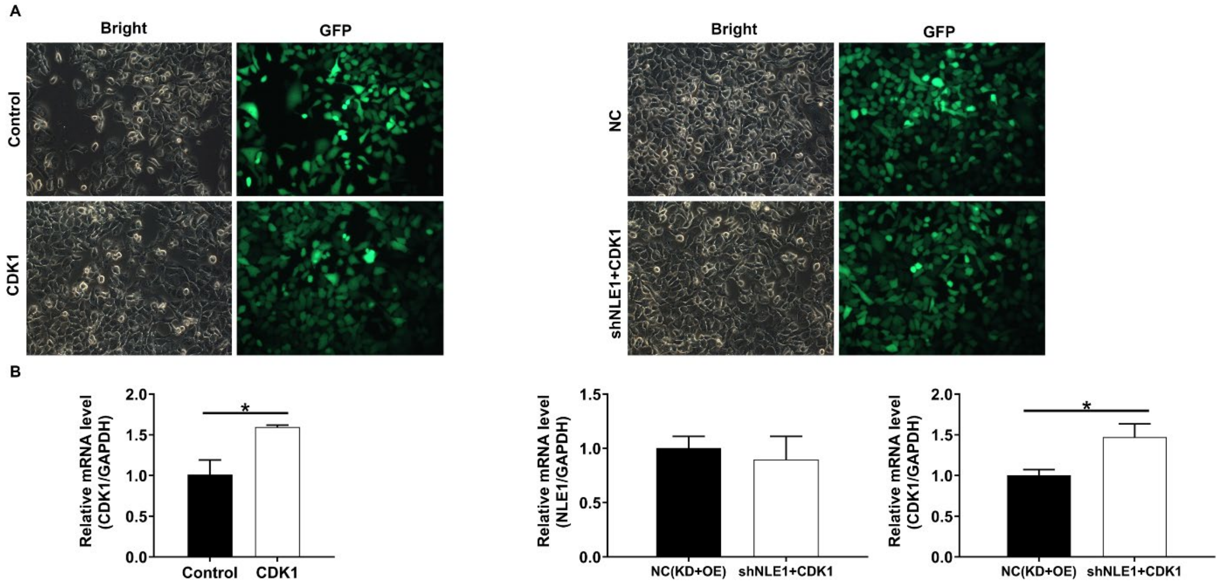

Figure S3. (A) The infection efficiencies of CDK1 and CDK1+shNLE1 in NCI-H1299 cells were assessed through observing the fluorescence of GFP. Magnification times: 200×. (B) The overexpression or knockdown efficiencies of CDK1 and CDK1+shNLE1 in NCI-H1299 cells were assessed through qRT-PCR assay. Results were presented as mean ± SD. * $P < 0.05$.
